# Uncertainties in global hydrological and climate models challenge future estimates of crop water use and sustainability

**DOI:** 10.1038/s43247-026-03621-w

**Published:** 2026-05-26

**Authors:** Qiming Sun, Francesca Bassani, Marta Tuninetti, Sara Bonetti

**Affiliations:** 1 Laboratory of Catchment Hydrology and Geomorphology, École Polytechnique Fédérale de Lausanne (EPFL), Sion, Switzerland; 2 Department of Environment, Land and Infrastructure Engineering, Politecnico di Torino, Torino, Italy

**Keywords:** Hydrology, Governance, Water resources

## Abstract

Assessing crop water requirements and sustainability under future climates is crucial to concurrently address water scarcity, food security, and ecosystem preservation. Yet, global climate and hydrological models differ substantially in estimating precipitation, potential evapotranspiration, and renewable freshwater, challenging future crop water use and sustainability assessments. Here, we perform a multi-model analysis to quantify how model uncertainty propagates into crop water needs and sustainability estimates. Results show end-of-century global uncertainty spreads of ~ 18% and ~ 51% for green and blue water footprints, respectively, relative to ensemble means. Blue water sustainability exhibits even larger variability, from 250% to 451%, depending on scenario and model choices. These uncertainties further intensify at finer spatial scales. Hydrological model discrepancies dominate blue water assessments, whereas climate model variability primarily affects green water estimates, particularly under high-emission scenarios. Accounting for model uncertainty is essential for reliable crop water estimates and to inform agricultural water management under climate change.

## Abstract (web summary)

Model uncertainty causes wide divergence in future estimates of crop green and blue water footprints (~ 18–51%) and water debts (~ 250–451%), challenging reliable assessment of crop water use and sustainability, according to a global, multi model climate-hydrology analysis of end century scenarios.

## Introduction

Agriculture is the dominant consumer of freshwater resources, accounting for nearly 70% of global withdrawals worldwide^1^. Demand for water in agriculture is projected to increase further as population growth, dietary shifts, and expanding international trade drive higher food production requirements^2^. Meeting these rising anthropogenic demands places growing pressure on finite freshwater resources^3^. Climate change further compounds this challenge by intensifying extremes and increasing the uncertainty of water supply^4,5^.

Assessing and managing the sustainability of crop water use is therefore essential to cope with predicted water scarcity^6–12^, ensure global food security^2,5,13^, and inform sustainable water management^14,15^ under climate change. In this context, a growing body of literature has focused on the assessment of crop water requirements and sustainability^16–19^, and various metrics have been developed for their quantification, with applications ranging from local and national level assessments^20–22^ to global studies^23–26^. Common approaches include risk-based indicators^27^ and threshold-based classifications of water stress^28^. The majority of these indicators are effective for broad-scale assessments, but they often lack a direct physical basis and are limited in their ability to attribute water use and impact to individual crops within a given area^29^. To overcome such limitations and account directly for crop water use, two metrics have been proposed, namely the water footprint (WF), defined as the total volume of freshwater used to produce a certain crop^30–32^, and the water debt repayment time (WD), which is the time required to replenish water resources used for annual crop production^29^. While WF quantifies the absolute magnitude of water use per year (e.g., in m^3^ yr^−1^), WD relates this consumption to local hydrological renewability rates, translating volumes into a timescale that indicates whether crop water use exceeds the rate at which resources are naturally replenished on a yearly basis. Each of these indicators can be further assessed based on the different sources of water used by crops, namely the irrigated quota from surface water bodies and groundwater (“blue water”) and soil moisture (“green water”).

For the quantification of water use indicators, such as WF, several climatic and hydrologic inputs are needed, particularly estimates of precipitation and potential evapotranspiration (PET). Water sustainability indicators, such as WD, further require knowledge of renewable freshwater rates, including surface runoff, groundwater recharge, and soil moisture. All these variables can be derived from global climate and hydrological (water) models (GCMs and GWMs, respectively), which typically provide both historical observations and future projections for a range of future scenarios and different socio-economic assumptions^33^. However, these models often yield divergent estimates of the aforementioned variables due to multiple sources of uncertainty along the climate-hydrology modeling chain. In this study, uncertainty is understood as the incomplete and imperfect information about climate forcings and hydrological processes, which leads to probabilistic rather than deterministic estimates of crop evapotranspiration and water use. A major component is epistemic uncertainty, arising from differences in model structures, assumptions, and parameterizations, resulting in varying estimates of relevant hydrologic variables^34,35^. Additional uncertainty stems from the spread in climate projections^36^, reflecting both epistemic uncertainty related to various climate model formulations and scenario uncertainty associated with future socio-economic trajectories. Moreover, the inherently chaotic nature of the climate system contributes to internal variability, further compounding these uncertainties^4,37^. For crop water use indicators, these uncertainties propagate into output estimates, as demonstrated in water footprint and crop water use studies at basin to global scales^22,26^. This uncertainty is critical because it directly affects the reliability of sustainability assessments: overestimating water availability or underestimating crop water demands may lead to overly optimistic conclusions, while ignoring spread in model predictions may conceal regions facing critical water stress or unsustainable water use. Explicitly accounting for these uncertainties can support more robust and informative estimates of crop water use, enabling better-informed water management decisions.

This study aims to quantify how uncertainties embedded in estimates from global climate and water models propagate into crop water use and sustainability assessments from local to regional and global scales. To this aim, following a methodology proposed in previous studies^29,38^, we compute water requirements (blue and green WFs) and sustainability indicators (blue and green WDs) at fine spatial resolution (on a grid of 5 arc-min resolution, corresponding to about 9 km at the equator) at the global scale. The analysis is performed for eight of the most produced crops worldwide (rice, wheat, maize, potato, soybean, sugarcane, barley, and sorghum), and both for the reference year 2000 (for which most agricultural mapped datasets were available at the time of the study) as well as for future horizons. Specifically, WF and WD are evaluated every 10 years from 2010 to 2090, considering three Representative Concentration Pathways (RCPs), namely 2.6, 6.0, and 8.5, illustrative of different global warming trajectories. Estimates of precipitation, reference evapotranspiration, and renewability rates needed to solve the soil moisture balance in the model and compute WF and WD were obtained from six GWMs and four GCMs (ISIMIP2b^33^, see Methods). Performing a multi-model analysis allows us to quantify the uncertainties in the estimation of indicators for crop-water requirements and sustainability associated with different GCMs and GWMs, their propagation in future scenarios and across spatial scales of analysis, as well as to pinpoint the major sources of uncertainty and their spatial distribution. Characterizing this ensemble uncertainty is essential to assess the robustness and likelihood of projected changes in water sustainability, beyond the outcomes of individual models. In this context, our work further enables the identification of hotspot regions and crops that are likely to experience severe transitions to unsustainable water use in the future. Quantifying uncertainties embedded in crop water use and sustainability estimates and tracing them back to their main sources is crucial for providing a clear understanding of potential risks, improving future predictions, and enabling the formulation of targeted strategies to manage water resources sustainability in the face of climate change.

## Results

### Variability in multi-model projections of crop water requirements and sustainability

**Fig. 1:**
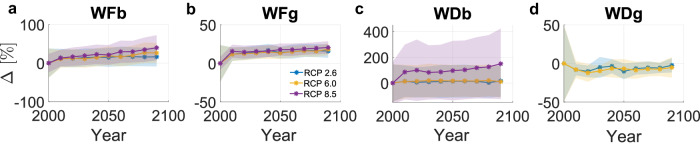
Variability in projections of global water footprint and water debt estimates. Temporal evolution of the percentage change *Δ* (relative to the average in the reference year 2000) of global (**a**) blue water footprint WF_b_, (**b**) green water footprint WF_g_, (**c**) blue water debt WD_b_, and (**d**) green water debt WD_g_ accounting for all the 8 crops (crop-specific graphs are provided in Supplementary Fig. S5). Solid lines represent average values across all GWMs and GCMs under RCP 2.6 (blue), 6.0 (yellow), and 8.5 (purple). Shaded areas denote the uncertainty range computed as the standard deviation among values from all models. WD_g_ is not available under RCP 8.5 (see Table 1).

Global projections show an increase in future global blue and green water footprints (WF_b_ and WF_g_, respectively) across all climate scenarios, with respect to their global averages in the reference year 2000 (WF_b_= 638 km^3^ yr^−1^, and WF_g_= 2856 km^3^ yr^−1^, accounting for all the eight crops). Specifically, for WF_b_, the ensemble means across all models increase by 16% and 26% under RCP 2.6 and 6.0, respectively, and up to 39.7% under RCP 8.5 by 2090 relative to the year 2000 (solid lines in Fig. 1a), indicating higher water demands in scenarios with more severe climate change, as expected. The uncertainty range broadens over time, reflecting diverging model responses. WF_g_ exhibits a narrower range of uncertainty (average inter-model variability across all RCPs of about  ± 9%, Fig. 1b, approximately 2.8 times lower than the inter-model variability for WF_b_), suggesting more consistent model predictions for green water use compared to WF_b_. WD_b_ exhibits far greater inter-model spread than WF metrics (see also crop-specific values in Supplementary Fig. S1), with an average spread around the mean across all years (Fig. 1c) of 250% (RCP 2.6), 254% (RCP 6.0) and 451% (RCP 8.5) (Supplementary Tables S1–S4). Divergences mainly arise from differences in projected surface runoff (Q_r_) and groundwater recharge (Q_s_) estimates from GWMs, with MATSIRO providing, on average, the highest Q_r_, PCR-GLOBWB and CWatM yielding the lowest Q_s_, and LPJmL exhibiting the strongest temporal variability (Supplementary Figs. S2–S3). We further note that the ensemble mean (across all models) of WD_b_ only shows a significant increasing trend (Mann-Kendall test^39,40^, *p* < 0.05, Table S5) under the most severe climate scenario (RCP 8.5), while remaining relatively constant under RCPs 2.6 and 6.0 (Fig. 1c). No statistically significant trends emerge for individual models under these scenarios (Supplementary Fig. S4 and Supplementary Table S5). Despite relying on only two GWMs with available root-zone soil moisture data (Table 1), WD_g_ shows significantly lower variability at the global scale than WD_b_ (± 26% and ± 29% on average, depending on the RCP – see Fig. 1d). The temporal evolutions of global WF and WD for specific crops further highlight those crops whose WF and WD estimates exhibit the greatest variability across models (namely barley, maize, soybean, wheat, and rice) (Supplementary Fig. S5). This is ascribable to their concentration in areas where differences in GWM and GCM responses are more significant, as further discussed in the following.

**Table 1:** Summary of available variables across water models and scenarios

	Potential evapotranspiration				Blue water variables: runoff and groundwater recharge				Green water variable: root zone soil moisture			
Scenario →	h	2.6	6.0	8.5	h	2.6	6.0	8.5	h	2.6	6.0	8.5
CWatM	h_1_	f_05_	f_05_	f_05_	h_1_	f_05_	f_05_	f_05_	h_1_	f_05_	f_05_	f_05_
PCR-GLOBWB	h_1_	f_05_	f_05_	–	h_1_	f_05_	f_05_	–	h_1_	f_05_	f_05_	–
LPJmL	h_1_	f_05_	f_05_	f_05_	h_1_	f_05_	f_05_	f_05_	–	–	–	–
H08	h_1_	f_05_	f_05_	f_05_	h_1_	f_05_	f_05_	f_05_	–	–	–	–
WaterGAP2	h_1_	f_05_	f_05_	f_05_	h_1_	f_05_	f_05_	f_05_	–	–	–	–
MATSIRO	h_1_	f_05_	f_05_	–	h_1_	f_05_	f_05_	f_05_*	–	f_05_	f_05_	–

Summary of available variables for each GWM (first column) and scenario (h refers to historical, while the numbers to the corresponding RCP scenario), derived from ISIMIP2b^33^. Dash symbols imply data not available at the time the study was conducted (hence, no WF/WD calculations were performed for this GWM-scenario combination). “h_1_” refers to the historical impact simulations (1861-2001), while “f_05_” refers to the future RCP scenarios with fixed socio-economic conditions as of the year 2005. The superscript “*” indicates that the variables are available on ISIMIP2b, but were neglected in this study due to the unavailability of the potential evapotranspiration for the same scenario (RCP 8.5 in this case), which is the necessary condition to run the soil water balance model for crops.

**Fig. 2:**
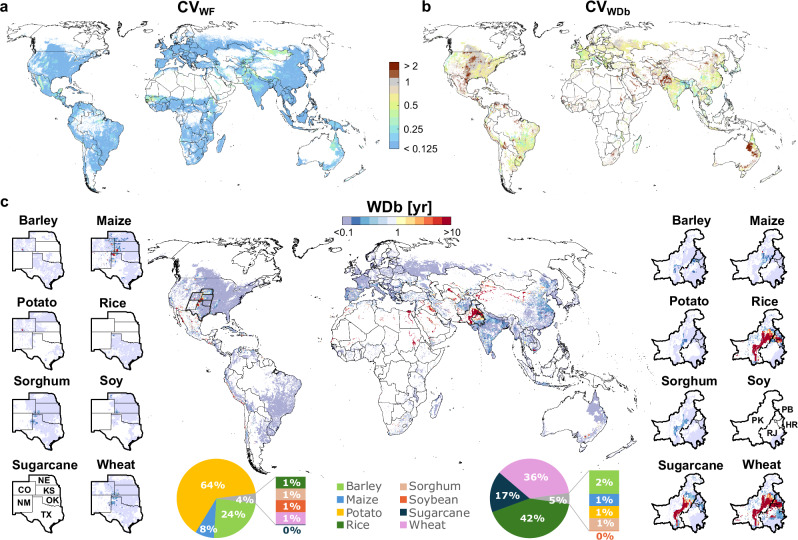
End-of-century spatial distribution of coefficient of variation and blue water debt. Coefficient of variation *CV* of total water footprint (WF=WF_b_+WF_g_) (**a**) and blue water debt WD_b_ (**b**) for the year 2090 (RCP 8.5). **(c)** Blue water debt (WD_b_) accounting for all crops and averaged across all climate and water models (year 2090, RCP 8.5). Solid black boundaries highlight two hotspot regions (WD_b_ > 10 years) for which crop-specific WD_b_ values are shown on the left side (Nebraska (NE), Kansas (KS), Oklahoma (OK), Texas (TX), New Mexico (NM), Colorado (CO), United States) and right side (Pakistan (PK); Indian provinces of Punjab (PB), Haryana (HR), and Rajasthan (RJ)). Pie charts show percentage contributions of each crop to WD_b_. High percentages of potato and barley in the USA derive from local WD_b_ ≫ 10 years in Colorado. Credit: all maps were produced using m_map and NOAA National Centers for Environmental Information shapefiles.

To pinpoint specific locations where WF and WD assessments may be more unreliable, we map the spatial distribution of the projected coefficient of variation, *CV*, in 2090 (RCP 8.5) (Fig. 2a, b). *CV* is defined as the ratio between the variability (standard deviation) across all model estimates and the corresponding multi-model mean (Methods). Therefore, values of *CV* < 1 indicate that the ensemble spread is smaller than the mean signal, implying that model agreement is relatively strong, while *CV* > 1 denotes that the variability across models exceeds the mean, and thus low agreement and higher uncertainty. For WF, most regions exhibit *CV* ≪ 1 (Fig. 2a), indicating a relatively low level of uncertainty compared to the variable’s magnitude and suggesting that results are broadly robust, as the uncertainty does not dominate the signal. In contrast, WD_b_ exhibits higher *CV* values, reflecting the much larger variability of the sustainability metric already highlighted above. This difference arises from the formulation of WD_b_ as the ratio between water demand and blue renewability rate. In fact, while WF primarily depends on precipitation and potential evapotranspiration, which are relatively consistent across models (Supplementary Figs. S6–S8), WD_b_ is strongly influenced by runoff and groundwater recharge, which exhibit large inter-model differences due to divergent process representations in hydrological models^41^. As a result, variability in WD_b_ across GWMs might exceed the magnitude of the mean signal itself, leading to *CV* > 1 in certain regions, such as Eastern Australia, North-Western India, and Central USA (Fig. 2b), thus highlighting areas where crop water estimates are particularly sensitive to model choice (see also Supplementary Tables S1–S4 for crop-specific global values of WF and WD_b_, and Supplementary Figs. S9–S12 for the corresponding maps).

A closer look at the spatial distribution of projected WD_b_ further highlights that a subset of such regions is also characterized by significant pressure on water resources, and thus, high values of WD_b_ (Fig. 2c). These hotspots emerge across all RCP scenarios (Supplementary Fig. S13) and correspond to areas with the highest rates of groundwater depletion^42^, where local and regional aquifer systems are over-exploited^43^, thus affecting groundwater recharge. Significant hotspots, for instance, are observed in the Central USA and along the Pakistan-India border, where the dominant crops in each region contribute to exceeding sustainable levels (potatoes, barley, and maize in the former; wheat, rice, and sugarcane in the latter; see insets in Fig. 2c). The positive but relatively weak correlation between WD_b_ and *CV*(WD_b_) (slope of the linear fitting  < 0.05, Supplementary Fig. S14) reflects the fact that, while high water debt is associated with higher uncertainty, such conditions are confined to spatially limited hotspots.

The granularity of these results hints to a scale-dependency of uncertainty, thus motivating a closer examination of how uncertainty varies ranging from global averages to the local grid cell estimates. To this aim, we compute the *CV* for WF and WD variables to quantify how uncertainties change with spatial resolution (see Methods). For WF_b_ and WF_g_, *CV* increases at finer scales, indicating a considerably higher divergence in model estimates at the single-cell level than in aggregated global-scale means (Supplementary Fig. S15). This occurs because local-scale projections retain the full magnitude of inter-model differences, whereas aggregation across larger areas averages out opposing deviations, leading to lower apparent uncertainty at coarser scales, as also noted in hydrological impact studies^44^. In contrast, WD_b_ and WD_g_ exhibit an opposite behavior. Here, the heavy-tailed distribution of cell-level spread means that a few hotspots exhibit extremely high values (see Fig. 2c). As the averaging domain expands, the likelihood of including such hotspots rises, thus increasing the block-level uncertainty.

### Disentangling the relative contributions of global climate and water models

The preceding analyses highlight substantial variability across different GCM and GWM combinations. Understanding the origins of this variability is crucial to determine the robustness of crop water use and sustainability estimates. We therefore turn to disentangling the relative contributions of GCMs and GWMs to the total variability across scales and locations.

**Fig. 3:**
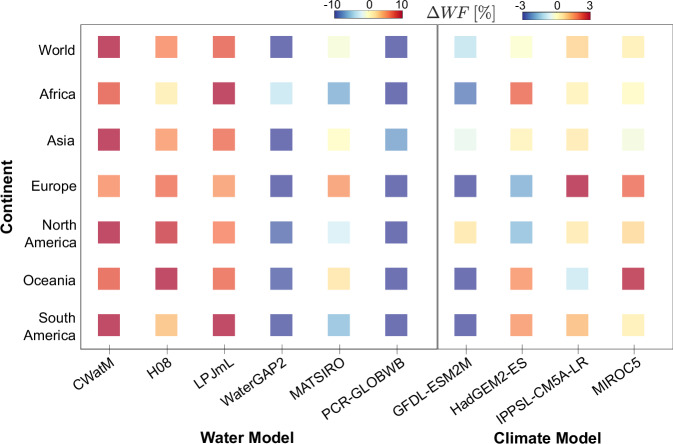
Sources of uncertainty in global and continental scale crop water footprint. Variability of global and continental scale total water footprint WF (blue plus green) across GWMs (left panel) and GCMs (right panel) averaged for all years between 2000 and 2090 and all crops (here future projections refer to RCP 6.0, for which all models are available, see Table 1). Colors code the relative deviation of WF with respect to the average value per continent *C*: $\Delta {{{\rm{WF}}}}=[({{{{\rm{WF}}}}}_{C,m}-{\overline{{{{\rm{WF}}}}}}_{C})/{\overline{{{{\rm{WF}}}}}}_{C}]\cdot 100$, *m* being the considered model. The same analysis for WD_b_ is shown in Supplementary Fig. S18.

At the global and continental scales (Fig. 3), the largest variability in total WF (considering blue and green contributions together) arises from GWMs, which provide PET inputs to the soil water balance model (see Methods). Deviations from the continental mean approach +10% for CWatM, H08, LPJmL, and  − 10% for WaterGAP2 and PCR-GLOBWB (positive/negative drifts indicate over/under-estimations with respect to the continental average) under RCP 6.0. Differences among GCMs, linked to precipitation forcings (Supplementary Fig. S6, coefficient of variation *CV* = 3.4% globally), are smaller in magnitude.

The largest variability among GWMs is ascribable to PET estimates, which explain much of this divergence (Supplementary Figs. S7–S8). In particular, in year 2000, MATSIRO simulates PET up to 70% below the multi-model mean, resulting in a global WF_b_ 76% lower than average (Supplementary Fig. S4). This is also visible in Fig. 1, where PET estimates from MATSIRO induce significantly large discrepancies in water footprints only for year 2000. WaterGAP2 and PCR-GLOBWB systematically yield PET estimates approximately 20% below the mean (Supplementary Figs. S7–S8), yielding WF_b_ reductions of between 26 and 28% depending on the scenario (Supplementary Fig. S4). Their probability distributions are consistently shifted toward lower values across all years and RCPs (Supplementary Figs. S16–S17).

While uncertainty in global WF_b_ (*CV* ≃ 23%) primarily arises from variability in PET (*CV* = 15.4%), WD_b_ shows substantially larger deviations from the ensemble mean (up to  ± 150% across GWMs, Supplementary Fig. S18). This occurs because WD_b_ estimates integrate not only PET variability but also uncertainties in water renewability rates. The relatively minimal variability in blue renewability rates among GWMs (*CV* = 14% based on global values) points to a highly non-linear relationship, indicating that quantifying the uncertainty in input variables alone is insufficient to capture the total uncertainty that propagates through the modeling chain into the final water sustainability indicator.

**Fig. 4:**
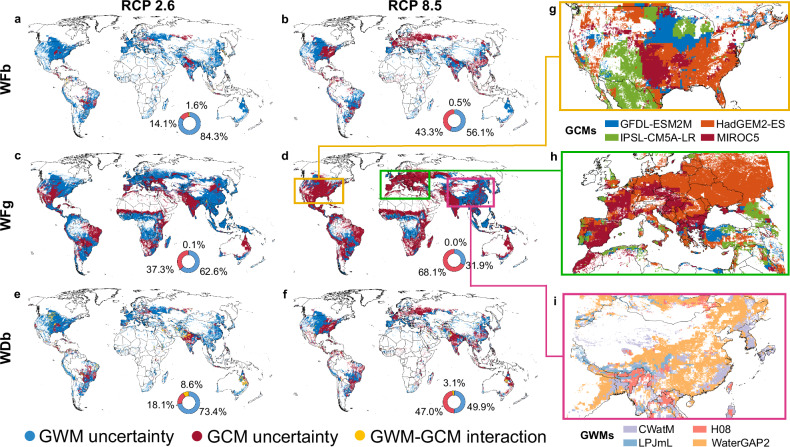
Dominant sources of uncertainty. Global scale dominant source of uncertainty, deriving from water models (GWM, in blue), climate models (GCM, in red), or GWM-GCM interactions (in yellow), for blue water footprint WF_b_ (**a**, **b**), green water footprint WF_g_ (**c**, **d**), and blue water debt WD_b_ (**e**, **f**). The global maps refer to the year 2090, under RCP 2.6 (first column) and RCP 8.5 (second column). Doughnut charts show the global area proportion of each source (Methods). The three insets associated with the RCP 8.5 projections of WF_g_ feature macro-areas for which the dominant source of uncertainty is further examined by highlighting the GCMs (**g**, **h**) and GWMs (**i**) that contribute the most to the local uncertainty. Results are based on four climate models for all RCPs, combined with six GWMs for RCP 2.6 and four GWMs for RCP 8.5. The complete list of GCMs and GWMs is provided in the Methods (Table 1). Credit: all maps were produced using m_map and NOAA National Centers for Environmental Information shapefiles.

Moving to more local scales, we now turn our attention to identifying the dominant sources of uncertainty (GCMs or GWMs) across global cropland areas. Figure 4 shows the projected end-of-century global distribution of major uncertainty sources for RCPs 2.6 and 8.5. Disagreement across water models dominates both WF_b_ and WD_b_ uncertainties, in line with previous studies^45^ (and as already emerged in Fig. 3 and in Supplementary Fig. S18). However, under RCP 8.5, the key role of GCM uncertainty grows: climate models control 43.3% of WF_b_ uncertainty mostly across Central Europe, Eastern USA, and southern Asia (Fig. 4b), and 47% of WD_b_ uncertainty in more scattered locations across global croplands, with no other major pronounced regional clustering apart from some regions in China and India (Fig. 4f). For WF_g_, the dominant source shifts from GWMs under RCP 2.6 (Fig. 4c) to an opposite climate-controlled uncertainty under RCP 8.5 for 68.1% of global cropland areas (Fig. 4d). Notably, most of the uncertainty can be attributed to the main effects of GWMs and GCMs, with only a minor contribution arising from their combined (non-linear) interactions (see Methods). Indeed, the interaction term remains limited across all variables and scenarios (yellow shares in Fig. 4), with the highest interaction-driven contribution found for WD_b_ under RCP 2.6 (8.6%), decreasing to 3.1% under RCP 8.5 (Fig. 4e, f). To further dig into the merits of these results, we investigated the contributions of each climate and water model to the uncertainty for three macro-areas. For GCMs, uncertainty is mainly associated with HadGEM2-ES estimates around Eastern USA (Fig. 4g) and East Europe (Fig. 4h), while for GWMs, WaterGAP2 seems to be the main driver of uncertainty in several Asian regions (Fig. 4i).

## Discussion

These findings demonstrate the critical role that global water and climate model uncertainties play in the assessment of current and future crop water requirements and sustainability across spatial scales.

Specifically, we highlighted the different roles that variability and uncertainties in GCMs and GWMs play for water use and sustainability estimates (here quantified using WF and WD indicators), as we move from current conditions into future scenarios. Uncertainty in crop water use estimates (here embedded in WF) is dominated by the choice of GWMs, which provide divergent estimates of PET in terms of both global average annual values and their spatial variability (see Supplementary Figs. S7–S8). Conversely, while the four climate models considered here show broad agreement in precipitation estimates (both annual totals and their distributions, Supplementary Fig. S6), their discrepancies become increasingly prominent in future projections–especially under high-emission scenarios–thereby constraining the reliability of water use and sustainability projections. In deriving WD, defined as the ratio of WF to the locally available water resources (local renewability rates), these uncertainties are substantially magnified, reflecting the considerable divergence in renewability projections across GWMs.

Such variability reflects structural differences among global climate and water models, largely stemming from different model structures^45–48^. In particular, the representation of CO_2_ physiological effects on vegetation (such as stomatal conductance and evapotranspiration), is not consistent across the GWMs considered here^37,49,50^ – an aspect that may further contribute to the spread in projected PET and renewability estimates. The significant variability observed, especially across GWMs, highlights the necessity of adopting ensemble approaches for crop water use and sustainability assessments, particularly in regions and at scales where discrepancies in precipitation, PET, and renewability rate estimates are largest.

The relative contribution of GCM and GWM uncertainty should also be interpreted in light of the characteristics of the available model ensemble. Although in this study the number of GWMs (six) exceeds the number of GCMs (four), we observed that the overall uncertainty appears robust to the ensemble composition, as indicated by the limited sensitivity to the exclusion of individual models (Supplementary Fig. S19). Model availability also varies across scenarios and variables within the ISIMIP2b framework. In particular, some GWMs do not provide the soil moisture renewability rate required to compute the green water debt for all RCPs (Table 1), which should be considered when comparing uncertainty magnitudes across scenarios and water components. Differences across GCM projections reflect both structural differences in model formulation and the contribution of internal climate variability. Further separation of these two contributions would require large ensembles from individual climate models^36^, which are not available within the ISIMIP2b framework used here.

The high spatial resolution of the analysis performed here further allowed us to identify hotspot regions (see Fig. 2) where crop water estimates are likely to be the least reliable–that is, where projections are particularly sensitive to model choice–hence calling for careful interpretation of crop water estimates in these areas. Although spatially explicit estimates of crop water use and sustainability are essential to support context-specific water management strategies, our results indicate that model disagreement amplifies markedly at finer spatial scales^51^. While global mean estimates remain relatively consistent across models, increased granularity reveals amplified uncertainty (with global average WF changing by approximately 50 to 100% at finer spatial scales, Supplementary Fig. S15), further challenging the reliability of localized crop water estimates and the robustness of global model outputs when applied to regional or local-level analyses.

In addition to characterizing uncertainty, the resulting WF and WD estimates offer robust spatial insights, enabling the identification of regions where water availability and demand are expected to intensify, and thus defining projected water-stress hotspots alongside their uncertainty ranges. Projected WD_b_ at the end of the century confirms persistent stress in the central United States, Pakistan, and north-west India – where repayment times exceed ten years in all scenarios (Supplementary Fig. S13). In these regions, water-intensive crops such as potatoes, barley, maize, wheat, rice, and sugarcane drive unsustainable water use (Fig. 2c)^29,42,43^. The unsustainability results from the interplay between local climatic conditions, the slow local renewability rates of water resources, and the predominance of certain water-intensive crops. Excessive reliance on irrigation, often drawing from already stressed surface or groundwater resources, further compounds the impact. While these projections are valuable, they should be interpreted with care, as this study relies on business-as-usual scenarios, assuming fixed socio-economic conditions at 2005 levels^33^ and not incorporating mitigation efforts or projected changes in management practices, crop production (yields, harvested areas, or growing seasons) driven by technological advancements, land reform policies, irrigation expansion, food demand, or market dynamics. In addition, crop differentiation in this study relies on fixed crop coefficients and static cropping calendars, not explicitly representing crop phenology and temporal variations in management practices. While such assumptions are common in global-scale assessments, they neglect adaptive responses of agricultural systems–such as shifts in sowing dates or cultivar choices–that can substantially influence crop growth periods and phenological trends^52^ and alter simulated crop performance compared with fixed-calendar assumptions^53^. This approach, however, allows us to detect locally critical areas which may face aggravated climate-driven stress in the future–thus deserving more in-depth investigation and careful water resource planning–while not introducing additional uncertainty also due to tentative estimations of environmental flow requirements^29,38^.

Global climate and water model outputs are paramount for the quantification of several crop water indicators, yet variability in their estimates leads to highly divergent assessments of present and future crop water use and sustainability. Careful quantification of the ensuing uncertainty is essential to support robust decision-making under uncertainty, since ignoring model spread risks conveying a delusive sense of accuracy in sustainability estimates^54^. Our results show that while multi-model means can help stabilize projections, they must be interpreted in light of the underlying epistemic uncertainty, including model limitations and the scarcity of observational data against which to validate them. Indeed, recent works emphasize that sustainability indicators (such as WD) critically depend on uncertain inputs–ranging from irrigated areas and crop water requirements to groundwater withdrawals and local renewability rates^38,45^. Expanding model ensemble use in this context, coupled with targeted efforts to reduce key data and process uncertainties, will therefore be fundamental to providing more reliable and policy-relevant insights on agricultural water use. In this sense, while the work here focused on the use of a specific model for computing crop water use^24,38^, additional uncertainty might arise based on the use of different agro-hydrological model configurations. As such, a coral computational effort aiming at evaluating crop water needs and their sustainability based on different agro-hydrological model formulations^55–58^ will be of utmost importance to further distill model (dis)agreements across space and time, particularly in major agricultural hotspots where large inter-model uncertainty complicates the design of robust local water management strategies. Ultimately, recognizing and transparently communicating uncertainty does not weaken water sustainability assessments; rather, it strengthens their credibility and reliability in guiding adaptive strategies under climate change.

## Methods

### Data

Crop-specific data at the spatial resolution of 5 × 5 arc-min and representative of the year 2000 were retrieved for the eight crops considered, namely maize, wheat, rice, barley, sorghum, sugarcane, soybean, and potato (categorized as in Portmann et al.^59^ and corresponding to approximately 55% of the global harvested area^59^).

For each crop, yield (*Y*) was taken from the EarthStat dataset^60^, which includes multiple FAO classes^61^ for maize (maize, maize for forage and silage, popcorn) and sorghum (sorghum, sorghum for forage and silage). To aggregate these subclasses, an area-weighted method was applied to calculate total yield values. In this study, the maize class “popcorn” was neglected since the yield (Y = 10^−22^ − 10^−20^ tons per hectares) was more than 20 orders of magnitude smaller than the other two classes (Supplementary Fig. S20).

Although EarthStat^60^ provides also crop-specific harvested areas, we used MIRCA2000^59,62^ for harvested areas (*A*), as it includes explicit crop-specific distinctions between rainfed and irrigated areas. Minor discrepancies exist in crop growing areas between the two datasets (EarthStat^60^ and MIRCA2000^59,62^). We therefore treated MIRCA2000 as the reference and gap-filled the EarthStat yield data using nearest neighbor interpolation, thus ensuring consistency in spatial coverage of Earthstat-derived yield data with the MIRCA2000 dataset for irrigated and rainfed cropland areas.

Crop-specific growing period calendars representative of the year 2000 were derived from MIRCA2000^59,62^, while crop coefficients and length of crop development stages were derived from Chapagain et al.^20^. The available water content was taken from the Regridded Harmonized World Soil database v1.2^63^.

Global climate and hydrological data for historical and future climate scenarios were sourced from the Inter-Sectoral Impact Model Intercomparison Project, protocol 2b (ISIMIP2b)^33^. We retrieved data for (i) the historical period, which in ISIMIP2b ranges from 1861 to 2005, and (ii) future scenarios (time period: 2005–2100) for three Representative Concentration Pathways (RCPs): RCP 2.6, RCP 6.0, and RCP 8.5, corresponding to different global warming trajectories. In this study, RCPs were selected under fixed 2005 socio-economic conditions (“2005soc” in the data source) so as to isolate and quantify solely the effects of climate change^33^. Despite the availability of the more recent ISIMIP3b dataset, its coverage of hydrological models remains more limited than ISIMIP2b, motivating our decision to use the latter. From the ISIMIP Repository, available at https://data.isimip.org/, we considered data from four GCMs (GFDL-ESM2M, HadGEM2-ES, IPSL-CM5A-LR, and MIROC5) and six GWMs (CWatM, PCR-GLOBWB, LPJmL, H08, WaterGAP2, and MATSIRO) for which the following variables are available: precipitation (called “pr” in the data source), potential evapotranspiration (“potevap”), surface runoff (“qr”), groundwater recharge (“qs”), root-zone soil moisture (“rootmoist”). In ISIMIP2b, precipitation is provided for the four GCMs (GFDL-ESM2M, HadGEM2-ES, IPSL-CM5A-LR, MIROC5) for both the historical period and the three future RCP scenarios, and serves as the atmospheric forcing for the impact models (e.g., GWMs)^33^. The other variables, namely potential evapotranspiration, surface runoff, groundwater recharge, and root-zone soil moisture, are outputs of GWMs (forced by a particular GCM). For example, “potevap” can be generated by the water model CWatM, forced by any of the four climate models (GFDL-ESM2M, HadGEM2-ES, IPSL-CM5A-LR, or MIROC5). In this study, we considered all available combinations of GCMs and GWMs for each historical and RCP scenario. When data are available for every GWM and GCM, then the number of possible combinations is 24 (6 GWMs x 4 GCMs) per variable and scenario. However, this was not always the case. For example, potential evapotranspiration, runoff, and groundwater recharge were not available for two GWMs under RCP 8.5, while root-zone soil moisture had even more limited availability, as summarized in Table 1. The ISIMIP2b variables precipitation and potential evapotranspiration were used to calculate the crop-specific actual evapotranspiration and water footprint, surface runoff and groundwater recharge were combined to determine the blue water renewability rate, while root-zone soil moisture was used to represent the green water renewability rate–as detailed in the following section.

To capture period-specific trends and produce realistic estimates of long-term behaviors, monthly averages of ISIMIP2b variables were computed over different time windows. Specifically, we considered a 3-year window for precipitation and potential evapotranspiration, a five-year window for groundwater recharge and root zone soil moisture, and a ten-year window for surface runoff^38^. Each window was centered around the corresponding year of interest: year 2000 (consistent with the availability of crop-specific data) for the historical period, while future scenarios were evaluated at decadal intervals from 2010 to 2090. Daily variables were derived by assuming uniform distribution of monthly data, except for potential evapotranspiration, which was linearly interpolated between monthly averages^38^. Monthly values were assigned to the midpoint of each month, treating each month as 30 days for simplicity.

### Crop water modeling

Crop water footprint (WF) and water debt (WD) were computed at a cell level with a spatial resolution of 5 × 5 arc-min. We followed the same methodology proposed in previous studies^24,38^ to compute global water use and sustainability indicators for the reference year 2000 (that is the year with the highest availability of spatially distributed data at the time this study was conducted) and for projected future scenarios (every 10 years from 2010 to 2090). We quantified actual evapotranspiration (*ET*) from both irrigated and rainfed crops by adopting a soil water balance modeling framework grounded in the FAO-56 approach^61^. Specifically, for each crop (*cr*), the total evapotranspiration over the length of the growing period (*LGP*) was derived by aggregating daily ET values,

1 $$E{T}_{cr,LGP}={\sum}_{j=1}^{LGP}E{T}_{cr,j},$$

where *ET*_*cr*,*j*_ = *k*_*c*,*cr*,*j*_ ⋅ *k*_*s*,*cr*,*j*_ ⋅ *PET*_*j*_, with *PET*_*j*_ representing the daily reference potential evapotranspiration (derived from GWMs, see Table 1), *j* the day of the growing period, *k*_*c*,*cr*,*j*_ the crop coefficient, and *k*_*s*,*cr*,*j*_ the water stress coefficient. For each crop, *k*_*c*_ varies during the growing season as

2 $${k}_{c,j}=\left\{\begin{array}{ll}{k}_{{{{\rm{c,ini}}}}} \hfill & \,{{{\rm{if}}}}\; j\in {{{\rm{Stage\; I}}}} \hfill \\ j\cdot \frac{{k}_{{{{\rm{c,mid}}}}}-{k}_{{{{\rm{c,ini}}}}}}{j-{l}_{I}} \hfill & \,{{{\rm{if}}}}\;j\in {{{\rm{Stage\; II}}}}\hfill \\ {k}_{{{{\rm{c,mid}}}}} \hfill & \,{{{\rm{if}}}}\;j\in {{{\rm{Stage\; III}}}}\hfill \\ j\cdot \frac{{k}_{{{{\rm{c,end}}}}}-{k}_{{{{\rm{c,mid}}}}}}{j-{l}_{I}-{l}_{II}-{l}_{III}} & \,{{{\rm{if}}}}\;j\in {{{\rm{Stage\; IV}}}}\hfill \end{array}\right.$$

where the length of each stage (*l*_Stage_) was computed as a fraction of *LGP*. Values of the crop coefficients and length of each stage were obtained from the literature^20^.

The water stress coefficient *k*_*s*,*cr*,*j*_ ranges between 0 (maximum water stress) and 1 (no water stress) and was evaluated considering rainfed and irrigated conditions separately. In particular, *k*_*s*,*j*_ = 1 for irrigated conditions (no stress) for the entire duration of the growing period, while for rainfed crops it directly depends on soil moisture (and hence local precipitation derived from GCMs) and was evaluated as ${k}_{s,j}=\frac{TA{W}_{j}-{D}_{mo,j}}{TA{W}_{j}-RA{W}_{j}}$. Here, *TAW* [mm] is the total available water content in the root zone, *RAW* [mm] the readily available water content, and *D*_*mo*_ [mm] is the root zone depletion in the morning, namely the water shortage relative to field capacity. *TAW* depends on the available water content *AWC* [mm/m] and the root depth *Z*_*r*,*j*_ [m] as *TAW*_*j*_ = *AWC* ⋅ *Z*_*r*,*j*_. *AWC* was derived from the literature^63^, while maximum root depths (*Z*_*r*,*max*_) assumed for each crop^61^ are listed in Table S6. The root depth was assumed to linearly increase during the first two growing stages and then maintained constant until harvesting. *RAW* is the water that crops use for evaporation before water stress and stomata closure begins and is given by *RAW*_*j*_ = *ρ* ⋅ *TAW*_*j*_, where *ρ* is the depletion fraction coefficient obtained from the literature^61^ (here assumed constant over the growing season, see Table S6). In rainfed crops, the root zone depletion in the morning, *D*_*mo*,*j*_, is equal to the depletion recorded at the end of the previous day (*D*_*ev*,*j*−1_) minus the daily precipitation value, *D*_*mo*,*j*_ = *D*_*ev*,*j*−1_ − *P*_*j*_. Daily precipitation was obtained by uniformly distributing the monthly rainfall (from GCMs) along the growing season with daily frequency. In the evening, the root zone depletion decreases because of evaporation as *D*_*ev*,*j*_ = *D*_*mo*,*j*_ + *ET*_*cr*,*j*_, with $E{T}_{cr,j}={k}_{c,j}\cdot \frac{TA{W}_{j}-{D}_{mo,j}}{TA{W}_{j}-RA{W}_{j}}\cdot PE{T}_{j}$. Note that losses by deep percolation are neglected and capillary rise is assumed equal to zero. Furthermore, water excess leading to negative *D*_*mo*,*j*_ is cut off at zero (exceeding precipitation is assumed to be lost as surface runoff). In addition, while under rainfed conditions the water volume evapotranspired by the crop during the growing period is only green, under irrigated conditions the daily irrigation volume *I*_*j*_ is determined, assuming that the crop experiences no water stress, as *I*_*j*_ = *D*_*mo*,*j*_ − *RAW*_*j*_ + *k*_*c*,*j*_ ⋅ *PET*_*j*_^38^. In the evening, the root zone depletion is given by *D*_*ev*,*j*_ = *D*_*mo*,*j*_ + *ET*_*cr*,*j*_ − *I*_*j*_. Thus, the blue water corresponds to the irrigation water (*ET*_*b*,*j*_ = *I*_*j*_) while the green water is the difference between the total *ET* and *ET*_*b*,*j*_. No explicit assumptions regarding irrigation practices were introduced in this analysis. Rather, irrigation demand is represented as the exact volume of water required to prevent crop water stress, corresponding to the locally needed amount without over- or under-supply. For rice cultivation, an additional volume per unit area of 200 mm was considered, thus accounting for the water used to prepare the paddy fields^24,38^.

We then derived WF and WD values following established formulations^24,29,38^. Specifically, for each water source *s* (blue or green), crop (*cr*), and location (*l*) the water footprint (WF) was evaluated as

3 $$W{F}_{s,cr,l}=\frac{E{T}_{s,cr,l}}{{Y}_{cr,l}}\cdot P{r}_{cr,l}=VWC_{s,cr,l}\cdot{P{r}_{cr,l}}$$

with VWC being the virtual water content, while the water debt (WD) was computed as

4 $$W{D}_{s,cr,l}=\frac{W{F}_{s,cr,l}}{{A}_{l}\cdot {R}_{s,l}}$$

where *Y* is the annual crop yield (ton ha^−1^), *Pr* the annual crop production (ton), obtained as the product between yield and harvested area, *ET* the crop evapotranspiration during the growing period, *A* the grid cell area (m^2^), and *R* the annual renewability rate of the water source (m yr^−1^). Renewability rates include runoff, groundwater recharge, and root-zone soil moisture, and were derived from different GWMs within ISIMIP2b (see Data section), reflecting variability across models in the internal representation of hydrological processes^41^. To compute WD_b_, we considered a blue water renewability rate (*R*_*b*_) defined as the sum of runoff and groundwater recharge, whereas WD_g_ was calculated using green water renewability (*R*_*g*_) represented by root-zone soil moisture. Further details on aggregation of these metrics across crops and spatial scales are provided in a previous study^38^. To avoid unrealistically low renewability rates, which would lead to artificially high WD estimates, a minimum threshold of 10^−4^ m yr^−1^ was applied to *R* (with all cells below this threshold excluded from the analysis). This value was selected as a compromise between minimizing the number of grid cells excluded from the analysis and limiting the bias in the WD variability estimates associated with the inclusion of excessively low renewability rates (see sensitivity analysis in Supplementary Fig. S21). WF and WD were derived from the same underlying crop water use (evapotranspiration) estimates but differ in their interpretation. WF quantifies the absolute volume of freshwater consumed annually to sustain crop production, whereas WD expresses this consumption relative to the long-term renewability rate of the corresponding water source. Consequently, WD provides a direct measure of sustainability: values ≤1 year indicate that annual crop water use can be replenished within one hydrological cycle, while values  > 1 year indicate that water is being depleted faster than it is renewed (unsustainable), thus requiring more years to be replenished.

### Uncertainty analysis

We adopted the standard deviation as the primary uncertainty metric, as it provides a robust (Supplementary Fig. S19) and balanced characterization of the ensemble spread. Alternative measures, including the interquartile range (IR) and the maximum-minimum range, were also evaluated (Supplementary Fig. S22). While the IR reduces the influence of extreme values and thus yields a slightly more conservative estimate of variability^50^, the maximum-minimum range is highly sensitive to individual outlier model estimates. In comparison, the standard deviation retains sensitivity to variability across all models without being disproportionately influenced by single extreme realizations, thereby offering a suitable compromise for representing uncertainty in this study.

To quantify the magnitude of uncertainty, we adopted the coefficient of variation (*CV*) defined as the standard deviation (*σ*) across all model estimates normalized by the multi-model ensemble mean:

5 $$CV=\frac{\sigma (V)}{\overline{V}},$$

where *V* denotes the considered variable (here, WF or WD) in each grid cell.

To determine the dominant source of uncertainty (Fig. 4a-f) and explicitly account for potential interactions between GWMs and GCMs, we applied a two-factor analysis of variance (ANOVA) framework^64^. Let *V*_*i*,*j*_(*g*) denote the simulated variable (WF or WD) at the grid cell *g*, produced by the *i*-th GWM and *j*-th GCM. The grand mean, namely the average across all models, is defined as ${\overline{V}}_{\cdot \cdot }(g)=\frac{1}{24}{\sum }_{i=1}^{6}{\sum }_{j=1}^{4}{V}_{i,j}(g)$. Furthermore, the average across all GCMs for each GWM is defined as ${\overline{V}}_{i\cdot }(g)=\frac{1}{4}{\sum }_{j=1}^{4}{V}_{i,j}(g)$, reflecting the spread among different water models (GWM contribution), while the average across all GWMs for each GCM is ${\overline{V}}_{\cdot j}(g)=\frac{1}{6}{\sum }_{i=1}^{6}{V}_{i,j}(g)$, indicating the spread among climate models (GCM contribution). At each pair *i*, *j*, the residuals representing the GCM-GWM interactions are defined as ${r}_{i,j}(g)={V}_{i,j}(g)-{\overline{V}}_{i\cdot }(g)-{\overline{V}}_{\cdot j}(g)+{\overline{V}}_{\cdot \cdot }(g)$. If the residuals are equal to zero for all *i*, *j*, there are no interactions among GWMs and GCMs. The total variance can then be decomposed as:

6 $${\sigma }_{\,{{{\rm{tot}}}}}^{2}={\sigma }_{W}^{2}+{\sigma }_{C}^{2}+{\sigma }_{I}^{2},$$

where ${\sigma }_{tot}^{2}={{{\rm{Var}}}}[{V}_{ij}(g)]$, ${\sigma }_{W}^{2}={{{\rm{Var}}}}[{\overline{V}}_{i\cdot }(g)]$ represents the GWM contribution, ${\sigma }_{C}^{2}={{{\rm{Var}}}}[{\overline{V}}_{\cdot j}(g)]$ the GCM contribution, and ${\sigma }_{I}^{2}={{{\rm{Var}}}}[{r}_{i,j}(g)]$ the interaction term. This formulation allowed a statistically consistent partitioning of uncertainty without assuming independence between sources^64^. The relative importance of each uncertainty source was assessed by comparing the fractional contributions of the variance components on the right-hand side of Eq. (6). Specifically, the dominant source of uncertainty was identified as the one with the highest value among ${\sigma }_{W}^{2}/{\sigma }_{tot}^{2}$, ${\sigma }_{C}^{2}/{\sigma }_{tot}^{2}$, and ${\sigma }_{I}^{2}/{\sigma }_{tot}^{2}$. The most influential model (as shown in panels g-i in Fig. 4) was then identified by sequentially excluding one model from the ensemble and recomputing the resulting standard deviation. The model whose exclusion led to the largest difference in the ensemble uncertainty was classified as the major contributor to the total uncertainty.

Lastly, to assess the scale-dependency of uncertainty (Supplementary Fig. S15) we computed the total coefficient of variation *CV* across spatial scales. The analysis was performed by progressively disaggregating values from the global mean down to the single grid cell, effectively convolving model outputs across increasing levels of spatial detail, through a moving window with dimensions equal to the scale considered.

## Supplementary information

Transparent Peer Review file

Supplementary Information

## Data Availability

Data results for each crop, model, year, and scenario are available via drop4crop web-mapping platform at https://drop4crop.epfl.ch/ with CCA 4.0 licence.

## Code availability

The figures were generated using MATLAB and m_map (https://www.eoas.ubc.ca/~rich/map.html), where the basemap data (GSHHG, https://www.ngdc.noaa.gov/mgg/shorelines/gshhs.html) are used under the GNU Lesser General Public license. All the codes used in the data processing and visualization are available via Zenodo at 10.5281/zenodo.19235604.
