# Supplementary material for: Uncertainties in global hydrological and climate models challenge future estimates of crop water use and sustainability
Source: Commun Earth Environ. 2026 May 26;7(1):676. doi: 10.1038/s43247-026-03621-w (PMC13485690; doi:10.1038/s43247-026-03621-w)
Supplement: Supplementary file 1 [file 43247_2026_3621_MOESM1_ESM.pdf]

nature portfolio

Peer Review File

Uncertainties in global hydrological and climate models challenge future estimates of crop water use and sustainability

Corresponding Author: Professor Sara Bonetti

This file contains all editorial decision letters in order by version, followed by all author rebuttals in order by version.

Version 0:

Decision Letter:

** Please ensure you delete the link to your author home page in this e-mail if you wish to forward it to your coauthors **

Dear Professor Bonetti,

Your manuscript titled "Uncertainties in global hydrological and climate models challenge future estimates of crop water use and sustainability" has now been seen by 2 reviewers, and we include their comments at the end of this message. They find your work of interest, but some important points are raised. We are interested in the possibility of publishing your study in Communications Earth & Environment, but would like to consider your responses to these concerns and assess a revised manuscript before we make a final decision on publication.

In particular, please ensure that the revised manuscript meets the following editorial thresholds:
1. Present a compelling quantification of how global climate- and hydrology-model uncertainty propagates into estimates of crop water needs and sustainability.
2. Ensure that you fully describe and justify your methods and assumptions, including the definition and quantification of uncertainty, the use of the max–min range as the primary uncertainty metric, the renewability-rate calculations, and the ks parameter calculation.

We therefore invite you to revise and resubmit your manuscript, along with a point-by-point response that takes into account the points raised. Please highlight all changes in the manuscript text file.

Please submit your point-by-point responses as a separate file, distinct from your cover letter where you can add responses to the Editors' comments that you do not want to be made available to the reviewers. Word files are preferred. We recommend that any figures, tables or graphs that are included in the response to reviewers are also included in the main article or Supplementary Information.

We are committed to providing a fair and constructive peer-review process. Please don't hesitate to contact us if you wish to discuss the revision in more detail.

Please use the following link to submit your revised manuscript, point-by-point response to the referees' comments (which should be in a separate document to any cover letter), a tracked-changes version of the manuscript (as a PDF file) and the completed checklist:
Link Redacted
** This url links to your confidential home page and associated information about manuscripts you may have submitted or be reviewing for us. If you wish to forward this email to co-authors, please delete the link to your homepage first **

We hope to receive your revised paper within six weeks; please let us know if you aren't able to submit it within this time so that we can discuss how best to proceed. If we don't hear from you, and the revision process takes significantly longer, we may close your file. In this event, we will still be happy to reconsider your paper at a later date, as long as nothing similar has been accepted for publication at Communications Earth & Environment or published elsewhere in the meantime.

Please do not hesitate to contact us if you have any questions or would like to discuss these revisions further. We look

forward to seeing the revised manuscript and thank you for the opportunity to review your work.

Best regards,

Nicola Colombo, PhD
Associate Editor, Communications Earth & Environment
Consulting Editor, Communications Sustainability

EDITORIAL POLICIES AND FORMATTING

We ask that you ensure your manuscript complies with our editorial policies. Please ensure that the following formatting requirements are met, and any checklist relevant to your research is completed and uploaded as a Related Manuscript file type with the revised article.

For Manuscripts that fall into the following fields:
• Behavioural and social science
• Ecological, evolutionary & environmental sciences
• Life sciences
An updated and completed version of our Reporting Summary must be uploaded with the revised manuscript
You can download the form here:
https://www.nature.com/documents/nr-reporting-summary.pdf

Furthermore, please align your manuscript with our format requirements, which are summarized on the following checklist:
Communications Earth & Environment formatting checklist

and also in our style and formatting guide Communications Earth & Environment formatting guide .

*** DATA: Communications Earth & Environment endorses the principles of the Enabling FAIR data project (http://www.copdess.org/enabling-fair-data-project/). We ask authors to make the data that support their conclusions available in permanent, publically accessible data repositories. (Please contact the editor if you are unable to make your data available).

All Communications Earth & Environment manuscripts must include a section titled "Data Availability" at the end of the Methods section or main text (if no Methods). More information on this policy, is available at http://www.nature.com/authors/policies/data/data-availability-statements-data-citations.pdf.

In particular, the Data availability statement should include:
- Unique identifiers (such as DOIs and hyperlinks for datasets in public repositories)
- Accession codes where appropriate
- If applicable, a statement regarding data available with restrictions
- If a dataset has a Digital Object Identifier (DOI) as its unique identifier, we strongly encourage including this in the Reference list and citing the dataset in the Data Availability Statement.

DATA SOURCES: All new data associated with the paper should be placed in a persistent repository where they can be freely and enduringly accessed. We recommend submitting the data to discipline-specific, community-recognized repositories, where possible and a list of recommended repositories is provided at http://www.nature.com/sdata/policies/repositories.

If a community resource is unavailable, data can be submitted to generalist repositories such as figshare or Dryad Digital Repository. Please provide a unique identifier for the data (for example a DOI or a permanent URL) in the data availability statement, if possible. If the repository does not provide identifiers, we encourage authors to supply the search terms that will return the data. For data that have been obtained from publically available sources, please provide a URL and the specific data product name in the data availability statement. Data with a DOI should be further cited in the methods reference section.

Please refer to our data policies at http://www.nature.com/authors/policies/availability.html.

REVIEWER COMMENTS:

Reviewer #1 (Remarks to the Author):

This manuscript addresses how climate- and hydrology-model uncertainty propagates into global crop water footprint and

water debt estimates at high spatial resolution. The work is relevant for water sustainability assessments, and the use of ISIMIP2b multi-model ensembles is appropriate. The manuscript is generally well structured and clearly written; the figures illustrate the spatial patterns. However, several methodological assumptions underlying the uncertainty analysis require clearer justification and additional discussion. The treatment of uncertainty risks overstating the robustness of some conclusions, particularly regarding the relative roles of GCMs and GWMs. I therefore recommend major revision.

1. The total uncertainty is defined as the sum of GWM uncertainty (W) and GCM uncertainty (C). This linear additivity is not formally justified. W and C are not statistically independent quantities, and potential GCM × GWM interaction effects are ignored. As a result, the proposed total uncertainty may not represent the true envelope of the ensemble, nor a variance-equivalent measure. A sensitivity test or comparison with an alternative metric would further strengthen the analysis.

2. The max–min range is used as the primary uncertainty metric. While the approach is simple and intuitive, it is highly sensitive to individual outlier models, especially given the small model ensemble. The manuscript should assess whether the reported uncertainty patterns are robust. It is important as several conclusions rely on relative magnitudes of uncertainty across crops, regions, and scenarios.

3. The number of GWMs differs across RCPs, and key variables for WDg are missing in some scenarios. This limits comparability of uncertainty magnitudes across scenarios and water components. Please make this limitation explicit.

4. WD is a ratio involving renewable water availability. Near-zero availability will inflate WD and can dominate max–min uncertainty. Please specify how low or zero renewable water is handled (masking, lower bounds, or other QC), and quantify whether extreme grid cells drive reported spreads and hotspots. A simple sensitivity analysis for the WD threshold would strengthen the hotspot interpretation.

Minor:
1. Crop differentiation relies on fixed crop coefficients and static cropping calendars. This is acceptable for global assessment. However, the implications of ignoring adaptive phenology and management should be more explicitly stated.

Reviewer #2 (Remarks to the Author):

This study assesses inter-model uncertainty in projected crop water demands across an ensemble of global climate and water models. Results show generally larger inter-model variability for blue water, which is dominantly driven by GWMs, and smaller inter-model variability for green water, which is dominantly driven by GCMs. The article is a useful contribution to this literature on the sustainability of future crop water requirements, in which inter-model uncertainty is generally large and under-characterized. The approach is overall sound, and the results shown generally support the results.
That said, there are several reasonably important methodological and clarity problems that need to be addressed before this article can be published. The main issues are that the definition and quantification of 'uncertainty' is vague in the intro and methods, and the measures used are technically incorrect and not sufficiently firmly grounded in formal statistics. For several key steps in the analysis (notably the renewability rate calculations), the rationale and justification for the methods is absent and we get only references to other papers. I looked at some of the papers and did not find the necessary explanations. These steps need to be clarified for me to assess whether the results are sound and meaningful. All these concerns are enumerated in the line comments below, and I think they should all be addressable within the paper's existing framework.

Intro – layout why this uncertainty matters.
L42 individual crop responsibility – not sure I understand the term. I think you mean attribution of aggregate water requirements for an area to different crops that might be grown there, which is obviously policy relevant information?
L43-46: Given the centrality of WD and WF to the analysis, expanding a bit on the meaning of these two metrics could ease interpretation of the whole paper. I did not find further explanation in the Methods. How are they different/similar? Does the distinction between mass (volume) vs time basis give different impressions of water requirements? A couple extra sentences on this would be enough. It could also be explained further why these two metrics are more physical (you compare to threshold-based definitions, but to me thresholds can easily be physically based and crop-specific).
L78-80: I wouldn't call this output a byproduct – its inherent to the problem you are trying to solve. Without characterizing model ensemble uncertainty, we have no idea how likely the water sustainability projections of a given model are to actually arise. The likelihood is a key piece of information, beyond the projections of single models, that is essential to characterizing what the future is likely to look like.
L100: Is this lack of trend for the ensemble mean or individual models? I think it is for the ensemble mean, which is perhaps not the most meaningful context-setting result in a study focused on inter-model variability. Is there a subset of individual models that have significant trends under lower emissions scenarios? And vice versa?
L110: Consider clarifying what you mean by 'robust' for fractional uncertainty < 1
L112-119: There are two things to explain here. 1) Why is model spread for WDb so much larger than signal compared to WF? 2) Regions with F>1 for WF align closely with hotspots of high F for WDb. Why those regions? You refer to the SI figures at the end of this paragraph, but the interpretation is not clear. Those figures show variability between crops – are differences between crops driving these two things? A bit of mechanistic expansion might be illuminating.
L120-126: Ah ok here is more explanation of why these regions. It may improve organization to group expansion on point 1 above into a single paragraph, then group regional hotspot discussion (point 2) into a separate paragraph. Glancing between the panels, I think I mostly agree with the spatial correspondence between F for WDb and the magnitude of WDb itself. A more rigorous approach could be to plot WDb vs F(WDb) – the corresponding regression statistics would give a clear sense of how strong this spatial dependence is (maybe in the SI as this figure already has a lot of data).

L126: So is the interpretation here that model differences have a larger impact on total uncertainty in regions where both 1) total crop water demands are high and 2) crop water demands are highly concentrated in a small number of crops? Why is that? An extra sentence on interpretation would help.
L134: The paper below on CO_2-vegetation effects on runoff has a similar argument regarding the influence of spatial averaging on uncertainty estimation. It could be a useful reference:
https://www.nature.com/articles/s44221-024-00361-z

L139-141: A stylistic point but I think this transition is very neutral misses an opportunity to express why it is important to figure out the origins of uncertainty (i.e. draw a clearer link with the preceding section).
L146-147: There are only 4 GCMs used in the study, compared to 6 GWMs. Might you expect having 50% more GWMs to give you a greater range simply because of the sample difference? It's also worth noting that any 2 GCMs run with the same forcing can give different answers for 2 reasons: differences in model assumptions or process representation, and chaotic internal variability. Here they are lumped together. The way to solve that is by isolating internal variability using large ensembles from a single model. That might not be possible given limited models participating in ISIMIP, but this problem should be noted and interpreted.
Fig. 3: I find it hard to compare values in this figure. My eye is drawn to the size of the dots and it's hard to simultaneously compare the colors, which is the data we care about. Consider rethinking the visualization here so that the key message (WF range, not continental gross production) is more visually dominant.
Regarding CO_2 physiological effects: Is the effect of CO_2 on stomatal conductance, crop water demand, and renewability rates included in the GWMs? It should be by default in the GCMs, and ideally this should be consistent across the modeling chain. Perhaps different physiology representations in GWMs is part of why the range of projected PET is so large?
Methods: It is hard to figure out at many points in the methods which data are being used and for what. It is common to group the description of all primary data inputs as a section called "data" at the very beginning of the methods. Generally the methods require clarification throughout.
L251-253: Much more detail is required on how ks is calculated from the precipitation inputs from GCMs. This parameter is a key pivot point between GCM and GWM inputs, so it's essential to explain how it is calculated. I did not find the necessary details in the cited articles. Even if that information were clearly presented there, it should be summarized here in sufficient detail that readers can evaluate whether it is reasonable and what assumptions are embedded. It's also worth noting that the GCMs (and presumably the GWMs) also simulate soil moisture, so worth clarifying why you don't use those data directly.
L261: I think the renewability rates ultimately come from WaterGAP – summarize the data and justify why they are appropriate as applied here. This poses a bit of a problem here, because R comes from one of the models in the ensemble being evaluated, and is being applied to all of them. Presumably each model has its own representation of the relevant surface, soil and groundwater processes. So, using R from a single model probably underestimates the underlying model spread.
L270: I agree these values for popcorn make no sense – probably worth tracing the error and alerting the dataset manager, as it may signal quality control issues. The use of the ÷ symbol here is nonstandard, consider – or ~ or the word 'to'
L308-318: It generally is not very meaningful to sum ranges of 2 variables as an estimate of multivariate uncertainty. First, it ignores dependence structure between the two variables, and ignores all other data points than the max and min. Second, the range is not firmly grounded in statistical/probability theory, whereas variance is (and you are hinting at probabilistic projections in this paper). Third, summing ranges is inconsistent with error propagation theory. The standard approach would be to use variance and consider the total variance as σtotal2=σx2+σy2+2Cov(x,y). If you have a good reason not to use this more formally correct approach, it should be stated.
Code availability: Statement says processing codes are on zenodo, but I only see figure reproduction code included. Full code required to reproduce the results should be included. See guidelines in:
https://www.pnas.org/doi/10.1073/pnas.2422157123

** Visit Nature Portfolio's author and referees' website at www.nature.com/authors for information about policies, services and author benefits**

Communications Earth & Environment is committed to improving transparency in authorship. As part of our efforts in this direction, we are now requesting that all authors identified as 'corresponding author' create and link their Open Researcher and Contributor Identifier (ORCID) with their account on the Manuscript Tracking System prior to acceptance. ORCID helps the scientific community achieve unambiguous attribution of all scholarly contributions. You can create and link your ORCID from the home page of the Manuscript Tracking System by clicking on 'Modify my Springer Nature account' and following the instructions in the link below. Please also inform all co-authors that they can add their ORCIDs to their accounts and that they must do so prior to acceptance.
https://www.springernature.com/gp/researchers/orcid/orcid-for-nature-research

For more information please visit http://www.springernature.com/orcid

If you experience problems in linking your ORCID, please contact the Platform Support Helpdesk.

Version 1:

Decision Letter:

** Please ensure you delete the link to your author home page in this e-mail if you wish to forward it to your coauthors **

Dear Professor Bonetti,

Your manuscript titled "Uncertainties in global hydrological and climate models challenge future estimates of crop water use and sustainability" has now been seen by our reviewers, whose comments appear below. In light of their advice we are delighted to say that we are happy, in principle, to publish a suitably revised version in Communications Earth & Environment.

We therefore invite you to revise your paper one last time to address the remaining concerns of our reviewers. At the same time we ask that you edit your manuscript to comply with our format requirements and to maximise the accessibility and therefore the impact of your work.

EDITORIAL REQUESTS:

Please review our specific editorial comments and requests regarding your manuscript in the attached "Editorial Requests Table".

*****Please take care to match our formatting and policy requirements. We will check revised manuscript and return manuscripts that do not comply. Such requests will lead to delays. *****

Please outline your response to each request in the right hand column. Please upload the completed table with your manuscript files as a Related Manuscript file.

If you have any questions or concerns about any of our requests, please do not hesitate to contact me.

SUBMISSION INFORMATION:

In order to accept your paper, we require the files listed at the end of the Editorial Requests Table; the list of required files is also available at https://www.nature.com/documents/commsj-file-checklist.pdf .

OPEN ACCESS:

Communications Earth & Environment is a fully open access journal. Articles are made freely accessible on publication. For further information about article processing charges, open access funding, and advice and support from Nature Portfolio, please visit https://www.nature.com/commsenv/open-access

At acceptance, you will be provided with instructions for completing the open access licence agreement on behalf of all authors. This grants us the necessary permissions to publish your paper. Additionally, you will be asked to declare that all required third party permissions have been obtained, and to provide billing information in order to pay the article-processing charge (APC).

Please use the following link to submit the above items:
Link Redacted
** This url links to your confidential home page and associated information about manuscripts you may have submitted or be reviewing for us. If you wish to forward this email to co-authors, please delete the link to your homepage first **

We hope to hear from you within two weeks; please let us know if you need more time.

Best regards,

Yangxiaoyue Liu, PhD
Editorial Board Member
Communications Earth & Environment
orcid.org/0000-0003-3762-117X

Nicola Colombo, PhD
Associate Editor, Communications Earth & Environment
Consulting Editor, Communications Sustainability

REVIEWERS' COMMENTS:

Reviewer #1 (Remarks to the Author):

The authors have done a good job addressing the reviewers' concerns. The manuscript is now robust and provides important insights into the propagation of model uncertainties in global crop water assessments.

The authors might consider adding a brief concluding sentence that explicitly links the high inter-model uncertainty in these areas to specific local water management challenges, thereby further enhancing the policy relevance of this work.

Reviewer #2 (Remarks to the Author):

Thanks to authors for the thorough revisions. The new uncertainty framework is more solid, yet the conclusions are consistent with the original framework. I am also reassured that the uneven ensemble of GCMs and GWMs probably doesn't influence the results that much. There are many remaining limitations that cannot be addressed within the structure of the ISIMIP protocol employed here – these are issues to large to solve here, and I think the authors now do a sufficient job describing these limitations. Only a couple small adjustments to recommend, for clarity:
Fig. S9 – Including only the R2 for bin means inflates the strength of the dependence – I'd favour the lower R2 for the underlying data as the annotation on the panel, or you can include both.
L480 – I think you're referring to Fig. S18, not S17.

** Visit Nature Portfolio's author and referees' website at www.nature.com/authors for information about policies, services and author benefits**

Open Access This Peer Review File is licensed under a Creative Commons Attribution 4.0 International License, which permits use, sharing, adaptation, distribution and reproduction in any medium or format, as long as you give appropriate credit to the original author(s) and the source, provide a link to the Creative Commons license, and indicate if changes were made.
In cases where reviewers are anonymous, credit should be given to 'Anonymous Referee' and the source.
The images or other third party material in this Peer Review File are included in the article's Creative Commons license, unless indicated otherwise in a credit line to the material. If material is not included in the article's Creative Commons license and your intended use is not permitted by statutory regulation or exceeds the permitted use, you will need to obtain permission directly from the copyright holder.
To view a copy of this license, visit https://creativecommons.org/licenses/by/4.0/

<u>REVISION NOTES</u> - Manuscript *"Uncertainties in global hydrological and climate models challenge future estimates of crop water use and sustainability"* (manuscript number COMMSENV-25-6197-T) by Sun et al.

<u>Reviewer #1</u>

This manuscript addresses how climate- and hydrology-model uncertainty propagates into global crop water footprint and water debt estimates at high spatial resolution. The work is relevant for water sustainability assessments, and the use of ISIMIP2b multi-model ensembles is appropriate. The manuscript is generally well structured and clearly written; the figures illustrate the spatial patterns. However, several methodological assumptions underlying the uncertainty analysis require clearer justification and additional discussion. The treatment of uncertainty risks overstating the robustness of some conclusions, particularly regarding the relative roles of GCMs and GWMs. I therefore recommend major revision.

> <u>Reply</u>: We thank the Reviewer for their appreciation of our manuscript and the insightful comments. We have found all the suggestions highly valuable to improve and strengthen our study. In the revised manuscript, we addressed all points raised, as detailed in our comments below. Specifically, we have (i) updated our analyses by adopting more statistically justified metrics for the quantification of uncertainty, namely the two-factor analysis of variance for interactions across models, and the standard deviation instead of the max-min range, (ii) revised the results and discussion accordingly, (iii) broadened the discussion around the number of models adopted and the limitations of our framework, and (iv) included a sensitivity analysis for the selected minimum thresholds of renewability rates. In the revised manuscript, the changes are visible thanks to the track-change mode. Whenever possible, we also copied here extracts of modified text below the corresponding reply (highlighted in blue the new text additions, strikethrough text in red for deleted text). Line numbers reported for these excerpts refer to the version displayed in track-changes mode.

1. The total uncertainty is defined as the sum of GWM uncertainty (W) and GCM uncertainty (C). This linear additivity is not formally justified. W and C are not statistically independent quantities, and potential GCM × GWM interaction effects are ignored. As a result, the proposed total uncertainty may not represent the true envelope of the ensemble, nor a variance-equivalent measure. A sensitivity test or comparison with an alternative metric would further strengthen the analysis.

> <u>Reply</u>: We thank the Reviewer for raising such a relevant point. We agree that the original formulation of total uncertainty as the sum of GWM and GCM contributions implicitly assumed independence between the two sources and neglected potential interaction effects. As correctly pointed out by both Reviewers (see also point n. 19, Reviewer #2), this assumption is not formally justified and may lead to a misrepresentation of the total uncertainty. To address this concern, we have revised the uncertainty framework by explicitly accounting for GCMxGWM interactions using a two-factor ANOVA approach (Montgomery, 2017). This allows a formal variance decomposition into three components: (i) GWM-related uncertainty, (ii) GCM-related uncertainty, and (iii) their interaction term. The revised formulation no longer relies on linear additivity but instead provides a statistically consistent partitioning of the total variance.

In addition, we replaced the previously defined fractional uncertainty metric (F=(W+C)/mean(V)) with the coefficient of variation computed across all model combinations, defined as CV= std(V)/mean(V), where V is the variable of interest (water footprint or water debt). This metric directly reflects the total ensemble spread without any assumptions on independence, and is coherent with the substitution of max-min range with the standard deviation (see point 2 below). We have therefore revised the entire manuscript accordingly. In particular:

- Figure 2 (**Fig. R1** here) and Figure A3 (scale-dependency) have been updated using the new coefficient of variation (CV). Importantly, the main hotspots of uncertainty emerging from the previous fractional uncertainty remained overall consistent, thereby supporting the same conclusions as in the original manuscript;
- The Results (lines 127-162, 171-210) and Methods (lines 477-517) sections have been revised to reflect these updated analyses;
- Figure 4 (**Fig. R2** here) has been extended to include the interaction term (σ^2_I), alongside the GCM (σ^2_C) and GWM (σ^2_W) contributions, providing a complete variance decomposition (thoroughly presented in the revised Methods – lines 489-507).

Although the key findings presented in the original version of the manuscript are largely unaffected by the initial simplifying assumptions, particularly regarding the location of hotspots of high uncertainty and/or high unsustainability, we acknowledge that these revisions have substantially strengthened the methodological rigor of our analyses.

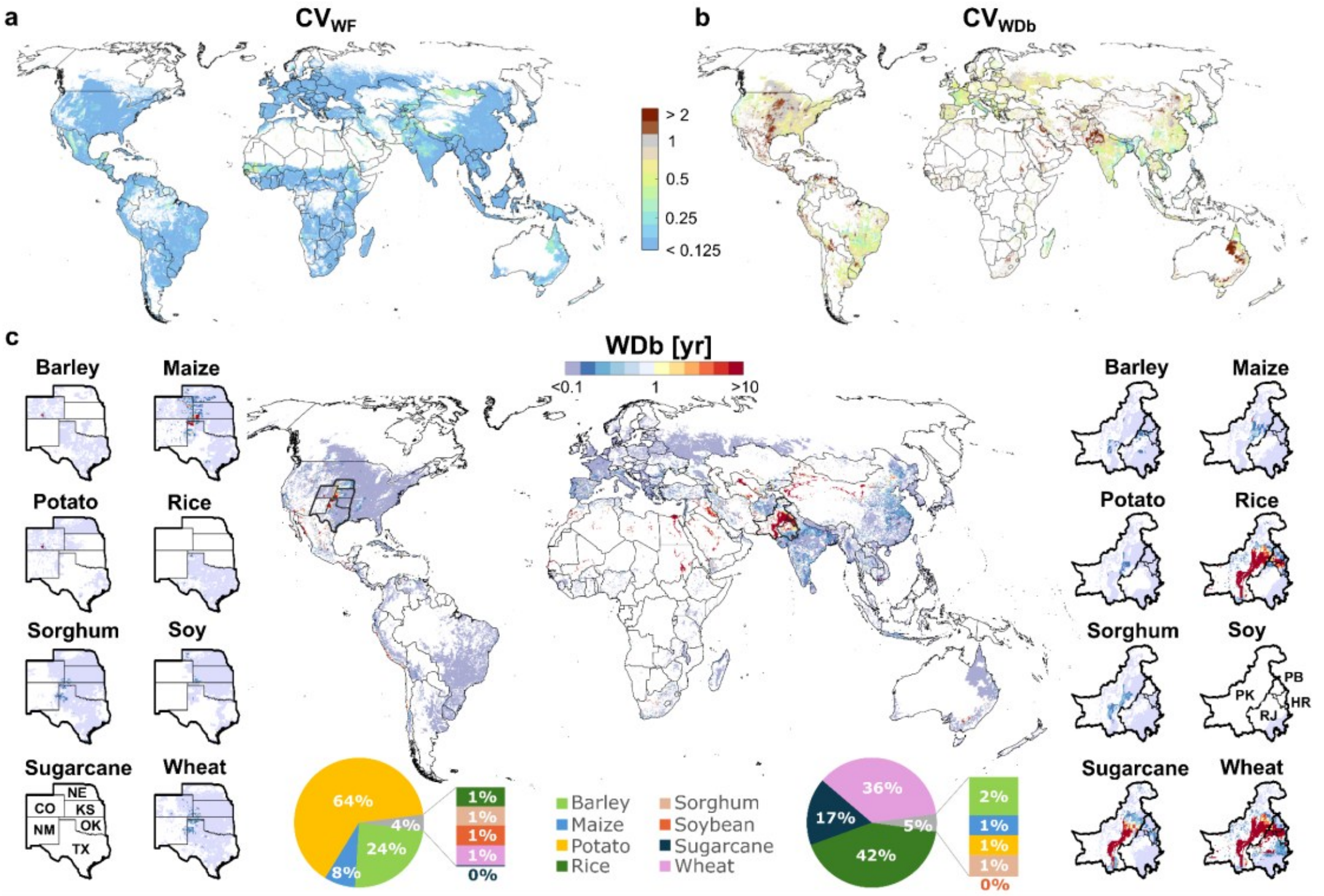

Figure R1 (updated Fig. 2 in the revised manuscript): End-of-century spatial distribution of coefficient of variation and blue water debt. Coefficient of variation CV of total water footprint (WF=WFb+WFg) (**a**) and blue water debt WDb (**b**) for the year 2090 (RCP 8.5). (**c**) Blue water debt (WDb) accounting for all crops and averaged across all climate and water models (year 2090, RCP 8.5). Solid black boundaries highlight two hotspot regions (WDb>10 years) for which crop-specific WDb values are shown on the left side (Nebraska (NE), Kansas (KS), Oklahoma (OK), Texas (TX), New Mexico (NM), Colorado (CO), United States) and right side (Pakistan (PK); Indian provinces of Punjab (PB), Haryana (HR), and Rajasthan (RJ)). Pie charts show percentage contributions of each crop to WDb. High percentages of potato and barley in the USA derive from local WDb>>10 in Colorado.

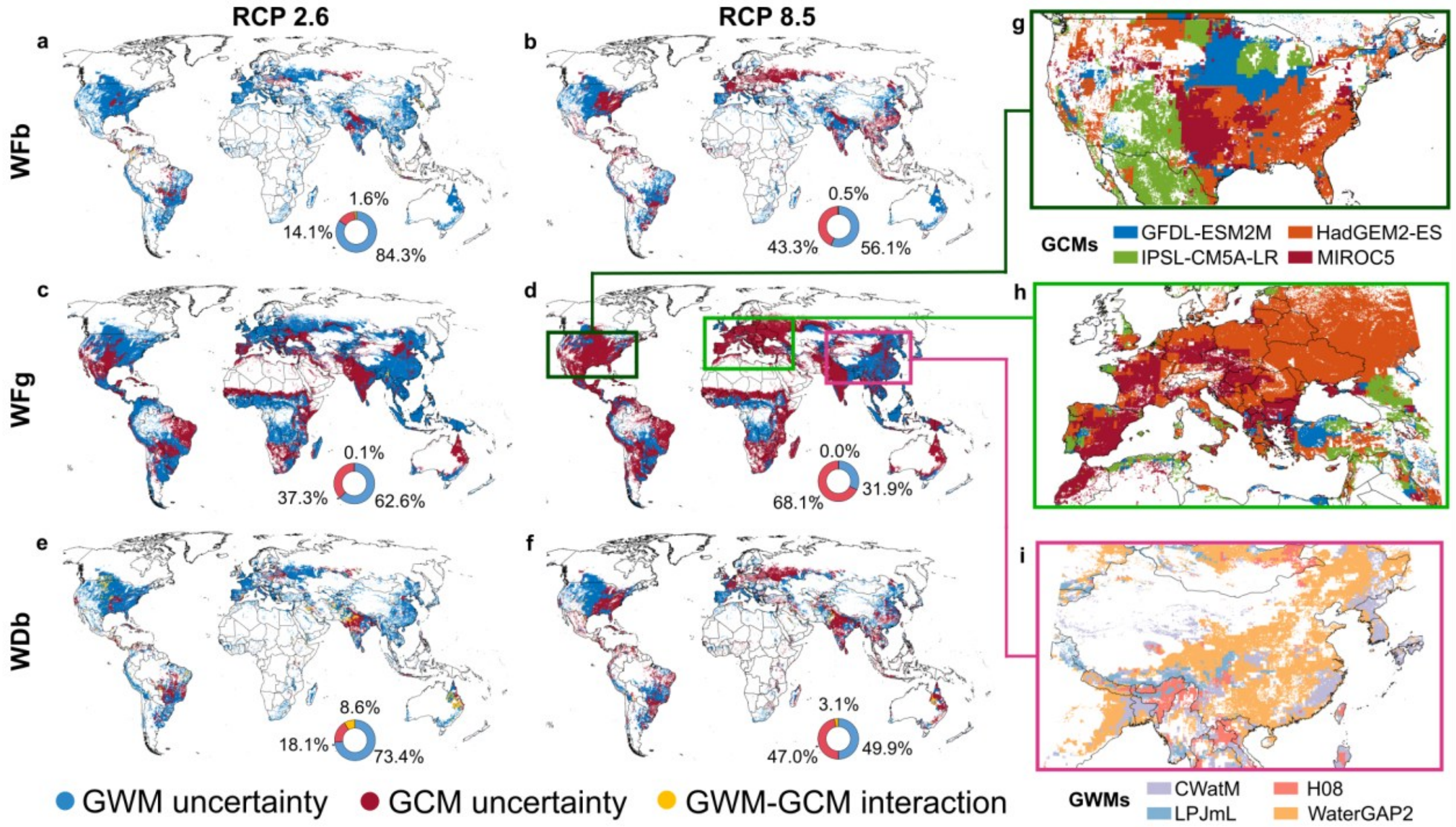

Figure R2 (updated Fig. 4 in the revised manuscript): Dominant sources of uncertainty. Global scale dominant source of uncertainty deriving from water models (GWM, in blue), climate models (GCM, in red), and GWM-GCM interactions (in yellow), for blue water footprint WFb (**a**,**b**), green water footprint WFg (**c**,**d**), and blue water debt WDb (**e**,**f**). The global maps refer to the year 2090, under the RCP 2.6 (first column) and RCP 8.5 (second column). Doughnut charts show the global area proportion of each source (Methods). The three insets associated with the RCP 8.5 projection of WFg feature macro-areas for which the dominant source of uncertainty is further examined by highlighting the corresponding GCMs (**g**,**h**) and GWMs (**i**). Results are based on four climate models for all RCPs, combined with six GWM models for RCP 2.6 and four GWM models for RCP 8.5. The complete list of GCMs and GWMs is provided in the Methods.

2. The max–min range is used as the primary uncertainty metric. While the approach is simple and intuitive, it is highly sensitive to individual outlier models, especially given the small model ensemble. The manuscript should assess whether the reported uncertainty patterns are robust. It is important as several conclusions rely on relative magnitudes of uncertainty across crops, regions, and scenarios.

> <u>**Reply**</u>: We thank the Reviewer for this comment and fully agree that the use of the max-min range may be sensitive to outliers, particularly in relatively small model ensembles. We have thus extended our analysis by considering two additional, widely used, statistical measures of variability: the standard deviation and the interquartile range (IR, i.e., the difference between the 75th and 25th percentiles), as done, e.g., in Prudhomme et al. (2014). A comparative assessment of these metrics (performed for the results shown in **Fig. R3** below) indicates that IR tends to dampen the contribution of extreme model outcomes, leading to a more conservative estimate of uncertainty. In contrast, the standard deviation provides an intermediate characterization of ensemble spread, retaining sensitivity to variability across all models without being disproportionately driven by single outliers, as for the case of the max-min metric (see **Figs. R3-R4** in this document).

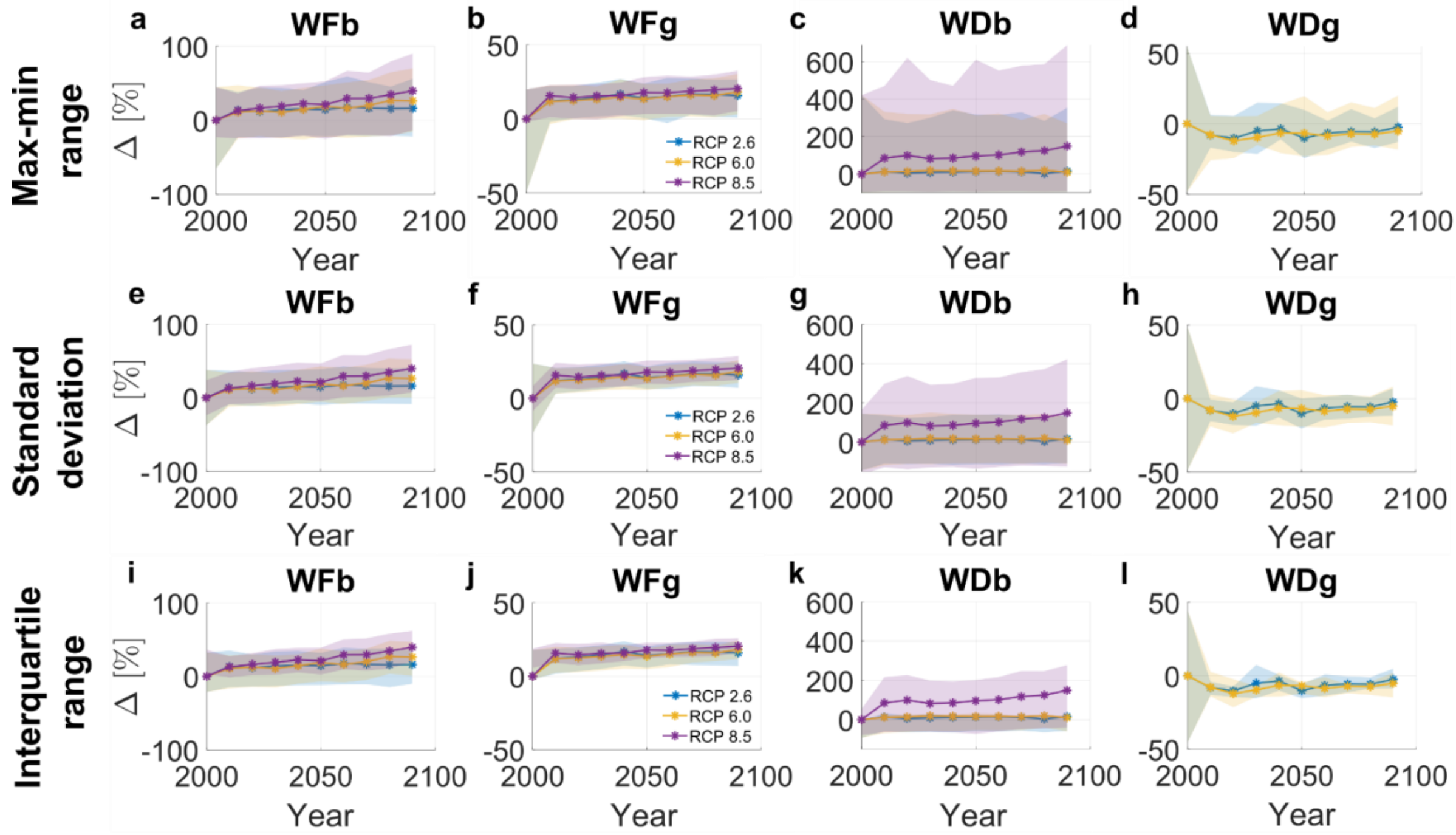

Figure R3 (new supplementary Fig. S18 in the revised manuscript): Comparative assessment of the variability in projections of global water footprint and water debt estimates using different metrics. Temporal evolution of the percentage change (Δ) (relative to the average in the reference year 2000) of global blue water footprint (WFb), green water footprint (WFg), blue water debt (WDb), and green water debt (WDg) - accounting for all the 8 crops. Solid lines represent average values across all GWMs and GCMs under RCP 2.6 (blue), 6.0 (yellow), and 8.5 (purple). The uncertainty ranges denoting variability among values from all models (colored shaded areas) are computed as: the maximum-minimum difference (panels **a-d**, equal to the original version of Fig. 1), the standard deviation (panels **e-h**, equal to the new Fig. 1 in the revised manuscript), and the interquartile range (panels **i-l**).

Based on this – and for coherence with the decomposition of variance (fully addressed in point 1 above) – we have decided to adopt the standard deviation as the primary uncertainty metric in the revised manuscript, while including the differences among the three metrics in the Supplementary Information (new supplementary Fig. S17). In particular, we have revised the Methods as detailed in the following (lines 478-485):

"Methods

[...]

Uncertainty analysis

We adopted the standard deviation as the primary uncertainty metric, as it provides a robust (Fig. S17) and balanced characterization of the ensemble spread. Alternative measures, including the interquartile range (IR) and the maximum-minimum range, were also evaluated (Fig. S18). While the IR reduces the influence of extreme values and thus yields a slightly more conservative estimate of variability (Prudhomme et al., 2014), the maximum-minimum range is highly sensitive to individual outlier model estimates. In comparison, the standard deviation retains sensitivity to variability across all models without being disproportionately influenced by single extreme realizations, thereby offering a suitable compromise for representing uncertainty in this study. [...]."

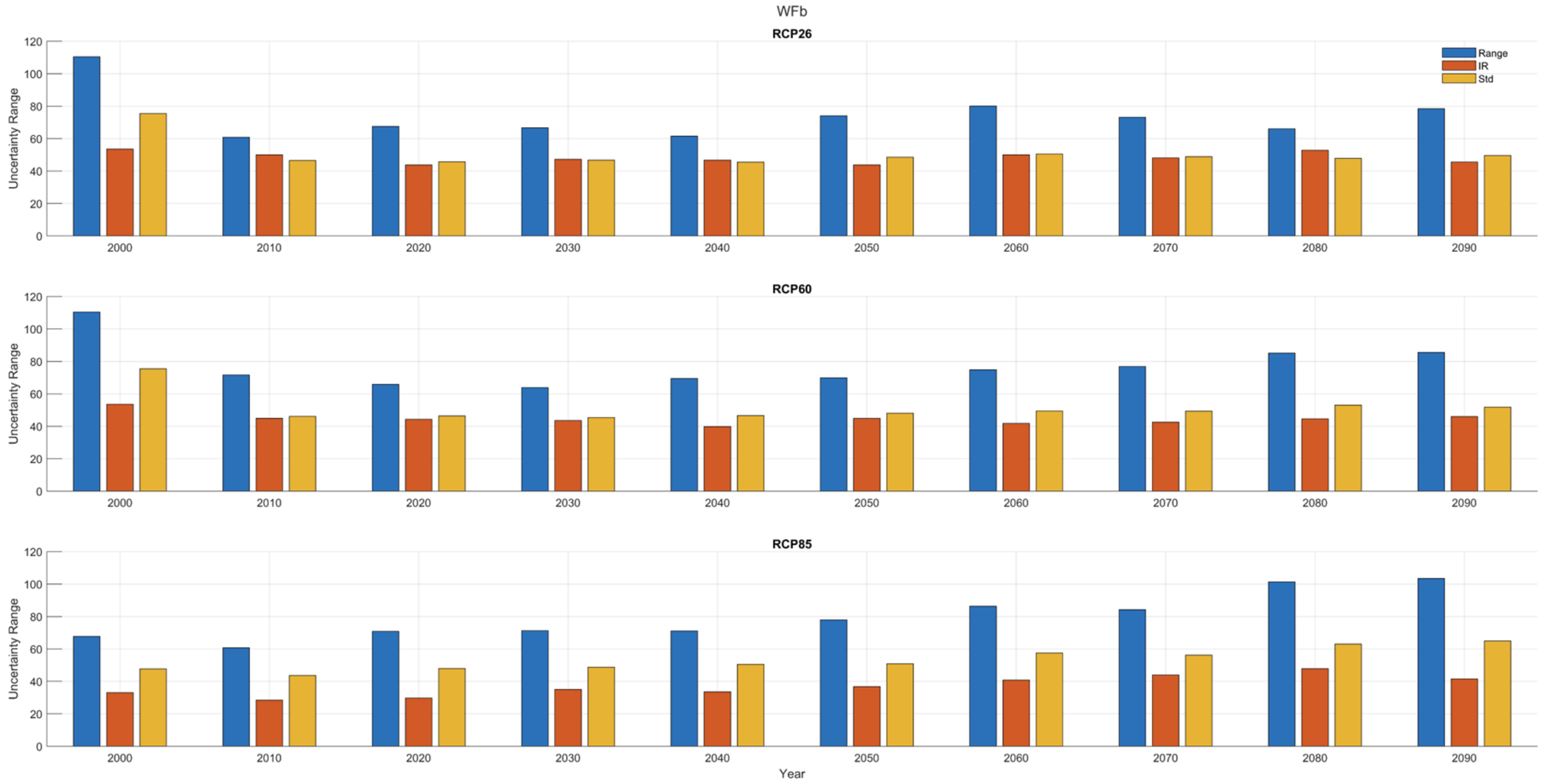

Figure R4: Comparative assessment of the uncertainty ranges, here shown for the global blue water footprint, under RCP 2.6 (top row), 6.0 (central row), and 8.5 (bottom row). The uncertainty ranges are computed for each year as the maximum-minimum difference (blue), the interquartile range (orange), and the standard deviation (yellow).

We have therefore revised Figure 1 by adopting the standard deviation for the shaded areas rather than the max-min range (see panels e-h of **Fig. R3** here), and modified the quantitative results accordingly, both the Abstract (lines 17-19) and in the Results section (lines 109-114), which now read:

Abstract

"[...] Results show that global average uncertainty ranges for the end-of-century projections exhibit a spread of approximately ~~28~~18% for green water (soil moisture) and ~~80~~51% for blue water (surface and groundwater bodies) footprints relative to the ensemble means. Blue water sustainability exhibits even larger variability, ranging from ~~416~~250% to ~~640~~451%, depending on climate scenarios and hydrological model choice. [...]"

Results

"[...] WF_g exhibits a narrower range of uncertainty (average inter-model variability across all RCPs of about ±~~14~~9% Fig. 1b, approximately ~~three~~2.8 times lower than the inter-model variability for WFb), suggesting more consistent model predictions for green water use compared to WFb. WDb exhibits far greater inter-model spread than WF metrics (see also crop-specific values in Fig. A1), with an average spread around the mean across all years (Fig. 1c) of ~~416~~250% (RCP 2.6), ~~420~~254% (RCP 6.0) and ~~640~~451% (RCP 8.5) (Tables S1--S4). [...]"

With regards to the last point raised by the Reviewer, to assess the robustness of the reported uncertainty patterns we conducted a leave-one-out analysis, recalculating the standard deviation while sequentially excluding each model from the ensemble. The resulting uncertainty estimates remain stable, with relative variations from the median between -6.4% and +1.3% across all variables, RCPs, and years (**Fig. R5** here).

Based on this, we have revised all the analyses presented in the manuscript accordingly. Together with the total uncertainty (fully addressed in point 1 above), we have consistently adopted the standard deviation rather than the max-min range also to quantify how uncertainties change with spatial resolution (**Fig. R6** below). In particular, we have adopted the coefficient of variation to assess the scale dependency of the uncertainty (lines 486-488, 512-515):

*"**Methods***

[...]

Uncertainty analysis

[...] To quantify the magnitude of uncertainty, we adopted the coefficient of variation (CV) defined as the standard deviation (σ) across all model estimates normalized by the multi-model ensemble mean:

$$CV = \frac{\sigma(V)}{\underline{V}}$$

where V denotes the considered variable (here, WF or WD) in each grid cell.

[...]

Lastly, to assess the scale-dependency of uncertainty (Fig. A3) we ~~define~~ computed the total coefficient of variation CV ~~as the relative spread between maximum and minimum model estimates (among all models) normalized by the multi-model ensemble mean (UR=[max(V)-min(V)]/mean(V)]~~ across spatial scales. The analysis was performed by progressively disaggregating values from the global mean down to the single grid cell, effectively convolving model outputs across increasing levels of spatial detail, through a moving window with dimensions equal to the scale considered."

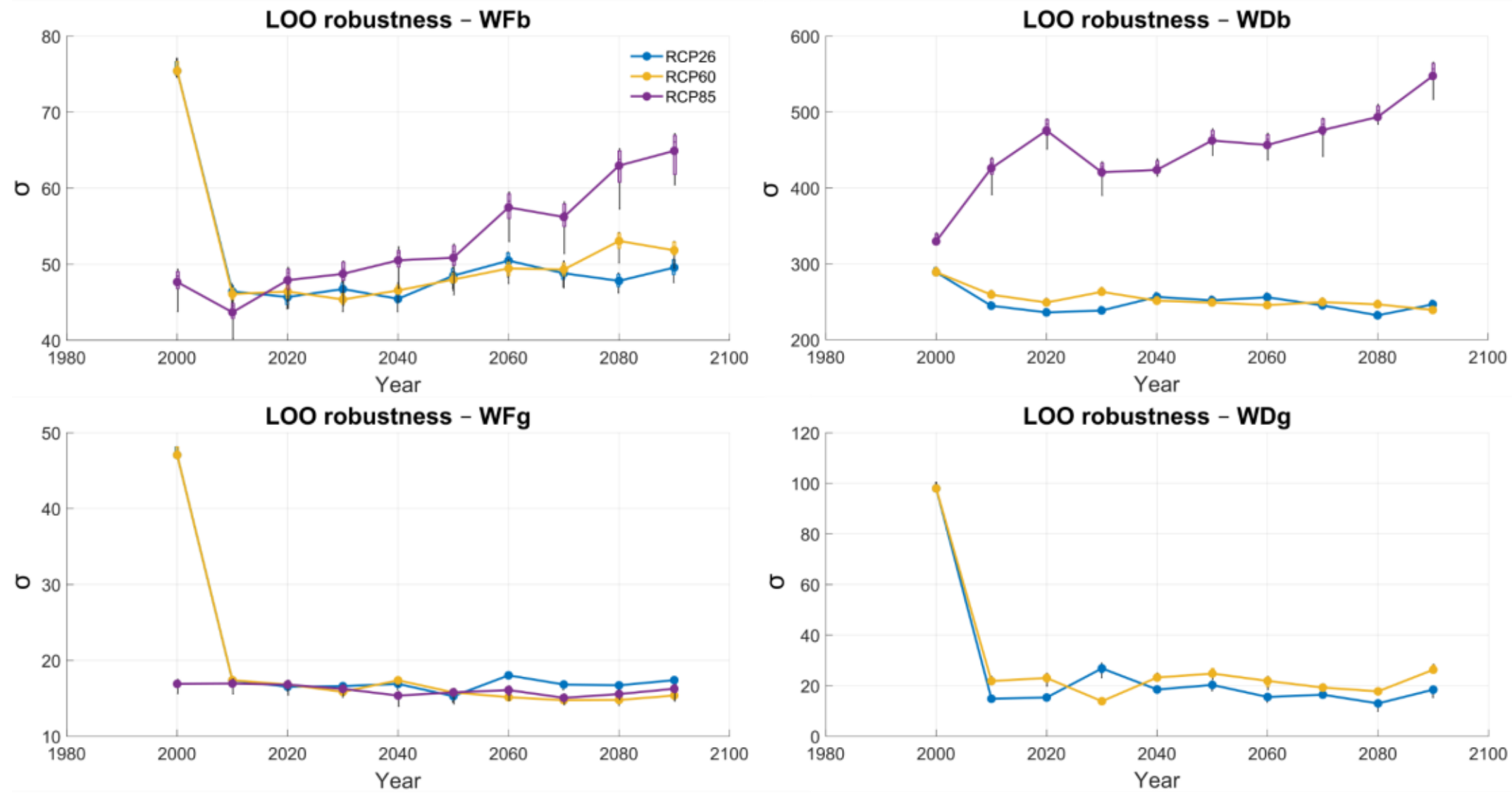

Figure R5 (new supplementary Fig. S17 in the revised manuscript): Robustness of the uncertainty metric, namely the standard deviation relative to year 2000 (σ, %), assessed through a leave-one-out (LOO) analysis. Panels show results for WFb (top left), WFg (bottom left), WDb (top right), and WDg (bottom right) WDg, under RCP 2.6 (blue), 6.0 (yellow), and 8.5 (purple). The continuous line represents the standard deviation computed using the full ensemble of models, while the error bars depict the distribution of uncertainty estimates obtained by sequentially excluding one model at a time. The limited spread of the boxplots relative to the reference line indicates that the estimated uncertainty remains stable when individual models are omitted. The maximum variability is found for WFb in the year 2090 under RCP 8.5 (median 66.06%, quartiles 61.82% and 66.92%).

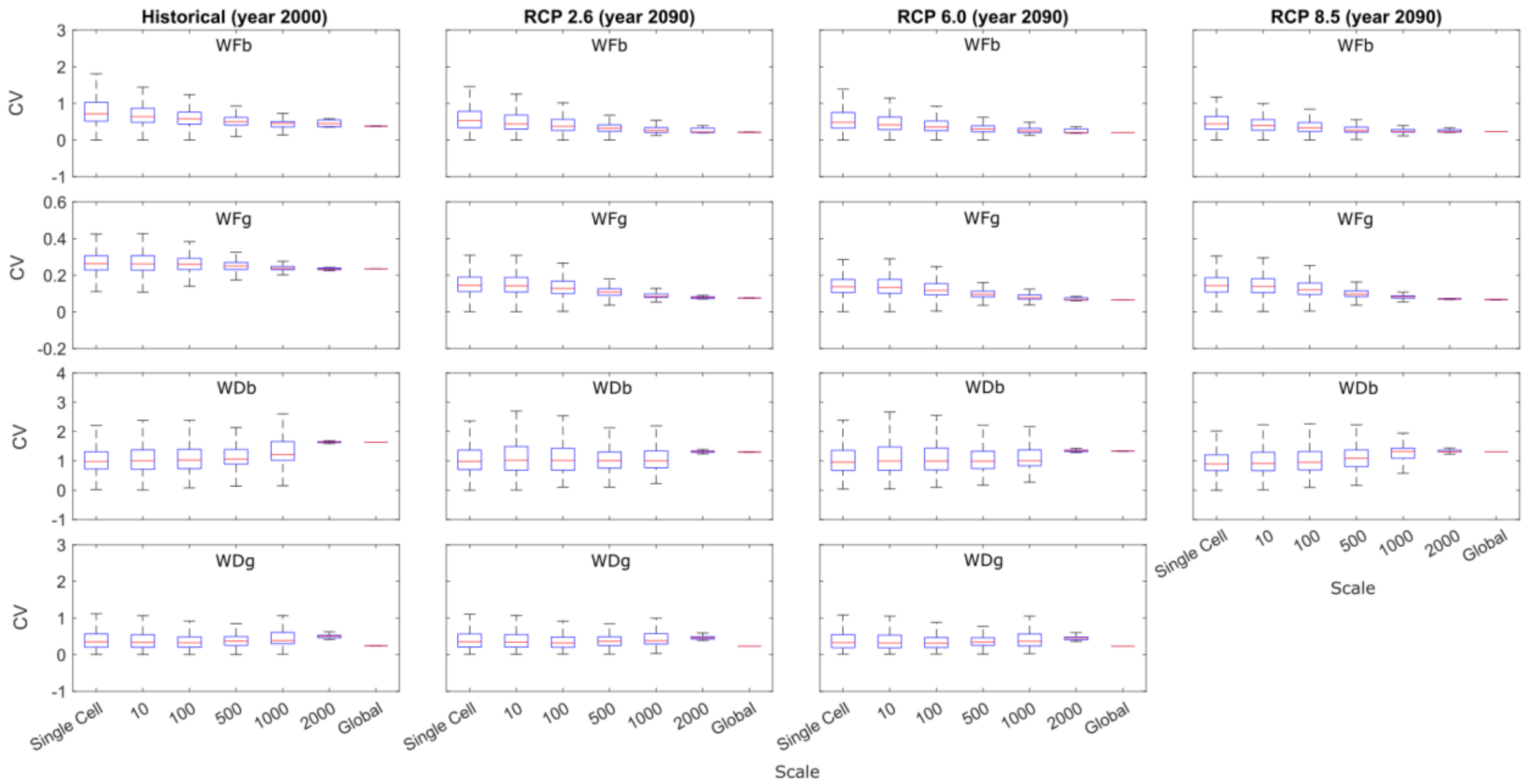

Figure R6 (same as Fig. A3 in the manuscript): Scale-dependency of the uncertainty. Total coefficient of variation among all water and climate models (for all the crops) as a function of the grid size (number of cells) considered, ranging from a single cell domain up to the to global scale.

3. The number of GWMs differs across RCPs, and key variables for WDg are missing in some scenarios. This limits comparability of uncertainty magnitudes across scenarios and water components. Please make this limitation explicit.

Reply: We thank the Reviewer for raising this point. We have now explicitly mentioned this limitation in the manuscript and broadened the discussion to address it, as also suggested by Reviewer #2 (point 12). The revised discussion (lines 240-249) now reads:

"[...] The relative contribution of GCM and GWM uncertainty should also be interpreted in light of the characteristics of the available model ensemble. Although in this study the number of GWMs (six) exceeds the number of GCMs (four), we observed that the overall uncertainty appears robust to the ensemble composition, as indicated by the limited sensitivity to the exclusion of individual models (Fig. S17). Model availability also varies across scenarios and variables within the ISIMIP2b framework. In particular, some GWMs do not provide the soil moisture renewability rate required to compute the green water debt for all RCPs (Tab. 1), which should be considered when comparing uncertainty magnitudes across scenarios and water components. Differences across GCM projections reflect both structural differences in model formulation and the contribution of internal climate variability. Further separation of these two contributions would require large ensembles from individual climate models (e.g., Lehner et al., 2020), which are not available within the ISIMIP2b framework used here.
The high spatial resolution of the analysis performed here further allowed us to [...]."

4. WD is a ratio involving renewable water availability. Near-zero availability will inflate WD and can dominate max–min uncertainty. Please specify how low or zero renewable water is handled (masking, lower bounds, or other QC), and quantify whether extreme grid cells drive reported spreads and hotspots. A simple sensitivity analysis for the WD threshold would strengthen the hotspot interpretation.

Reply: This is a very good point. Indeed, we considered a lower bound equal to 10^{-4} m/yr for the renewability rates, which was actually not initially mentioned in the manuscript. Motivated by the Reviewer's comment, we conducted a sensitivity analysis to test a range of minimum thresholds (from 10^{-1} to 10^{-6} m/yr) and evaluate their effects on (i) the resulting variability (i.e., standard deviation) in WD across models, and (ii) the fraction of grid cells affected (i.e., lost) by masking our data with such threshold. The result of this sensitivity analysis indicated that, at relatively high thresholds (> 10^{-3} m/yr), a large proportion of grid cells would be lost (**Fig. R7**), while at very low thresholds a small fraction of cells is affected, but the WD max-min range increases sharply due to the inclusion of too many low renewability rate values (**Fig. R7**, same as Fig. S16 in the revised SI). The threshold equal to 10^{-4} m/yr was selected as it represents a reasonable compromise between avoiding the exclusion of too many cells and preventing bias in the WD range estimate by including too many low renewability rate values.

In the revised version, we have included this analysis at lines 465-470:

"Renewability rates include runoff, groundwater recharge, and root-zone soil moisture, and were derived from [...]. To avoid unrealistically low renewability rates, which would lead to artificially high WD estimates, a minimum threshold of 10^{-4} m yr^{-1} was applied to R (with all cells below this threshold excluded from the analysis). This value was selected as a compromise between minimizing the number of grid cells excluded from the analysis and limiting the bias in the WD variability estimates associated with the inclusion of excessively low renewability rates (see sensitivity analysis in Fig. S16).

WF and WD were derived from [...]."

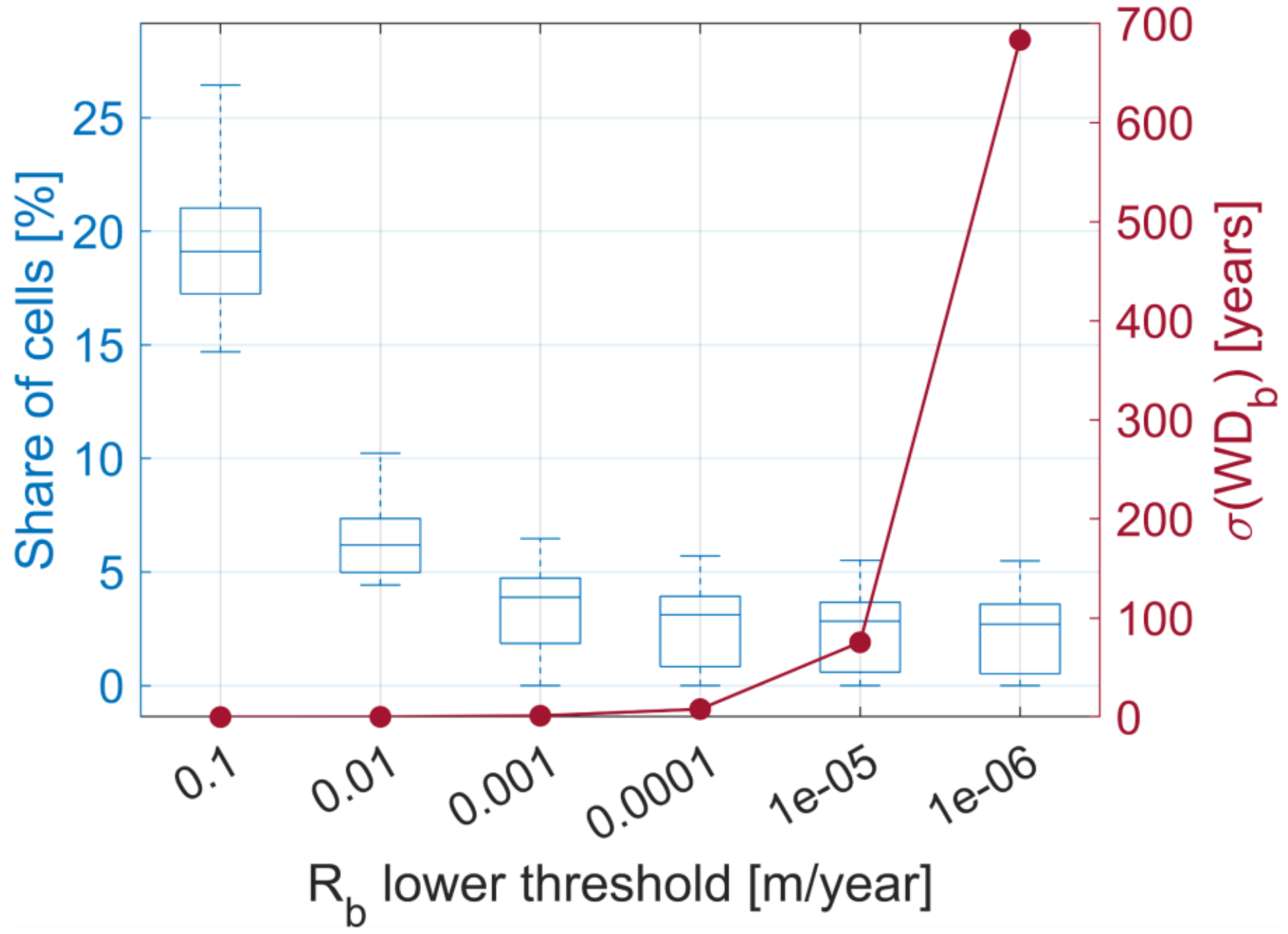

Figure R7 (same as new supplementary Fig. S16). Sensitivity of blue water debt to minimum thresholds applied to water renewability rates. Unrealistically low values of surface runoff and groundwater recharge were filtered using a range of thresholds (x-axis). For each threshold, the percentage of global cells affected by each value (left y-axis, in blue), and the variability of WD_b (expressed as standard deviation - right y-axis, in red) were evaluated. Results are aggregated across all crops, model combinations, and the entire 10-year period.

Minor:

5. Crop differentiation relies on fixed crop coefficients and static cropping calendars. This is acceptable for global assessment. However, the implications of ignoring adaptive phenology and management should be more explicitly stated.

Reply: We thank the Reviewer for this comment, with which we agree. We have expanded our discussion to address this as follows (lines 272-277):

"[...] While these projections are valuable, they should be interpreted with care, as this study relies on business-as-usual scenarios, assuming fixed socio-economic conditions at 2005 levels (Frieler et al., 2017) and not incorporating mitigation efforts or projected changes in management practices, crop production (yields, harvested areas, or growing season) driven by technological advancements, land reform policies, irrigation expansion, food demand or market dynamics. In addition, crop differentiation in this study relies on fixed crop coefficients and static cropping calendars, not explicitly representing crop phenology and temporal variations in management practices. While such assumptions are common in global-scale assessments, they neglect adaptive responses of agricultural systems -- such as shifts in sowing dates or cultivar choices -- that can substantially influence crop growth periods and phenological trends (Zhang et al., 2022) and alter simulated crop performance compared with fixed-calendar assumptions (Minoli et al., 2022). This approach, however, allows us to detect locally critical areas which may face aggravated climate-driven stress in the future [...]."

Reviewer #2

This study assesses inter-model uncertainty in projected crop water demands across an ensemble of global climate and water models. Results show generally larger inter-model variability for blue water, which is dominantly driven by GWMs, and smaller inter-model variability for green water, which is dominantly driven by GCMs. The article is a useful contribution to this literature on the sustainability of future crop water requirements, in which inter-model uncertainty is generally large and under-characterized. The approach is overall sound, and the results shown generally support the results.

That said, there are several reasonably important methodological and clarity problems that need to be addressed before this article can be published. The main issues are that the definition and quantification of 'uncertainty' is vague in the intro and methods, and the measures used are technically incorrect and not sufficiently firmly grounded in formal statistics. For several key steps in the analysis (notably the renewability rate calculations), the rationale and justification for the methods is absent and we get only references to other papers. I looked at some of the papers and did not find the necessary explanations. These steps need to be clarified for me to assess whether the results are sound and meaningful. All these concerns are enumerated in the line comments below, and I think they should all be addressable within the paper's existing framework.

> **Reply**: We thank the Reviewer for the positive assessment of our work and the detailed and constructive feedback, which greatly helped us improve the manuscript in terms of clarity and preciseness. In the revised version, we carefully addressed all the Reviewer's points, as detailed in our comments below. Specifically, we have (i) clarified and strengthened the definition and quantification of uncertainty throughout the manuscript, adopting more statistically grounded approaches and revising all analyses and Methods accordingly, (ii) improved the transparency and justification of key methodological steps (e.g., renewability rates), including additional explanations in addition to references, (iii) revised the Introduction, Results, and figures to enhance clarity and organization, include more explicit definitions (e.g., of WF and WD), and improve figure readability, (iv) expanded the explanation of key results by providing additional mechanistic insights into the emergence of hotspots, and (v) ensured full reproducibility by making the code to reproduce the analyses available.
>
> In the revised manuscript, the changes are visible thanks to the track-change mode. Whenever possible, we also copied here extracts of the modified text below the corresponding reply (highlighted in blue the new text additions, strikethrough text in red for deleted text). Line numbers reported for these excerpts refer to the version displayed in track-changes mode.

1. Intro – layout why this uncertainty matters.

> **Reply**: Thank you for the suggestion, which greatly helped to strengthen our Introduction. We added the following to better address why quantifying uncertainty matters (lines 61-76):
>
> *"[...] However, these models often yield divergent estimates of the aforementioned variables due to multiple sources of uncertainty along the climate-hydrology modeling chain. In this study, uncertainty is understood as the incomplete and imperfect information about climate forcings and hydrological processes, which leads to probabilistic rather than deterministic*

estimates of crop evapotranspiration and water use. A major component is epistemic uncertainty, ~~which is related to many aspects of the considered modeling framework, such as~~ arising from differences in model structures, assumptions, and parameterizations, resulting in varying estimates of relevant hydrologic variables (Walker et al., 2003; Zarei et al., 2024). Additional uncertainty stems from the spread in climate projections (Lehner et al., 2020), reflecting both epistemic uncertainty related to various climate model formulations and scenario uncertainty associated with future socio-economic trajectories. Moreover, the inherently chaotic nature of the climate system contributes to internal variability, further compounding these uncertainties (Haddeland et al., 2011; Schewe et al., 2014). For crop water use indicators, these uncertainties propagate into output estimates, as demonstrated in water footprint and crop water use studies at basin to global scales (Zhuo et al., 2014; Mialyk et al., 2024). This uncertainty is critical because it directly affects the reliability of sustainability assessments: overestimating water availability or underestimating crop water demands may lead to overly optimistic conclusions, while ignoring spread in model predictions may conceal regions facing critical water stress or unsustainable water use. Explicitly accounting for these uncertainties can support more robust and informative estimates of crop water use, enabling better-informed water management decisions. This study aims to [...]."

2. L42 individual crop responsibility – not sure I understand the term. I think you mean attribution of aggregate water requirements for an area to different crops that might be grown there, which is obviously policy relevant information?

Reply: We have adjusted the sentence to clarify the concept. This now reads (lines 43-44):

"The majority of these indicators are effective for broad-scale assessments, but they often lack a direct physical basis and are limited in their ability to ~~assess individual crop responsibility~~ attribute water use and impact to individual crops within a given area (Tuninetti et al., 2019)."

3. L43-46: Given the centrality of WD and WF to the analysis, expanding a bit on the meaning of these two metrics could ease interpretation of the whole paper. I did not find further explanation in the Methods. How are they different/similar? Does the distinction between mass (volume) vs time basis give different impressions of water requirements? A couple extra sentences on this would be enough. It could also be explained further why these two metrics are more physical (you compare to threshold-based definitions, but to me thresholds can easily be physically based and crop-specific).

Reply: Thank you for this suggestion. Indeed, WF and WD are central concepts in our study – we have therefore extended both the Introduction and the Methods to provide additional insights into these two metrics.

Introduction (lines 47-50):

"To overcome such limitations and account directly for crop water use, two metrics have been proposed, namely the water footprint (WF), defined as the total volume of freshwater used to produce a certain crop (Hoekstra et al., 2007; Hoekstra et al., 2011; Mekonnen et al., 2011), and the water debt repayment time (WD), which is the time required to replenish water resources used for annual crop production (Tuninetti et al., 2019). While WF quantifies the absolute magnitude of water use per year (e.g., in $m^3 yr^{-1}$), WD relates this consumption to

local hydrological renewability rates, translating volumes into a timescale that indicates whether crop water use exceeds the rate at which resources are naturally replenished on a yearly basis. Each of these indicators can be further assessed based on the different sources of water used by crops, namely the irrigated quota from surface water bodies and groundwater ("blue water") and soil moisture ("green water"). For the quantification [...]."

Methods (lines 470-476):
"WF and WD were derived from the same underlying crop water use(evapotranspiration) estimates but differ in their interpretation. WF quantifies the absolute volume of freshwater consumed annually to sustain crop production, whereas WD expresses this consumption relative to the long-term renewability rate of the corresponding water source. Consequently, WD provides a direct measure of sustainability: values ≤ 1 year indicate that annual crop water use can be replenished within one hydrological cycle, while values > 1 year indicate that water is being depleted faster than it is renewed (unsustainable), thus requiring more years to be replenished."

4. L78-80: I wouldn't call this output a byproduct – its inherent to the problem you are trying to solve. Without characterizing model ensemble uncertainty, we have no idea how likely the water sustainability projections of a given model are to actually arise. The likelihood is a key piece of information, beyond the projections of single models, that is essential to characterizing what the future is likely to look like.

__Reply__: We see the Reviewer's point and we have rephrased the paragraph accordingly (lines 92-94):

"Performing a multi-model analysis allows us to quantify the uncertainties in the estimation of indicators for crop-water requirements and sustainability associated with different GCMs and GWMs, their propagation in future scenarios and across spatial scales of analysis, as well as to pinpoint the major sources of uncertainty and their spatial distribution. ~~As a by-product of the uncertainty analysis,~~ Characterizing this ensemble uncertainty is essential to assess the robustness and likelihood of projected changes in water sustainability, beyond the outcomes of individual models. In this context, our work further ~~allows~~ enables the identification of hotspot regions and crops that are likely to experience severe transitions to unsustainable water use in the future. Quantifying uncertainties embedded in crop water use and sustainability estimates and tracing them back to their main sources is crucial for providing a clear understanding of potential risks, improving future predictions, and enabling the formulation of targeted strategies to manage water resource sustainability in the face of climate change."

5. L100: Is this lack of trend for the ensemble mean or individual models? I think it is for the ensemble mean, which is perhaps not the most meaningful context-setting result in a study focused on inter-model variability. Is there a subset of individual models that have significant trends under lower emissions scenarios? And vice versa?

__Reply__: Yes, the statistically non-significant increasing trend emerging for RCP 2.6 and 6.0 in Figure 1c refers to the ensemble mean (solid blue and yellow lines). Indeed, the main purpose of the figure was to highlight the range of variability among all models, here originally

represented using the min-max envelope, and its evolution over time, rather than to emphasize model-specific trends. We have also verified that, when individual models are considered separately, none exhibits statistically significant trends under these scenarios, as shown in **Fig. R8** below (same as Fig. A2 in the manuscript). We have clarified this in the revised text at lines 117-120:

"We further note that the ensemble mean (across all models) of WD_b only shows a significant increasing trend (Mann-Kendall test, $p<0.05$, Tab. S1) under the most severe climate scenario (RCP 8.5), while remaining relatively constant under RCPs 2.6 and 6.0 (Fig. 1c). No statistically significant trends emerge for individual models under these scenarios (Fig. A2). Despite relying on [...]".

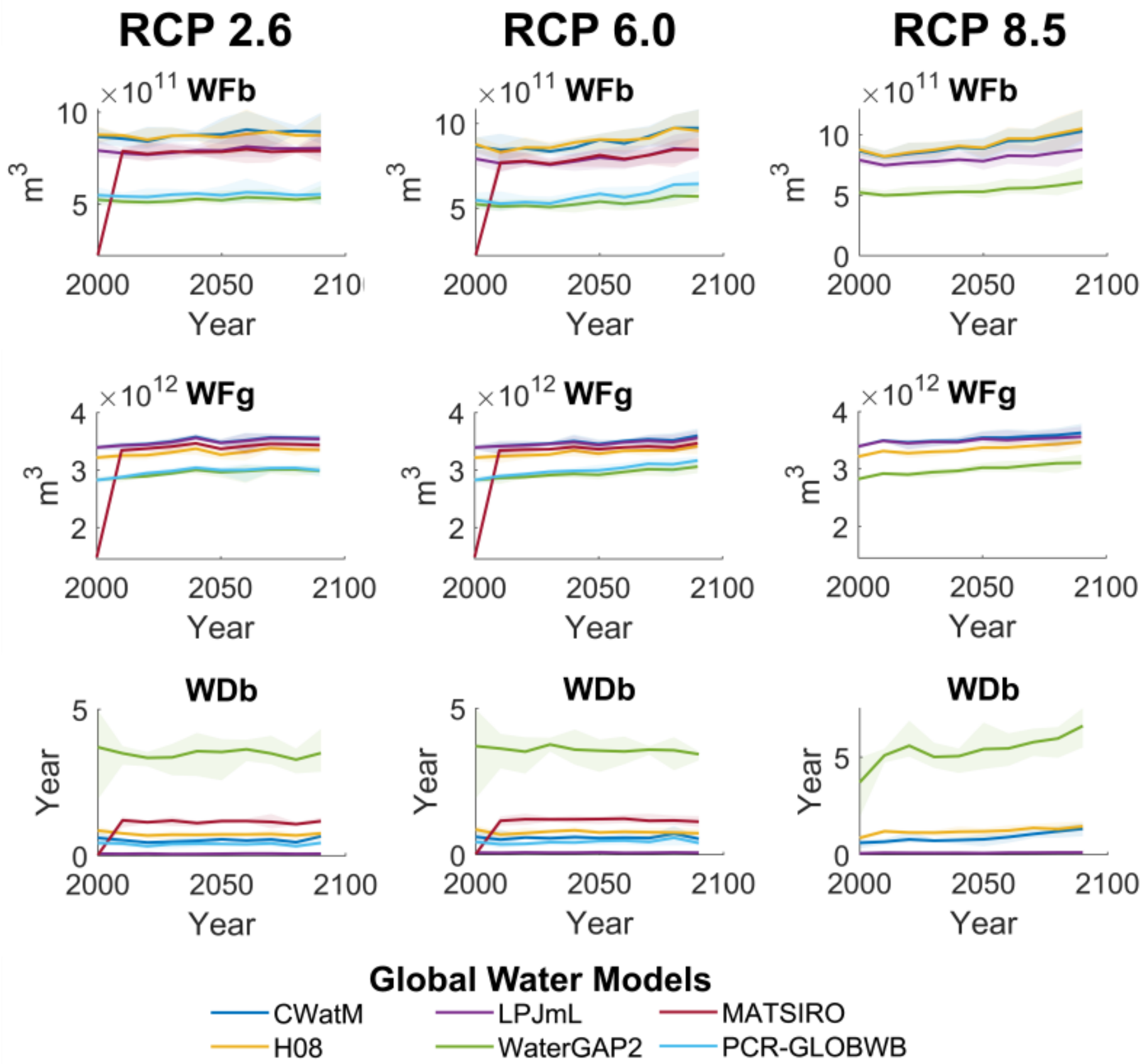

Figure R8 (same as Fig. A2 in the manuscript): Temporal evolution of global water footprint and water debt for different GWM. Global blue water footprint WF_b, green water footprint WF_g, and blue water debt WD_b under the three RCPs, and for each of the six GWMs (averaged for the four GCMs), considering all crops. MATSIRO and PCR-GLOBWB models are not available for RCP 8.5.

6. L110: Consider clarifying what you mean by 'robust' for fractional uncertainty < 1

Reply: Thank you for pointing this out. We have clarified the meaning of "robust" in the context of the coefficient of variation (CV) - which was adopted as a more statistically justified metric in the revised manuscript, following the Reviewers' comments (see also reply to point #19 below, and points 1 and 2 from Reviewer #1). The revised section (lines 127-134) reads:

"To pinpoint specific locations where WF and WD assessments may be more unreliable, we map the spatial distribution of projected ~~fractional uncertainty~~coefficient of variation, ~~F~~CV, in 2090 (RCP 8.5) (Fig. 2). CV is defined as the ratio between the variability (standard deviation)

across all model estimates and the corresponding multi-model mean (Methods). Therefore, values of CV ~~This metric compares the uncertainty magnitude relative to the signal itself, with F~~ < 1 indicate~~ing robust estimates across models, while F > 1 implies high uncertainty (see Methods for details)~~ that the ensemble spread is smaller than the mean signal, implying that model agreement is relatively strong, while CV > 1 denotes that the variability across models exceeds the mean, indicating low agreement and thus higher uncertainty."

7. L112-119: There are two things to explain here. 1) Why is model spread for WDb so much larger than signal compared to WF? 2) Regions with F>1 for WF align closely with hotspots of high F for WDb. Why those regions? You refer to the SI figures at the end of this paragraph, but the interpretation is not clear. Those figures show variability between crops – are differences between crops driving these two things? A bit of mechanistic expansion might be illuminating.

__Reply__: Thank you for pointing this out. After having provided further details on the (updated) coefficient of variation (see point 6 above), we have substantially revised this paragraph, in line also with the following comment. In particular, we have better explained why the spread for WDb is larger than that for WF and provided a clearer mechanistic explanation for this behavior. The revised paragraph (lines 127-148) now reads:

"To pinpoint specific locations where WF and WD assessments may be more unreliable, we map the spatial distribution of projected ~~fractional uncertainty~~coefficient of variation, ~~F~~CV, in 2090 (RCP 8.5) (Fig. 2). CV is defined as [...] uncertainty.
For WF, most regions exhibit ~~F~~CV<<1 (Fig. 2a), indicating a relatively low level of uncertainty compared to the variable's magnitude and suggesting that results are broadly robust, as the uncertainty does not dominate the signal. ~~A reversed situation occurs for WDb, for which a fractional uncertainty exceeding 1 almost everywhere (with the highest values in Eastern Australia, North-Western India, and Central USA) advises a significant variability in the results (Fig. 2b). Such a divergence stems from disagreement in climatic and renewability rates data and highlights regions where crop water estimates are particularly sensitive to model choice (see also Tables S2--S5 for crop-specific global values of WF and WDb, and Figures S4--S7 for the corresponding maps).~~ In contrast, WDb exhibits higher CV values, reflecting the much larger variability of the sustainability metric already highlighted above. This difference arises from the formulation of WDb as the ratio between water demand and blue renewability rates. In fact, while WF primarily depends on precipitation and potential evapotranspiration, which are relatively consistent across models (Figs. S10--S12), WDb is strongly influenced by runoff and groundwater recharge, which exhibit large inter-model variability due to different process representations in hydrological models (Telteu et al., 2021). As a result, variability in WDb across GWMs might exceed the magnitude of the mean signal itself, leading to CV>1 in certain regions, such as around Eastern Australia, North-Western India, and Central USA (Fig. 2b), thus highlighting areas where crop water estimates are particularly sensitive to model choice (see also Tables S2--S5 for crop-specific global values of WF and WDb, and Figures S4--S7 for the corresponding maps). A closer look at the spatial distribution of [...]."

We also note that, due to the introduction of CV, which is a more statistically justified metric (points 1 and 2 by Reviewer #1, and point 19 by Reviewer #2), we no longer observe regions where the coefficient of variation (previous F) is greater than 1 for WF.

8. L120-126: Ah ok here is more explanation of why these regions. It may improve organization to group expansion on point 1 above into a single paragraph, then group regional hotspot discussion (point 2) into a separate paragraph. Glancing between the panels, I think I mostly agree with the spatial correspondence between F for WDb and the magnitude of WDb itself. A more rigorous approach could be to plot WDb vs F(WDb) – the corresponding regression statistics would give a clear sense of how strong this spatial dependence is (maybe in the SI as this figure already has a lot of data).

__Reply__: In line with the previous reply, we have revised the entire paragraph (lines 127-148) accordingly. As noted in our reply to the previous point (n.7), we have reorganized this section as suggested by the Reviewer.
Regarding the correlation between WDb and its associated uncertainty, we thank the Reviewer for this helpful suggestion. To quantitatively assess the spatial correspondence between the two variables, we performed a grid-cell level analysis and added a new supplementary figure (Fig. S9, same as **Fig. R9** here). The results indicate a positive, albeit relatively weak, correlation ($R^2 \cong 0.2$), reflecting that areas with higher water debt tend to exhibit higher relative uncertainty, although such conditions are confined to localized hotspots rather than the majority of the domain. This additional analysis has been incorporated in the revised manuscript (lines 156-158).

"A closer look at the spatial distribution of projected WDb [...]. Significant hotspots, for instance, are observed in the Central USA and along the Pakistan-India border, where the dominant crops in each region contribute to exceeding sustainable levels (potatoes, barley, and maize in the former; wheat, rice, and sugarcane in the latter; see insets in Fig. 2c). The positive but relatively weak correlation between WDb and CV(WDb) (slope of the linear fitting < 0.05, $R^2 \cong 0.77$, Fig. S9) reflects the fact that, while high water debt is associated with higher uncertainty, such conditions are confined to spatially limited hotspots."

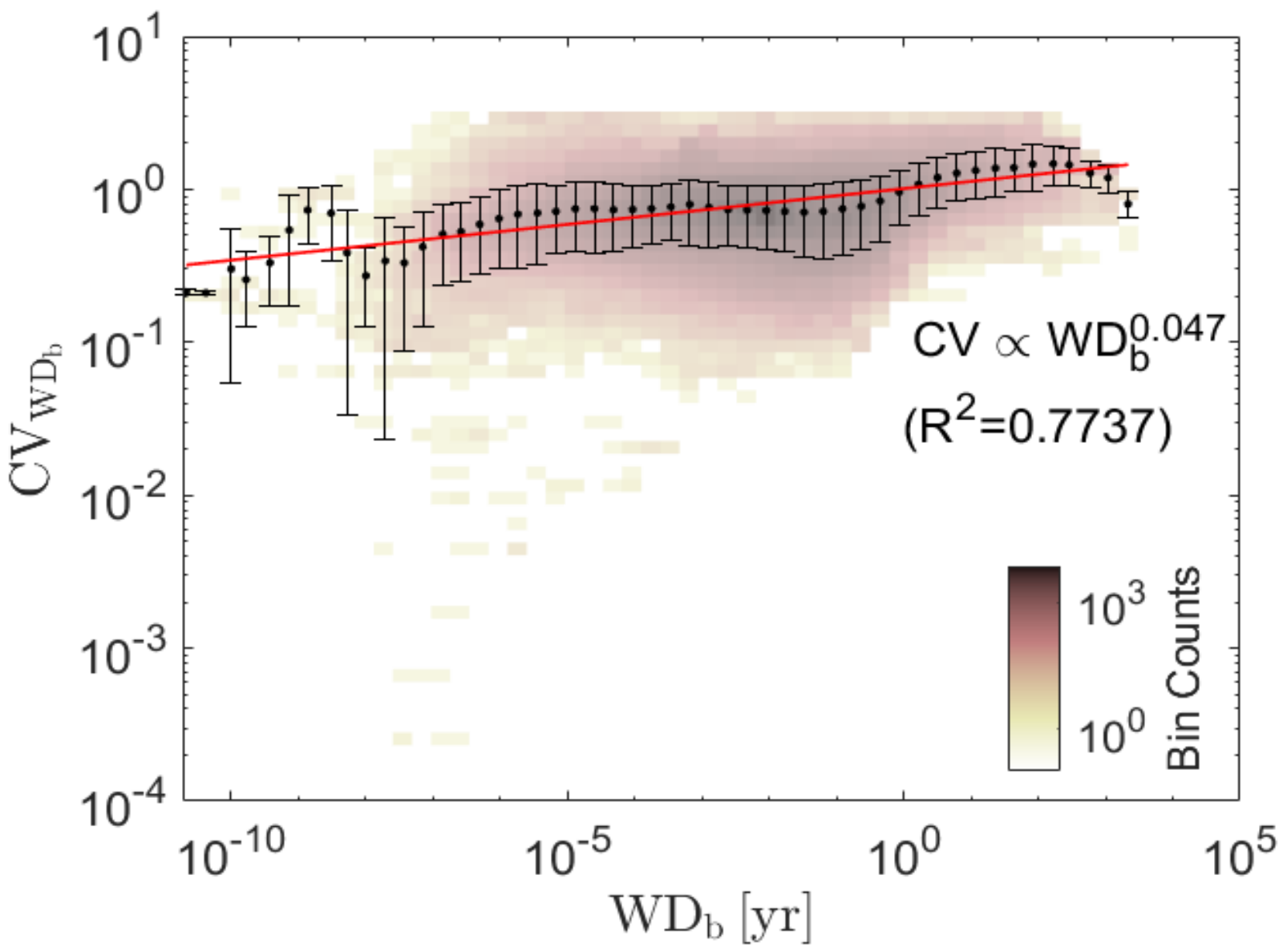

Figure R9 (same as new supplementary Fig. S9 in the manuscript): Bi-logarithmic grid-cell relationship between the blue water debt (WDb) and its associated coefficient of variation (CV), binned to increase readability (colors code the number of data points in each bin). The red line represents the linear regression ($R^2 \cong 0.77$) fitting the average of each bin (black dots).

9. L126: So is the interpretation here that model differences have a larger impact on total uncertainty in regions where both 1) total crop water demands are high and 2) crop water demands are highly concentrated in a small number of crops? Why is that? An extra sentence on interpretation would help.

Reply: We have revised the full paragraph following the Reviewer's suggestions (see also replies to comments 6-8) and added a broader explanation for the observed results, which are now updated with the new uncertainty metrics. The reason for the emergence of such hotspots (related to over-exploitation of aquifer systems) is provided in the revised paragraph (see lines 127-148 above) and in the discussion of Fig. 2c (lines 149-156):

"A closer look at the spatial distribution of projected WDb further highlights that a subset of such regions is also characterized by significant pressure on water resources, and thus, high values of WDb (Fig. 2c). These hotspots emerge across all RCP scenarios (Fig. S8) and correspond to areas with the highest rates of groundwater depletion (Aeschbach et al., 2012), where local and regional aquifer systems are over-exploited (Gleeson et al., 2012), thus affecting groundwater recharge. Significant hotspots, for instance, are observed in the Central USA and along the Pakistan-India border, where the dominant crops in each region contribute to exceeding sustainable levels (potatoes, barley, and maize in the former; wheat, rice, and sugarcane in the latter; see insets in Fig. 2c). [...]"

10. L134: The paper below on CO_2-vegetation effects on runoff has a similar argument regarding the influence of spatial averaging on uncertainty estimation. It could be a useful reference: https://www.nature.com/articles/s44221-024-00361-z

Reply: Thank you for suggesting this reference, which also makes a point on inter-model variability. We have included it at line 166:

"[...]. This occurs because local-scale projections retain the full magnitude of inter-model differences, whereas aggregation across larger areas averages out opposing deviations, leading to lower apparent uncertainty at coarser scales, as also noted in hydrological impact studies (Lesk et al., 2025). In contrast, [...]."

11. L139-141: A stylistic point but I think this transition is very neutral misses an opportunity to express why it is important to figure out the origins of uncertainty (i.e. draw a clearer link with the preceding section).

Reply: We thank the Reviewer for this constructive comment. The beginning of the subsection has been revised to establish a clearer link with the preceding results and to better motivate the need to disentangle the respective contributions of climate and water models to the overall uncertainty (lines 171-175):

"Disentangling the relative roles of global climate and water models

~~The results presented above already highlight substantial variability across different GCM and GWM combinations. We now turn to dissecting the sources of this variability and focus on~~

~~identifying what is driving the observed differences across scales and locations.~~ The preceding analyses highlight substantial variability across different GCM and GWM combinations. Understanding the origins of this variability is crucial to determine the robustness of crop water use and sustainability estimates. We therefore turn to disentangling the relative contributions of GCMs and GWMs to the total variability across scales and locations.
At the global and continental scales [...]."

12. L146-147: There are only 4 GCMs used in the study, compared to 6 GWMs. Might you expect having 50% more GWMs to give you a greater range simply because of the sample difference? It's also worth noting that any 2 GCMs run with the same forcing can give different answers for 2 reasons: differences in model assumptions or process representation, and chaotic internal variability. Here they are lumped together. The way to solve that is by isolating internal variability using large ensembles from a single model. That might not be possible given limited models participating in ISIMIP, but this problem should be noted and interpreted.

Reply: We thank the Reviewer for raising this point (which was also noted by Reviewer #1). This is indeed a limitation of the analysis here which was not discussed in our original manuscript. We have now broadened the discussion around this topic, by explicitly stating the implications of the unequal number of GWMs and GCMs in the ensemble, the incomplete model availability across scenarios and variables, and the role of internal climate variability in shaping GCM spread. The revised text (lines 240-249) now reads:

"[...] The relative contribution of GCM and GWM uncertainty should also be interpreted in light of the characteristics of the available model ensemble. Although in this study the number of GWMs (six) exceeds the number of GCMs (four), we observed that the overall uncertainty appears robust to the ensemble composition, as indicated by the limited sensitivity to the exclusion of individual models (Fig. S17). Model availability also varies across scenarios and variables within the ISIMIP2b framework. In particular, some GWMs do not provide the soil moisture renewability rate required to compute the green water debt for all RCPs (Tab. 1), which should be considered when comparing uncertainty magnitudes across scenarios and water components. Differences across GCM projections reflect both structural differences in model formulation and the contribution of internal climate variability. Further separation of these two contributions would require large ensembles from individual climate models (e.g., Lehner et al., 2020), which are not available within the ISIMIP2b framework used here.
The high spatial resolution of the analysis performed here further allowed us to [...]."

13. Fig. 3: I find it hard to compare values in this figure. My eye is drawn to the size of the dots and it's hard to simultaneously compare the colors, which is the data we care about. Consider rethinking the visualization here so that the key message (WF range, not continental gross production) is more visually dominant.

Reply: Thank you for this suggestion - we agree with the Reviewer that the key message of the figure could be more clearly emphasized. Accordingly, we have revised Fig. 3 (see **Fig. R10** below) by replacing differently sized bubbles with squares of the same size. The information on production for each continent has been removed, as it was not central to the interpretation of the results and potentially distracted from the main comparison (represented by colors).

For consistency, we have also revised Fig. A4 showing the same analysis for WDb (**Fig. R11** here).

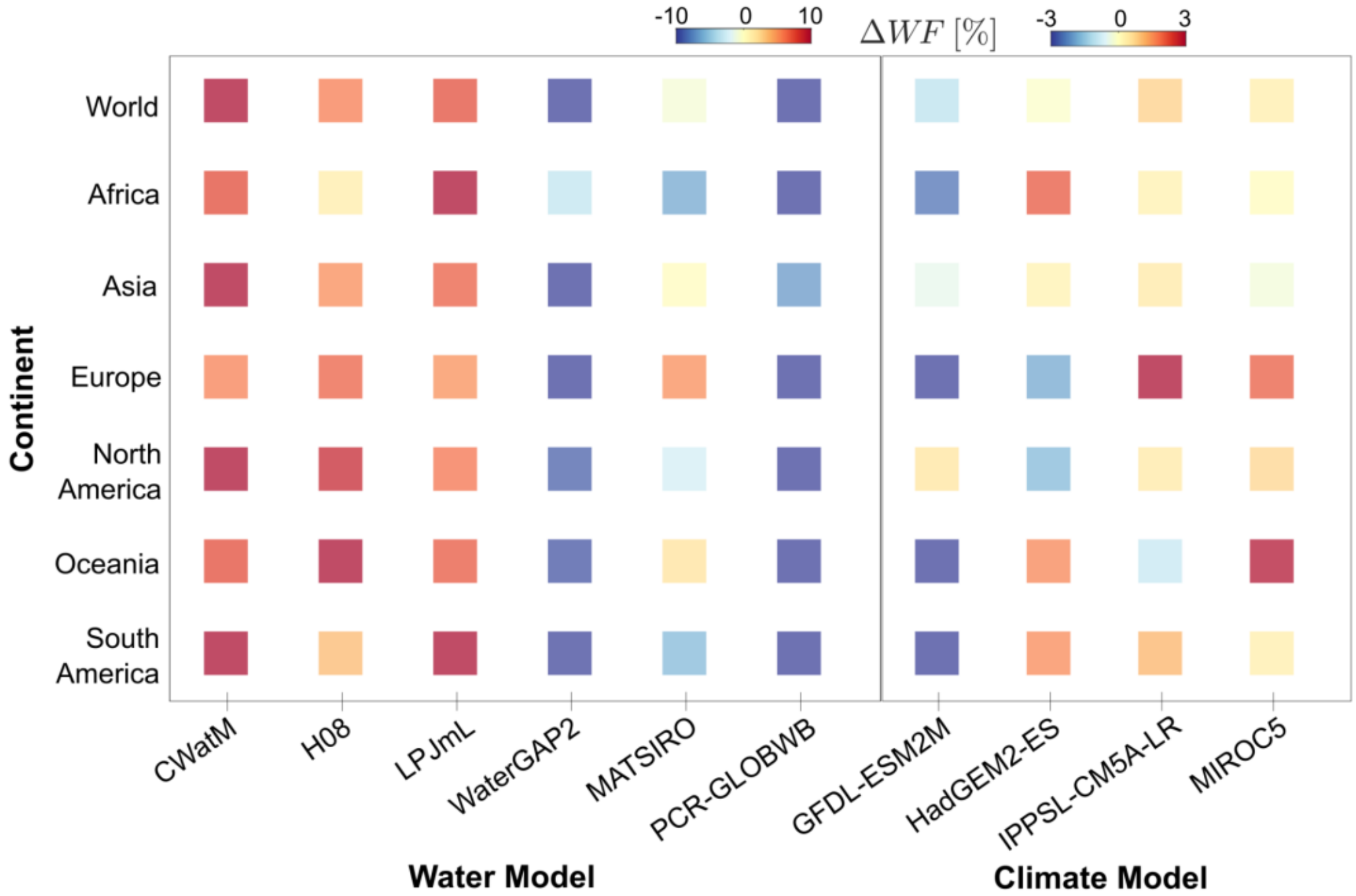

Figure R10 (same as new Fig. 3 in the manuscript): Sources of uncertainty in global and continental scale crop water footprint. Variability of global and continental scale total water footprint WF (blue plus green) across GWMs (left panel) and GCMs (right panel) averaged for all years between 2000 and 2090 and all crops (here future projections refer to RCP 6.0, for which all models are available, see Tab. 1). Colors code the relative deviation of WF with respect to the average value per continent *C*: $\Delta WF = [(WF_{C,m} - \underline{WF_C})/\underline{WF_C}] \cdot 100$, *m* being the considered model. The same analysis for WDb is shown in Fig. A4.

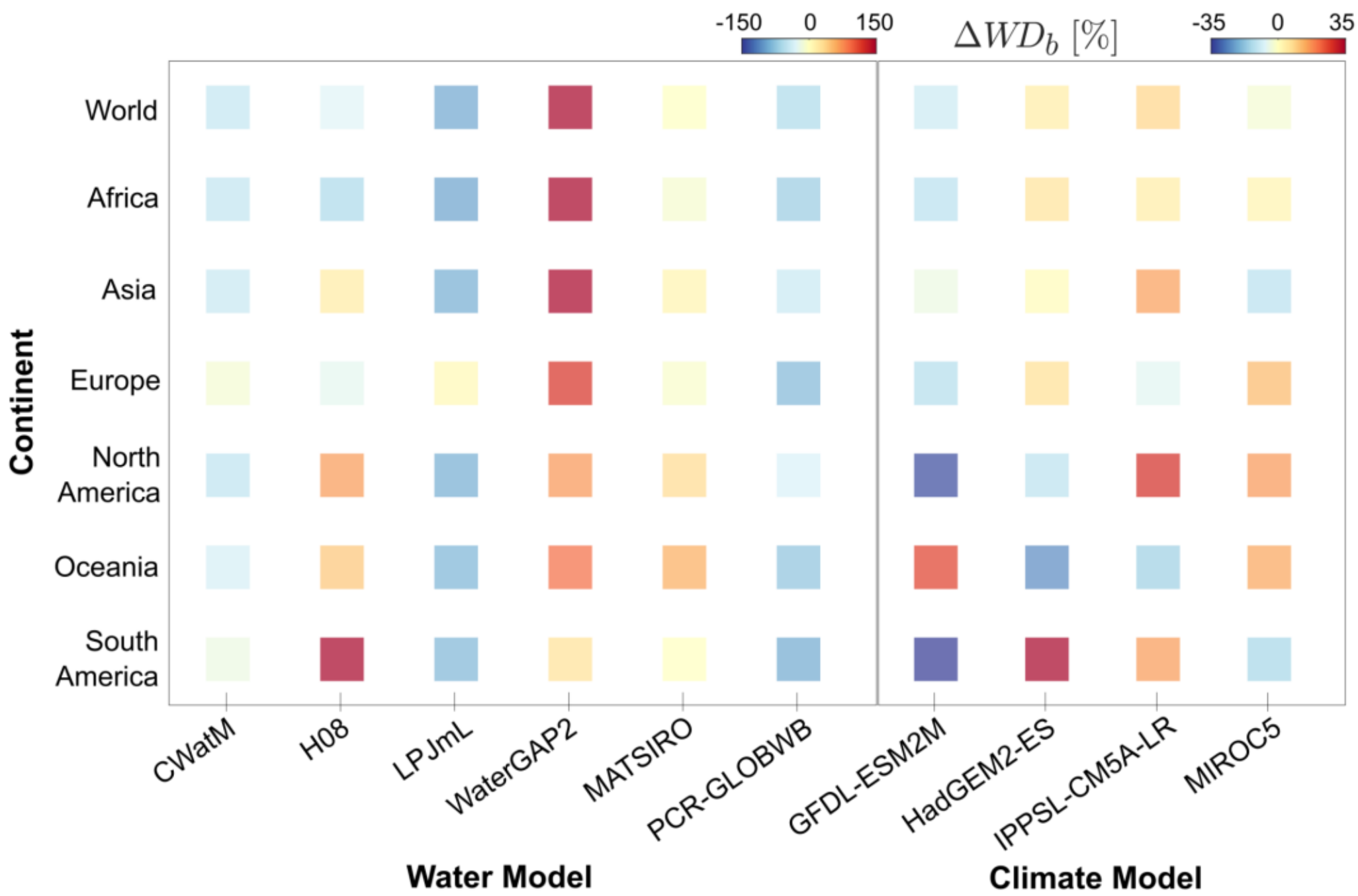

Figure R11 (same as Fig. A4 in the manuscript): Same as Fig. R10 but for blue water debt (WDb).

14. Regarding CO2 physiological effects: Is the effect of CO2 on stomatal conductance, crop water demand, and renewability rates included in the GWMs? It should be by default in the GCMs, and ideally this should be consistent across the modeling chain. Perhaps different physiology representations in GWMs is part of why the range of projected PET is so large?

Reply: This is a good point - thank you. Indeed, differences in how stomatal conductance and vegetation water use respond to rising CO_2 concentrations constitute a relevant source of structural uncertainty in hydrological projections. In ISIMIP2b, all models are driven by consistent CO_2 concentration trajectories. However, while the GCMs used to force the impact models generally include CO_2 effects on stomatal conductance and evapotranspiration in their land-surface schemes, the representation of vegetation physiology is not consistent across all the GWMs employed in our study. In particular, LPJmL and MATSIRO explicitly simulate CO_2-induced changes in photosynthesis and stomatal conductance (Reinecke et al., 2021), thereby allowing the variables that we use (potential evapotranspiration PET, surface runoff, groundwater recharge, and root-zone soil moisture) to respond to both radiative and physiological forcing. By contrast, the remaining GWMs in our ensemble - namely WaterGAP2, CWatM, PCR-GLOBWB, and H08 - do not explicitly account for CO_2 driven stomatal regulation, and therefore their potential evapotranspiration and hydrological fluxes respond primarily to climate variables. This structural inconsistency implies that projected PET and renewability rates reflect combined radiative and physiological forcing in some models, but radiative forcing only in others.

As such, we agree with the Reviewer that this difference in vegetation representation likely contributes to the large spread in projected PET across GWMs, even under identical GCM forcing. We have now included this aspect in the Discussion (lines 233-236), explicitly noting that heterogeneous treatment of CO_2 physiological effects across GWMs may represent an additional source of uncertainty in our analyses:

"Specifically, this study highlighted the different roles that variability and uncertainties in GCMs and GWMs play for water use and sustainability estimates (here quantified using WF and WD indicators), as we move from current conditions into future scenarios. Uncertainty in crop water use estimates (here embedded in WF) is dominated by the choice of GWMs, which provide divergent estimates of PET in terms of both global average annual values and their spatial variability (see Figs. S11--S12). Conversely, while the four climate models considered here show broad agreement in precipitation estimates (both annual totals and their distributions, Fig. S10), their discrepancies become increasingly prominent in future projections -- especially under high-emission scenarios -- thereby constraining the reliability of water use and sustainability projections. In deriving WD, defined as the ratio of WF to the locally available water resources (local renewability rates), these uncertainties are substantially magnified, reflecting the considerable divergence in renewability projections across GWMs.

Such variability reflects structural differences among global climate and water models, largely stemming from different model structures (e.g., Muller et al., 2014; Miralles et al., 2016; Burek et al., 2019; Reinecke et al., 2025). In particular, the representation of CO_2 physiological effects on vegetation (such as stomatal conductance and evapotranspiration), is not consistent across the GWMs considered here (Haddeland et al., 2011; Prudhomme et al., 2014; Reinecke et al., 2021) -- an aspect that may further contribute to the spread in projected PET and renewability estimates. The significant variability observed -- especially across GWMs -- highlights the

necessity of adopting ensemble approaches for crop water use and sustainability assessments, particularly in regions and at scales where discrepancies in precipitation, PET, and renewability rate estimates are largest."

15. Methods: It is hard to figure out at many points in the methods which data are being used and for what. It is common to group the description of all primary data inputs as a section called "data" at the very beginning of the methods. Generally the methods require clarification throughout.

Reply: We thank the Reviewer for this suggestion, with which we agree. We have now entirely revised the Methods section (lines 299-401). Specifically, we have: (i) added the subsection "Data", including a complete and detailed description of all data used in the study; (ii) amended the subsection "Crop water modeling" (previously called "Crop water modeling and crop-specific data") to focus solely on crop water modeling aspects (avoiding repetition on data-related aspects) and to include further details regarding the model (see also points 16 and 17 below); and (iii) removed the "Data from Global Water and Climate Models" section, consistently with the changes above. We believe that this revision has substantially improved the clarity of the Methods, making them more straightforward and easier to follow.

16. L251-253: Much more detail is required on how ks is calculated from the precipitation inputs from GCMs. This parameter is a key pivot point between GCM and GWM inputs, so it's essential to explain how it is calculated. I did not find the necessary details in the cited articles. Even if that information were clearly presented there, it should be summarized here in sufficient detail that readers can evaluate whether it is reasonable and what assumptions are embedded. It's also worth noting that the GCMs (and presumably the GWMs) also simulate soil moisture, so worth clarifying why you don't use those data directly.

Reply: Thank you for this suggestion - we agree with the Reviewer that the Methods would benefit from a more in-depth description of the ks calculation. We have now expanded the Methods to include more details on this (lines 417-430).
Regarding the last issue raised by the Reviewer, the soil moisture estimates from GWMs represent the fraction of precipitation, net of surface runoff and groundwater recharge, that infiltrates into the upper soil layer and recharges the soil water storage, becoming available for root water uptake and evapotranspiration (Tuninetti et al., 2019). As such, adopting estimates from GWMs in our framework would prevent our ability to estimate evapotranspiration for each crop under different conditions, which is key to the analysis.

17. L261: I think the renewability rates ultimately come from WaterGAP – summarize the data and justify why they are appropriate as applied here. This poses a bit of a problem here, because R comes from one of the models in the ensemble being evaluated, and is being applied to all of them. Presumably each model has its own representation of the relevant surface, soil and groundwater processes. So, using R from a single model probably underestimates the underlying model spread.

Reply: We thank the Reviewer for raising this issue. We would like to clarify that the renewability rates used in our analysis do not originate from a single model (e.g., WaterGAP). Instead, runoff ("qr"), groundwater recharge ("qs"), and root-zone soil moisture ("rootmoist") were obtained from the ISIMIP2b outputs of each individual global water model included in the ensemble (CWatM, PCR-GLOBWB, LPJmL, H08, WaterGAP2, and MATSIRO). Consequently,

the renewability rates are model-specific and reflect each model's internal representation of surface and subsurface processes. Within the ISIMIP2b framework, these variables are produced following harmonized definitions, but their computation remains consistent with the structure of each model. For example, groundwater recharge may correspond to percolation into an explicit groundwater storage compartment in some models, while in others it reflects deep drainage or soil-moisture fluxes; similarly, runoff generation mechanisms and root-zone soil moisture dynamics differ across models (Telteu et al., 2021). Therefore, the renewability rates used here inherently capture inter-model differences in hydrological process representation and do not impose a common WaterGAP-based constraint.

As this aspect may not have been sufficiently clear in the original manuscript and to avoid misinterpretations, we have now explicitly stated that renewability rates are considered separately for each model and have provided an additional reference to the ISIMIP protocol and the model intercomparison overview describing model differences (Telteu et al., 2021). The revised section now reads (lines 460-464):

"[...] where Y is the annual crop yield (ton ha^{-1}), Pr the annual crop production (ton), obtained as the product between yield and harvested area, ET the crop evapotranspiration during the growing period, A the grid cell area (m^2), and R the annual renewability rate of the water source (m yr^{-1}). Renewability rates include runoff, groundwater recharge, and root-zone soil moisture, and were derived from different GWMs within ISIMIP2b (see Data section), reflecting variability across models in the internal representation of hydrological processes (Telteu et al., 2021). To compute WD_b, we considered a blue water renewability rate (R_b) defined as the sum of runoff and groundwater recharge, whereas WD_g was calculated using green water renewability (R_g) represented by root-zone soil moisture. [...]"

18. L270: I agree these values for popcorn make no sense – probably worth tracing the error and alerting the dataset manager, as it may signal quality control issues. The use of the ÷ symbol here is nonstandard, consider – or ~ or the word 'to'

Reply: Thank you for noticing this, we changed the symbol with "–" accordingly.

19. L308-318: It generally is not very meaningful to sum ranges of 2 variables as an estimate of multivariate uncertainty. First, it ignores dependence structure between the two variables, and ignores all other data points than the max and min. Second, the range is not firmly grounded in statistical/probability theory, whereas variance is (and you are hinting at probabilistic projections in this paper). Third, summing ranges is inconsistent with error propagation theory. The standard approach would be to use variance and consider the total variance as σtotal2=σx2+σy2+2Cov(x,y). If you have a good reason not to use this more formally correct approach, it should be stated.

Reply: We thank the Reviewer for raising this relevant point. We acknowledge that summing ranges of two variables is not statistically rigorous, as it neglects both the dependence structure between sources of uncertainty and the limitations of the max-min range as a variability metric. As also highlighted by Reviewer #1 (comments 1 and 2), this formulation may lead to a misrepresentation of the total uncertainty. Following both Reviewers' advice, we have substantially revised the uncertainty framework throughout the manuscript.

First, we replaced the max-min range with the standard deviation (σ) as the primary uncertainty metric. Compared to the max-min range, σ provides a more robust and stable characterization of ensemble spread. We have supported this choice by performing a comparative analysis including also the interquartile range and max-min, now included in the Supplementary Information (**Fig. R12**), as well as by a leave-one-out sensitivity test demonstrating the robustness of the estimates. The revised Methods section now reads (lines 478-485):

*"**Methods***

[...]

Uncertainty analysis

We adopted the standard deviation as the primary uncertainty metric, as it provides a robust (Fig. S17) and balanced characterization of the ensemble spread. Alternative measures, including the interquartile range (IR) and the maximum-minimum range, were also evaluated (Fig. S18). While the IR reduces the influence of extreme values and thus yields a slightly more conservative estimate of variability (Prudhomme et al., 2014), the maximum-minimum range is highly sensitive to individual outlier model estimates. In comparison, the standard deviation retains sensitivity to variability across all models without being disproportionately influenced by single extreme realizations, thereby offering a suitable compromise for representing uncertainty in this study. [...]."

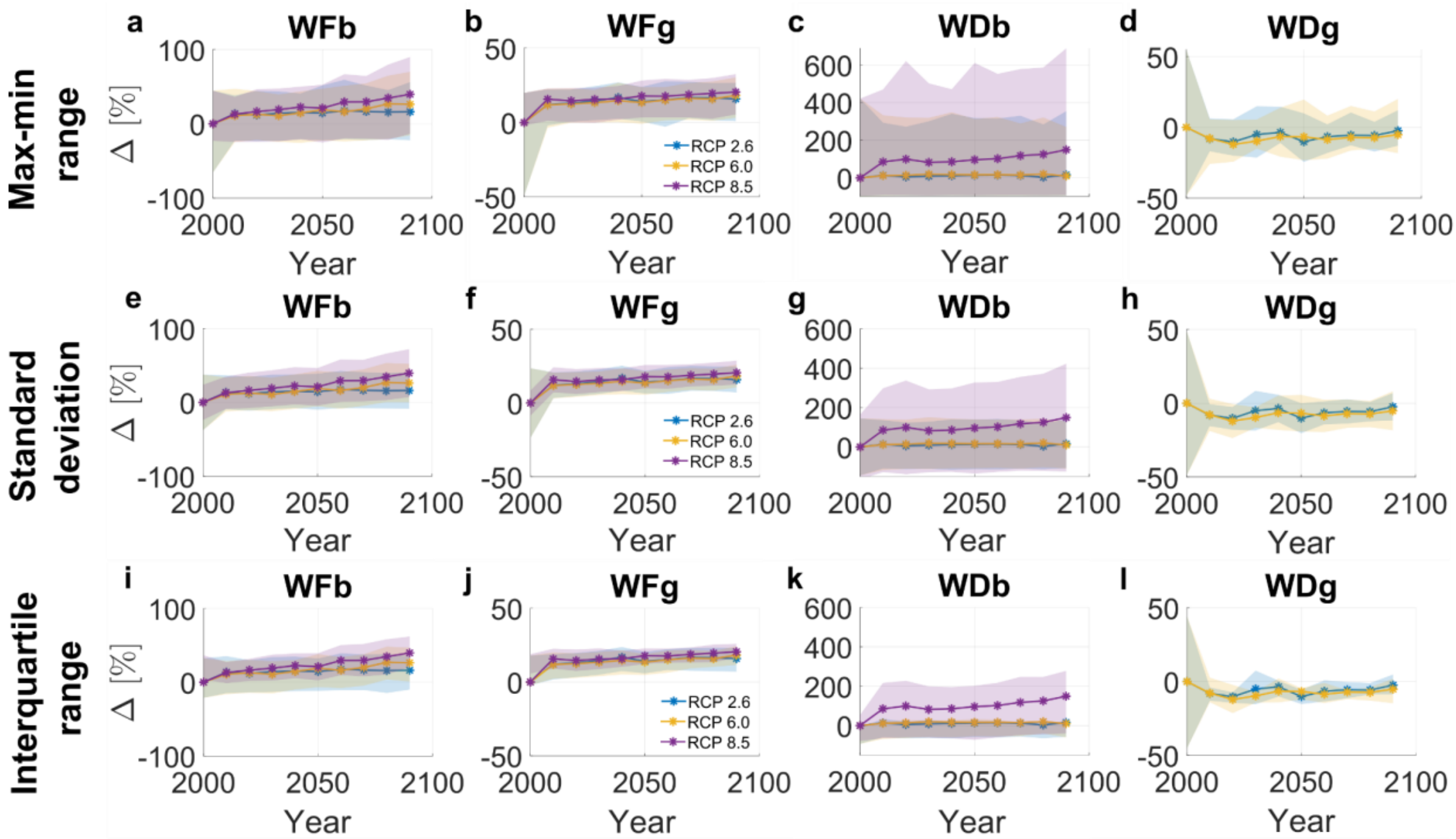

Figure R12 (new supplementary Fig. S18 in the revised manuscript): Comparative assessment of the variability in projections of global water footprint and water debt estimates using different metrics. Temporal evolution of the percentage change (Δ) (relative to the average in the reference year 2000) of global blue water footprint (WFb), green water footprint (WFg), blue water debt (WDb), and green water debt (WDg) - accounting for all the 8 crops. Solid lines represent average values across all GWMs and GCMs under RCP 2.6 (blue), 6.0 (yellow), and 8.5 (purple). The uncertainty ranges denoting variability among values from all models (colored shaded areas) are computed as: the maximum-minimum difference (panels **a-d**, equal to the original version of Fig. 1), the standard deviation (panels **e-h**, equal to the new Fig. 1 in the revised manuscript), and the interquartile range (panels **i-l**).

Second, we revised the definition of total uncertainty by adopting the coefficient of variation (CV), defined as (lines 486-488):

*"**Methods***

[...]

Uncertainty analysis

[...] To quantify the magnitude of uncertainty, we adopted the coefficient of variation (CV), defined as the standard deviation (σ) across all model estimates normalized by the multi-model ensemble mean:

$$CV = \frac{\sigma(V)}{\underline{V}}$$

where V denotes the considered variable (here, WF or WD) in each grid cell.

[...]".

This metric directly captures the total ensemble variability without relying on linear additivity assumptions or error propagation approximations. The previously used fractional uncertainty formulation has been discarded for consistency.

Third, to explicitly address the dependence between GCM and GWM contributions, we adopted a two-factor ANOVA variance decomposition (Montgomery, 2017). In our fully crossed ensemble (6 GWMs x 4 GCMs), each water model is forced by each climate model, resulting in a factorial design. In this context, the quantities previously denoted as GCM (C) and GWM (W) uncertainties correspond to variances of conditional means, rather than paired realizations of jointly sampled random variables. Therefore, a covariance term of the form Cov(W,C), as in classical error propagation, is not statistically defined. Instead, potential dependence between GCMs and GWMs manifests as interaction effects, which we explicitly quantify through the ANOVA framework (lines 489-507 in the revised Methods). The total variance is thus decomposed into three components, namely (i) GWM-related variance (σ^2_W), (ii) GCM-related variance (σ^2_C), and (iii) a GCM x GWM interaction term (σ^2_I), providing a statistically consistent measure of cross-model dependence.

These revisions are reflected throughout the manuscript:

- Figure 1 has been updated adopting the standard deviation to express variability (i.e. equal to panels e-h of **Fig. R12**);
- Figure 2 (**Fig. R1** - replies to Reviewer #1 above) and Figure A3 (scale-dependency) have been updated using the coefficient of variation CV, although spatial patterns remain overall consistent with the non-revised results, particularly regarding the location of hotspots;
- Figure 4 (**Fig. R2** - replies to Reviewer #1 above) now includes the interaction term together with GCM and GWM contributions, providing a more complete variance decomposition;
- The Results (lines 127-162, 171-210) and Methods (lines 477-517) sections have been revised accordingly.

Overall, while the main conclusions of the original manuscript remain unchanged, we believe that this revised framework significantly improves the statistical rigor and interpretability of the uncertainty analysis.

20. Code availability: Statement says processing codes are on Zenodo, but I only see figure reproduction code included. Full code required to reproduce the results should be included. See guidelines in: https://www.pnas.org/doi/10.1073/pnas.2422157123

> **Reply**: Thank you for this comment. This was indeed our intention, and the Zenodo repository (available at this link https://zenodo.org/records/17698932) has been updated accordingly. It now provides the code necessary to reproduce the revised figures, along with the implementation of the crop soil water balance model.

REFERENCES

Aeschbach-Hertig, W., & Gleeson, T. (2012). Regional strategies for the accelerating global problem of groundwater depletion. *Nature Geoscience*, *5*(12), 853-861.

Allen, R. G., Pereira, L. S., Raes, D., & Smith, M. (1998). Crop evapotranspiration-Guidelines for computing crop water requirements-FAO Irrigation and drainage paper 56. *Fao, rome*, *300*(9), D05109.

Burek, P., Satoh, Y., Kahil, T., Tang, T., Greve, P., Smilovic, M., ... & Wada, Y. (2020). Development of the Community Water Model (CWatM v1. 04)–a high-resolution hydrological model for global and regional assessment of integrated water resources management. *Geoscientific Model Development*, *13*(7), 3267-3298.

Frieler, K., Lange, S., Piontek, F., Reyer, C. P., Schewe, J., Warszawski, L., ... & Yamagata, Y. (2017). Assessing the impacts of 1.5 C global warming–simulation protocol of the Inter-Sectoral Impact Model Intercomparison Project (ISIMIP2b). *Geoscientific Model Development*, *10*(12), 4321-4345.

Gleeson, T., Wada, Y., Bierkens, M. F., & Van Beek, L. P. (2012). Water balance of global aquifers revealed by groundwater footprint. *Nature*, *488*(7410), 197-200.

Haddeland, I., Clark, D. B., Franssen, W., Ludwig, F., Voß, F., Arnell, N. W., ... & Yeh, P. (2011). Multimodel estimate of the global terrestrial water balance: setup and first results. *Journal of Hydrometeorology*, *12*(5), 869-884.

Hoekstra, A. Y., & Chapagain, A. K. (2007). Water footprints of nations: water use by people as a function of their consumption pattern. *Water resources management*, *21*(1), 35-48.

Hoekstra, A. Y., & Chapagain, A. K. (2011). *Globalization of water: Sharing the planet's freshwater resources*. John Wiley & Sons.

Lehner, F., Deser, C., Maher, N., Marotzke, J., Fischer, E. M., Brunner, L., ... & Hawkins, E. (2020). Partitioning climate projection uncertainty with multiple large ensembles and CMIP5/6. *Earth System Dynamics*, *11*(2), 491-508.

Lesk, C. S., Winter, J. M., & Mankin, J. S. (2025). Projected runoff declines from plant physiological effects on precipitation. *Nature Water*, *3*(2), 167-177.

Mekonnen, M. M., & Hoekstra, A. Y. (2011). The green, blue and grey water footprint of crops and derived crop products. *Hydrology and earth system sciences*, *15*(5), 1577-1600.

Mialyk, O., Schyns, J. F., Booij, M. J., Su, H., Hogeboom, R. J., & Berger, M. (2024). Water footprints and crop water use of 175 individual crops for 1990–2019 simulated with a global crop model. *Scientific data*, *11*(1), 206.

Minoli, S., Jägermeyr, J., Asseng, S., Urfels, A., & Müller, C. (2022). Global crop yields can be lifted by timely adaptation of growing periods to climate change. *Nature Communications*, *13*(1), 7079.

Miralles, D. G., Jiménez, C., Jung, M., Michel, D., Ershadi, A., McCabe, M. F., ... & Fernández-Prieto, D. (2016). The WACMOS-ET project–Part 2: Evaluation of global terrestrial evaporation data sets. *Hydrology and Earth System Sciences*, *20*(2), 823-842.

Montgomery, D. C. (2017). *Design and analysis of experiments*. John Wiley & Sons.

Müller Schmied, H., Eisner, S., Franz, D., Wattenbach, M., Portmann, F. T., Flörke, M., & Döll, P. (2014). Sensitivity of simulated global-scale freshwater fluxes and storages to input data, hydrological model structure, human water use and calibration. *Hydrology and Earth System Sciences*, *18*(9), 3511-3538.

Prudhomme, C., Giuntoli, I., Robinson, E. L., Clark, D. B., Arnell, N. W., Dankers, R., ... & Wisser, D. (2014). Hydrological droughts in the 21st century, hotspots and uncertainties from a global multimodel ensemble experiment. *Proceedings of the National Academy of Sciences*, *111*(9), 3262-3267.

Reinecke, R., Müller Schmied, H., Trautmann, T., Andersen, L. S., Burek, P., Flörke, M., ... & Döll, P. (2021). Uncertainty of simulated groundwater recharge at different global warming levels: a global-scale multi-model ensemble study. *Hydrology and Earth System Sciences*, *25*(2), 787-810.

Reinecke, R., Stein, L., Gnann, S., Andersson, J. C., Arheimer, B., Bierkens, M., ... & Wagener, T. (2025). Uncertainties as a guide for global water model advancement. *Wiley Interdisciplinary Reviews: Water*, *12*(3), e70025.

Schewe, J., Heinke, J., Gerten, D., Haddeland, I., Arnell, N. W., Clark, D. B., ... & Kabat, P. (2014). Multimodel assessment of water scarcity under climate change. *Proceedings of the National Academy of Sciences*, *111*(9), 3245-3250.

Telteu, C. E., Müller Schmied, H., Thiery, W., Leng, G., Burek, P., Liu, X., ... & Herz, F. (2021). Understanding each other's models: an introduction and a standard representation of 16 global water models to support intercomparison, improvement, and communication. *Geoscientific Model Development*, *14*(6), 3843-3878.

Tuninetti, M., Tamea, S., & Dalin, C. (2019). Water debt indicator reveals where agricultural water use exceeds sustainable levels. *Water Resources Research*, *55*(3), 2464-2477.

Walker, W. E., Harremoës, P., Rotmans, J., Van Der Sluijs, J. P., Van Asselt, M. B., Janssen, P., & Krayer von Krauss, M. P. (2003). Defining uncertainty: a conceptual basis for uncertainty management in model-based decision support. *Integrated assessment*, *4*(1), 5-17.

Zarei, M., & Destouni, G. (2024). A global multi catchment and multi dataset synthesis for water fluxes and storage changes on land. *Scientific Data*, *11*(1), 1333.

Zhang, J., & Liu, Y. (2022). Decoupling of impact factors reveals the response of cash crops phenology to climate change and adaptive management practice. *Agricultural and Forest Meteorology*, *322*, 109010.

Zhuo, L., Mekonnen, M. M., & Hoekstra, A. Y. (2014). Sensitivity and uncertainty in crop water footprint accounting: a case study for the Yellow River basin. *Hydrology and earth system sciences*, *18*(6), 2219-2234.

REVISION NOTES, 2nd ROUND - **Manuscript *"Uncertainties in global hydrological and climate models challenge future estimates of crop water use and sustainability"* (manuscript number COMMSENV-25-6197A) by Sun et al.**

Reviewer #1

The authors have done a good job addressing the reviewers' concerns. The manuscript is now robust and provides important insights into the propagation of model uncertainties in global crop water assessments.

> **Reply**: We thank the Reviewer for their appreciation of our revisions. The Reviewer's constructive suggestions and comments significantly helped us improve the robustness and clarity of the manuscript.

The authors might consider adding a brief concluding sentence that explicitly links the high inter-model uncertainty in these areas to specific local water management challenges, thereby further enhancing the policy relevance of this work.

> **Reply**: Thank you for this suggestion. We have added the following concluding remark at lines 283-286:
>
> *"[...] As such, a coral computational effort aiming at evaluating crop water needs and their sustainability based on different agro-hydrological model formulations [54–57] will be of utmost importance to further distill model (dis)agreements across space and time, particularly in major agricultural hotspots where large inter-model uncertainty complicates the design of robust local water management strategies. Ultimately, recognizing and transparently communicating uncertainty does not weaken water sustainability assessments; rather, it strengthens their credibility and reliability in guiding adaptive strategies under climate change."*

—

Reviewer #2

Thanks to authors for the thorough revisions. The new uncertainty framework is more solid, yet the conclusions are consistent with the original framework. I am also reassured that the uneven ensemble of GCMs and GWMs probably doesn't influence the results that much. There are many remaining limitations that cannot be addressed within the structure of the ISIMIP protocol employed here – these are issues to large to solve here, and I think the authors now do a sufficient job describing these limitations.

> **Reply**: We thank the Reviewer for the positive assessment of our revision work and agree that the framework is now more solid than the original version. We believe our manuscript has greatly benefited from the insightful Reviewer's comments.

Only a couple small adjustments to recommend, for clarity:

Fig. S9 – Including only the R2 for bin means inflates the strength of the dependence – I'd favour the lower R2 for the underlying data as the annotation on the panel, or you can include both.

Reply: We agree with the Reviewer's point. We have revised this supplementary Figure (see Figure R1 below) by removing the black dots indicating the mean of each bin (which could be misleading) and corrected R^2 so that it now refers to the total data rather than the bin means.

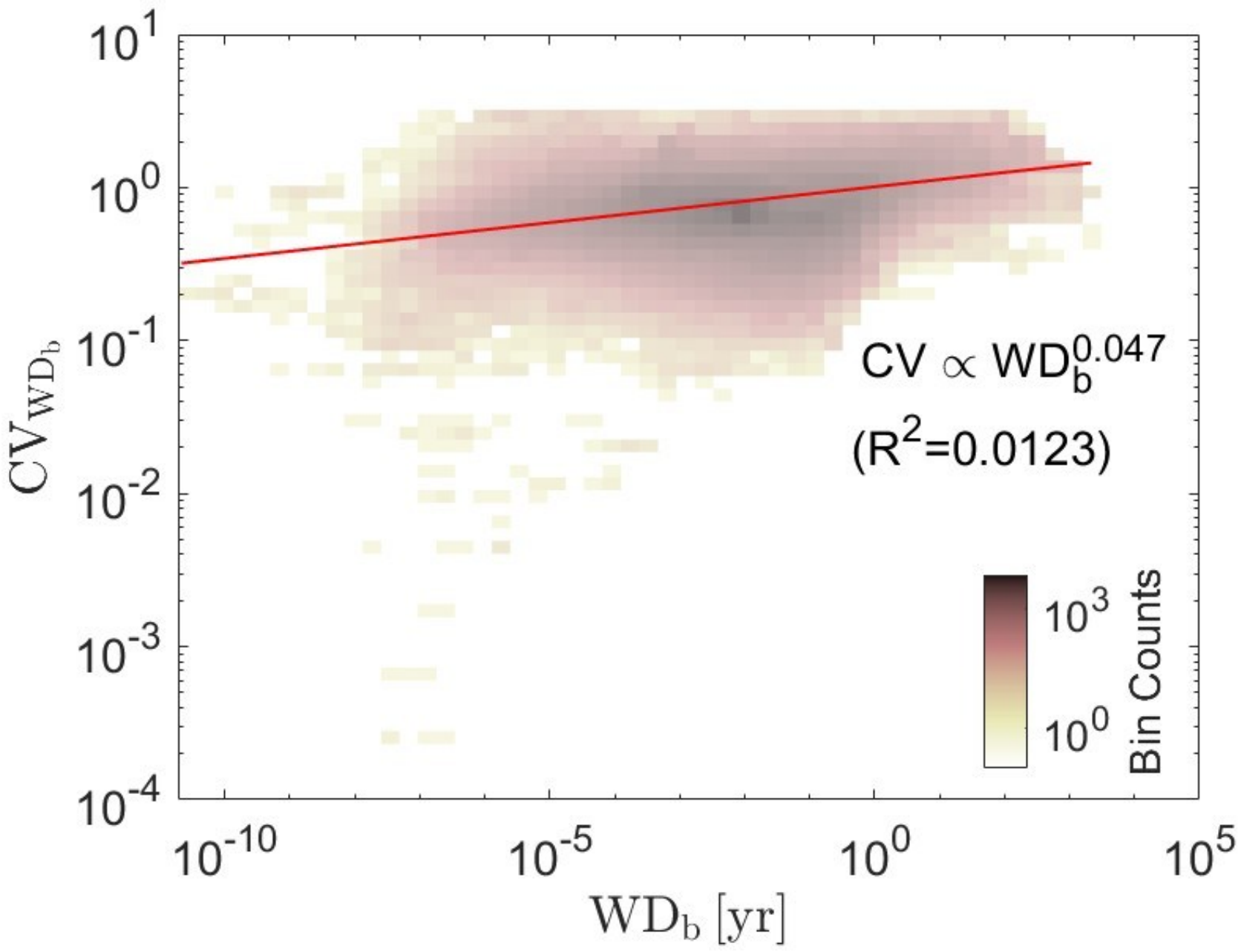

Figure R1 (same as new supplementary Fig. S12 in the manuscript): Bi-logarithmic grid-cell relationship between the blue water debt (WDb) and its associated coefficient of variation (CV), binned to increase readability (colors code the number of data points in each bin). The red line represents the linear regression (R^2≅0.0123) fitting the data.

We have also removed the specification from the main text accordingly (lines 148-149):

"A closer look at the spatial distribution of projected WDb [...]. Significant hotspots, for instance, are observed in the Central USA and along the Pakistan-India border, where the dominant crops in each region contribute to exceeding sustainable levels (potatoes, barley, and maize in the former; wheat, rice, and sugarcane in the latter; see insets in Fig. 2c). The positive but relatively weak correlation between WDb and CV(WDb) (slope of the linear fitting < 0.05, ~~R^2≅0.77,~~ Supplementary Fig. S12~~9~~) reflects the fact that, while high water debt is associated with higher uncertainty, such conditions are confined to spatially limited hotspots."

L480 – I think you're referring to Fig. S18, not S17.

Reply: We see the Reviewer's doubt, but the reference to Supplementary Fig. S17 (now Fig. S15, due to a reordering of the supplementary figures to match the first time they are called in the text) is actually the correct one. Indeed, in the following paragraph (lines 410-412), we firstly refer to Fig. S15 (old Fig. S17) to denote the robustness of the standard deviation, and then we mention that other metrics were taken into consideration. Figure S15 (see Fig. R2 below) shows the limited sensitivity to the exclusion of individual models (leave-one-model-out analysis), stressing the fact that the overall uncertainty appears robust to the ensemble

composition. Instead, Figure S18 only shows the comparison between three different metrics, namely the maximum-minimum range, the standard deviation, and the interquartile range.

*“**Uncertainty analysis***

We adopted the standard deviation as the primary uncertainty metric, as it provides a robust (Supplementary Fig. S15~~7~~) and balanced characterization of the ensemble spread. Alternative measures, including the interquartile range (IR) and the maximum-minimum range, were also evaluated (Supplementary Fig. S18). While the IR reduces [...].”

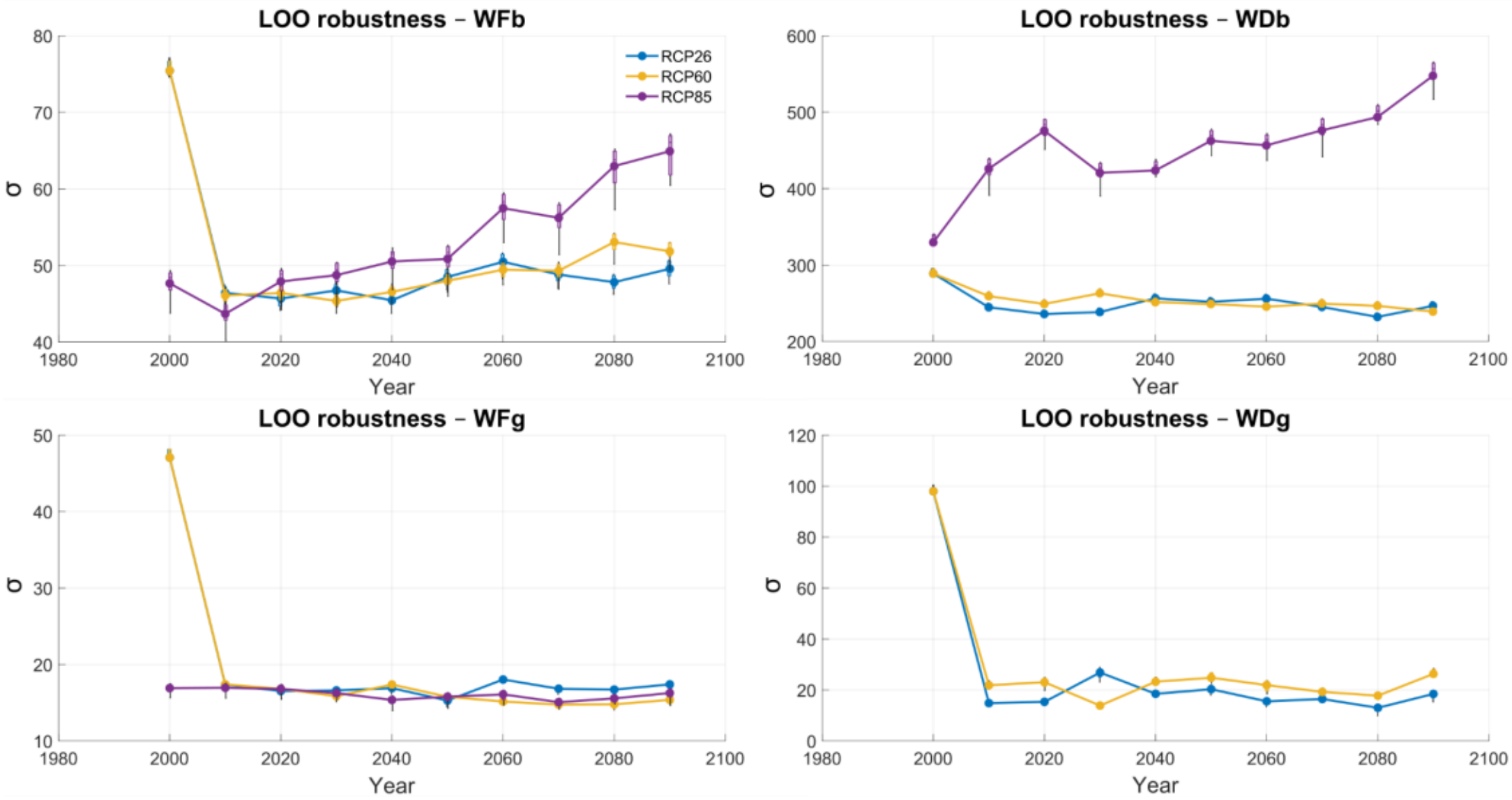

Figure R2 (same as supplementary Fig. S15 in the revised manuscript): Robustness of the uncertainty metric, namely the standard deviation relative to year 2000 (σ, %), assessed through a leave-one-out (LOO) analysis. Panels show results for WFb (top left), WFg (bottom left), WDb (top right), and WDg (bottom right) WDg, under RCP 2.6 (blue), 6.0 (yellow), and 8.5 (purple). The continuous line represents the standard deviation computed using the full ensemble of models, while the error bars depict the distribution of uncertainty estimates obtained by sequentially excluding one model at a time. The limited spread of the boxplots relative to the reference line indicates that the estimated uncertainty remains stable when individual models are omitted. The maximum variability is found for WFb in the year 2090 under RCP 8.5 (median 66.06%, quartiles 61.82% and 66.92%).
