# Supplementary material for: Uncertainties in global hydrological and climate models challenge future estimates of crop water use and sustainability
Source: Commun Earth Environ. 2026 May 26;7(1):676. doi: 10.1038/s43247-026-03621-w (PMC13485690; doi:10.1038/s43247-026-03621-w)
Supplement: Supplementary file 2 [file 43247_2026_3621_MOESM2_ESM.pdf]

Supplementary Material for Uncertainties in global hydrological and climate models challenge future estimates of crop water use and sustainability

Qiming Sun[1†], Francesca Bassani [1†], Marta Tuninetti[2], Sara Bonetti[1*]

[1*]Laboratory of Catchment Hydrology and Geomorphology, École Polytechnique Fédérale de Lausanne (EPFL), Route des Ronquos 86, Sion, 1951, Switzerland.
[2]Department of Environment, Land and Infrastructure Engineering, Politecnico di Torino, Corso Duca degli Abruzzi 24, Torino, 10129, Italy.

*Corresponding author(s). E-mail(s): sara.bonetti@epfl.ch;
†These authors contributed equally to this work.

Supplementary Tables

Table S1: Annual global values of blue, green, and total water footprint (WF), blue water debt (WD_b), and green water debt (WD_g) for the reference year 2000. Values are reported as mean ± standard deviation across all CWM and GWM combinations.

Crop	Year 2000 (historical scenario)				
	WF_b $[m^3]$	WF_g $[m^3]$	$WF_b + WF_g$ $[m^3]$	WD_b $[yr]$	WD_g $[yr]$
Barley	$(5.8 \pm 3.3) \cdot 10^9$	$(1.5 \pm 0.4) \cdot 10^{11}$	$(1.6 \pm 0.4) \cdot 10^{11}$	0.013 ± 0.013	0.002 ± 0.0008
Maize	$(4.4 \pm 2.9) \cdot 10^{10}$	$(6.3 \pm 1.5) \cdot 10^{11}$	$(6.8 \pm 1.8) \cdot 10^{11}$	0.118 ± 0.169	0.006 ± 0.003
Potato	$(1.0 \pm 0.5) \cdot 10^{10}$	$(6.6 \pm 1.3) \cdot 10^{10}$	$(7.7 \pm 1.8) \cdot 10^{10}$	0.024 ± 0.019	0.0008 ± 0.0003
Rice	$(3.9 \pm 0.9) \cdot 10^{11}$	$(5.5 \pm 1.3) \cdot 10^{11}$	$(9.4 \pm 2.2) \cdot 10^{11}$	0.280 ± 0.480	0.008 ± 0.003
Sorghum	$(6.6 \pm 3.6) \cdot 10^9$	$(1.4 \pm 0.4) \cdot 10^{11}$	$(1.5 \pm 0.4) \cdot 10^{11}$	0.130 ± 0.116	0.004 ± 0.001
Soybean	$(8.7 \pm 6.6) \cdot 10^9$	$(3.7 \pm 0.8) \cdot 10^{11}$	$(3.7 \pm 0.9) \cdot 10^{11}$	0.002 ± 0.002	0.008 ± 0.004
Sugarcane	$(6.9 \pm 3.8) \cdot 10^{10}$	$(2.1 \pm 0.5) \cdot 10^{11}$	$(2.7 \pm 0.8) \cdot 10^{11}$	0.116 ± 0.119	0.109 ± 0.107
Wheat	$(1.1 \pm 0.7) \cdot 10^{11}$	$(7.4 \pm 1.9) \cdot 10^{11}$	$(8.5 \pm 2.6) \cdot 10^{11}$	0.214 ± 0.276	0.006 ± 0.002
Total	$(6.4 \pm 2.4) \cdot 10^{11}$	$(2.9 \pm 0.7) \cdot 10^{12}$	$(3.5 \pm 0.9) \cdot 10^{12}$	0.953 ± 1.376	0.021 ± 0.010

Table S2: Annual global values of blue, green, and total water footprint (WF), blue water debt (WD_b), and green water debt (WD_g) for the year 2090 (RCP 2.6). Values are reported as mean ± standard deviation across all GCM and GWM combinations..

Crop	Year 2090				
	RCP 2.6				
	WF_b $[m^3]$	WF_g $[m^3]$	$WF_b + WF_g$ $[m^3]$	WD_b $[yr]$	WD_g $[yr]$
Barley	$(8.0 \pm 2.3) \cdot 10^9$	$(1.8 \pm 0.2) \cdot 10^{11}$	$(1.8 \pm 0.2) \cdot 10^{11}$	0.018 ± 0.015	0.002 ± 0.0008
Maize	$(5.8 \pm 2.2) \cdot 10^{10}$	$(7.2 \pm 0.6) \cdot 10^{11}$	$(7.8 \pm 0.8) \cdot 10^{11}$	0.12 ± 0.131	0.007 ± 0.003
Potato	$(1.3 \pm 0.3) \cdot 10^{10}$	$(7.6 \pm 0.4) \cdot 10^{10}$	$(8.8 \pm 0.7) \cdot 10^{10}$	0.037 ± 0.028	0.0009 ± 0.0003
Rice	$(4.2 \pm 0.6) \cdot 10^{11}$	$(6.6 \pm 0.5) \cdot 10^{11}$	$(1.1 \pm 0.1) \cdot 10^{12}$	0.309 ± 0.492	0.009 ± 0.003
Sorghum	$(7.9 \pm 2.0) \cdot 10^9$	$(1.6 \pm 0.1) \cdot 10^{11}$	$(1.7 \pm 0.1) \cdot 10^{11}$	0.173 ± 0.108	0.005 ± 0.002
Soybean	$(1.0 \pm 0.5) \cdot 10^{10}$	$(4.1 \pm 0.3) \cdot 10^{11}$	$(4.2 \pm 0.4) \cdot 10^{11}$	0.002 ± 0.002	0.009 ± 0.004
Sugarcane	$(7.8 \pm 2.5) \cdot 10^{10}$	$(2.4 \pm 0.2) \cdot 10^{11}$	$(3.2 \pm 0.5) \cdot 10^{11}$	0.150 ± 0.102	0.112 ± 0.109
Wheat	$(1.4 \pm 0.5) \cdot 10^{11}$	$(8.6 \pm 0.9) \cdot 10^{11}$	$(1.0 \pm 0.1) \cdot 10^{12}$	0.206 ± 0.185	0.007 ± 0.002
Total	$(7.4 \pm 1.6) \cdot 10^{11}$	$(3.3 \pm 0.2) \cdot 10^{12}$	$(4.1 \pm 0.4) \cdot 10^{12}$	1.105 ± 1.175	0.021 ± 0.002

Table S3: Annual global values of blue, green, and total water footprint (WF), blue water debt (WD_b), and green water debt (WD_g) for the year 2090 (RCP 6.0). Values are reported as mean ± standard deviation across all GCM and GWM combinations.

Crop	Year 2090				
	RCP 6.0				
	WF_b $[m^3]$	WF_g $[m^3]$	$WF_b + WF_g$ $[m^3]$	WD_b $[yr]$	WD_g $[yr]$
Barley	$(9.0 \pm 2.4) \cdot 10^9$	$(1.8 \pm 0.2) \cdot 10^{11}$	$(1.9 \pm 0.2) \cdot 10^{11}$	0.017 ± 0.013	0.002 ± 0.0006
Maize	$(6.8 \pm 2.5) \cdot 10^{10}$	$(7.4 \pm 0.6) \cdot 10^{11}$	$(8.1 \pm 0.8) \cdot 10^{11}$	0.128 ± 0.138	0.007 ± 0.003
Potato	$(1.4 \pm 0.3) \cdot 10^{10}$	$(7.7 \pm 0.5) \cdot 10^{10}$	$(9.1 \pm 0.9) \cdot 10^{10}$	0.040 ± 0.028	0.0009 ± 0.0003
Rice	$(4.5 \pm 0.6) \cdot 10^{11}$	$(6.7 \pm 0.4) \cdot 10^{11}$	$(1.1 \pm 0.1) \cdot 10^{12}$	0.314 ± 0.501	0.009 ± 0.003
Sorghum	$(9.2 \pm 2.1) \cdot 10^9$	$(1.6 \pm 0.1) \cdot 10^{11}$	$(1.7 \pm 0.1) \cdot 10^{11}$	0.173 ± 0.108	0.005 ± 0.002
Soybean	$(1.3 \pm 0.6) \cdot 10^{10}$	$(4.3 \pm 0.4) \cdot 10^{11}$	$(4.4 \pm 0.4) \cdot 10^{11}$	0.003 ± 0.002	0.009 ± 0.004
Sugarcane	$(8.6 \pm 2.5) \cdot 10^{10}$	$(2.5 \pm 0.2) \cdot 10^{11}$	$(3.4 \pm 0.5) \cdot 10^{11}$	0.130 ± 0.094	0.105 ± 0.104
Wheat	$(1.6 \pm 0.5) \cdot 10^{11}$	$(8.8 \pm 0.8) \cdot 10^{11}$	$(1.0 \pm 0.1) \cdot 10^{12}$	0.191 ± 0.171	0.007 ± 0.002
Total	$(8.1 \pm 1.7) \cdot 10^{11}$	$(3.4 \pm 0.2) \cdot 10^{12}$	$(4.2 \pm 0.4) \cdot 10^{12}$	1.051 ± 1.139	0.020 ± 0.003

Table S4: Annual global values of blue, green, and total water footprint (WF), blue water debt (WD_b), and green water debt (WD_g) for the year 2090 (RCP 8.5). Values are reported as mean ± standard deviation across all GCM and GWM combinations. The unavailability of the root zone soil moisture prevented the computation of WD_g.

Crop	Year 2090				
	RCP 8.5				
	WF_b $[m^3]$	WF_g $[m^3]$	$WF_b + WF_g$ $[m^3]$	WD_b $[yr]$	WD_g $[yr]$
Barley	$(9.5 \pm 2.9) \cdot 10^9$	$(1.8 \pm 0.2) \cdot 10^{11}$	$(1.9 \pm 0.2) \cdot 10^{11}$	0.019 ± 0.013	NA
Maize	$(7.5 \pm 3.1) \cdot 10^{10}$	$(7.4 \pm 0.5) \cdot 10^{11}$	$(8.1 \pm 0.8) \cdot 10^{11}$	0.134 ± 0.142	NA
Potato	$(1.5 \pm 0.4) \cdot 10^{10}$	$(7.6 \pm 0.5) \cdot 10^{10}$	$(9.1 \pm 1.0) \cdot 10^{10}$	0.045 ± 0.031	NA
Rice	$(4.6 \pm 0.7) \cdot 10^{11}$	$(6.8 \pm 0.5) \cdot 10^{11}$	$(1.1 \pm 0.1) \cdot 10^{12}$	0.351 ± 0.551	NA
Sorghum	$(1.0 \pm 0.3) \cdot 10^{10}$	$(1.7 \pm 0.1) \cdot 10^{11}$	$(1.8 \pm 0.1) \cdot 10^{11}$	0.211 ± 0.124	NA
Soybean	$(1.5 \pm 0.8) \cdot 10^{10}$	$(4.3 \pm 0.4) \cdot 10^{11}$	$(4.4 \pm 0.4) \cdot 10^{11}$	0.003 ± 0.002	NA
Sugarcane	$(9.0 \pm 2.6) \cdot 10^{10}$	$(2.5 \pm 0.2) \cdot 10^{11}$	$(3.4 \pm 0.4) \cdot 10^{11}$	0.163 ± 0.143	NA
Wheat	$(1.7 \pm 0.6) \cdot 10^{11}$	$(8.8 \pm 0.8) \cdot 10^{11}$	$(1.0 \pm 0.1) \cdot 10^{12}$	0.265 ± 0.290	NA
Total	$(8.9 \pm 2.1) \cdot 10^{11}$	$(3.4 \pm 0.2) \cdot 10^{12}$	$(4.3 \pm 0.4) \cdot 10^{12}$	2.375 ± 2.608	NA

Table S5: Summary of p-values from the Mann-Kendall trend test (the null hypothesis is trend absence [1, 2]) applied to the time series of global averages of blue and green water footprints (WF_b, WF_g), and blue and green water debts (WD_b, WD_g), corresponding to the solid lines shown in Fig. 1 of the main text. Values below 0.05 indicate a rejection of the null hypothesis.

	WF_b	WF_g	WD_b	WD_g
RCP 2.6	0.029	0.076	0.348	0.175
RCP 6.0	0.005	0.001	0.917	0.175
RCP 8.5	0.001	0.002	0.005	–

Table S6: Maximum root depth ($Z_{r,max}$) [3] and depletion fraction coefficient (ρ) [3] for the eight crops considered in this study.

Crop	$Z_{r,max}$ $[m]$	ρ
Barley	1.25	0.55
Maize	1.4	0.55
Potato	0.5	0.35
Rice	0.75	0.2
Sorghum	1.5	0.55
Soybean	0.95	0.5
Sugarcane	1.2	0.65
Wheat	1.5	0.55

Supplementary Figures

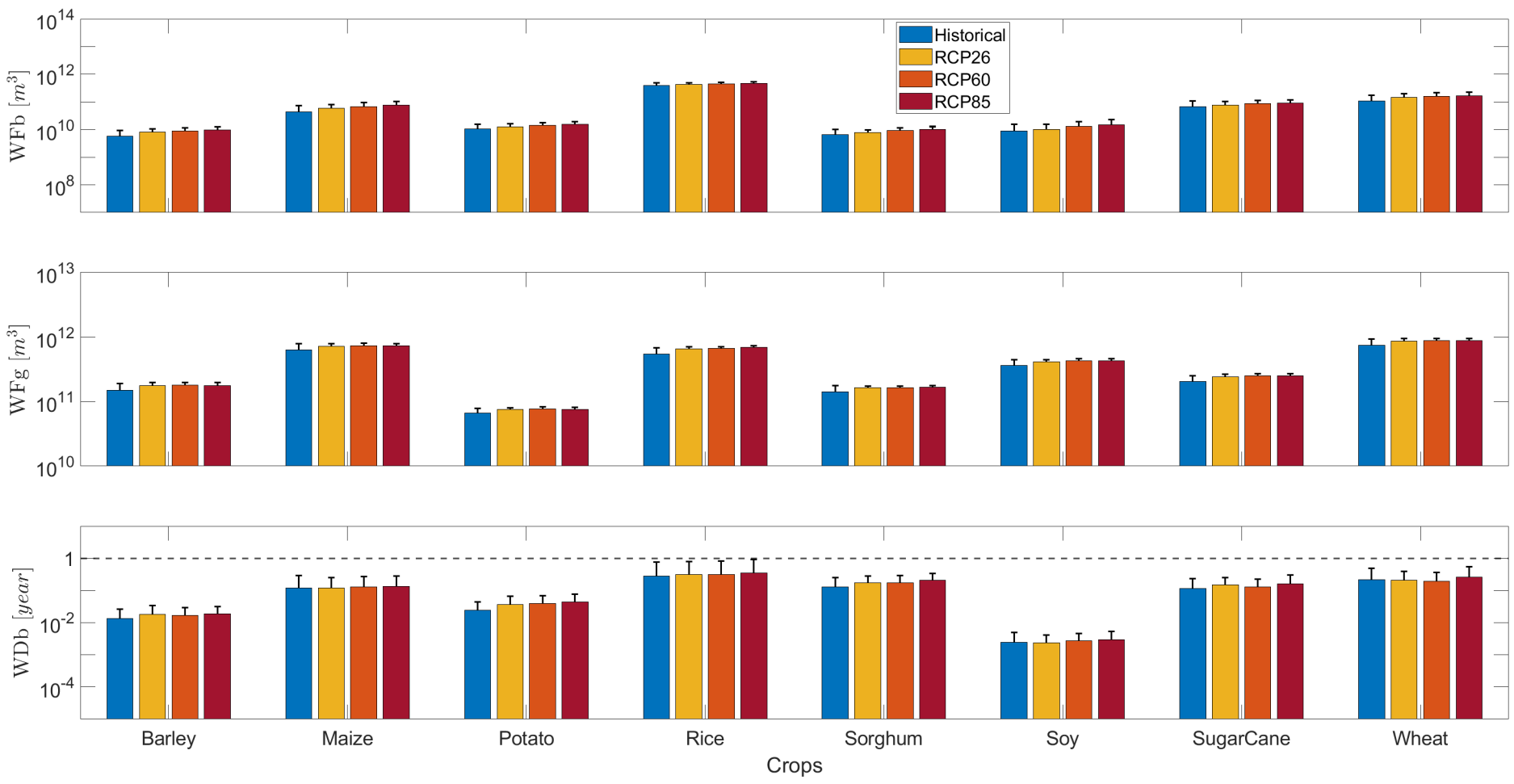

Fig. S1: Crop-specific global values of blue water footprint WF_b, green water footprint WF_g, blue water debt WD_b, computed for the reference year 2000 (blue) and future projections of year 2090 under RCP 2.6 (yellow), RCP 6.0 (orange), and RCP 8.5 (red). Error bars indicate the variability among all climate and water models. The dashed black line in the WD_b plot represents the sustainability threshold (WD_b= 1).

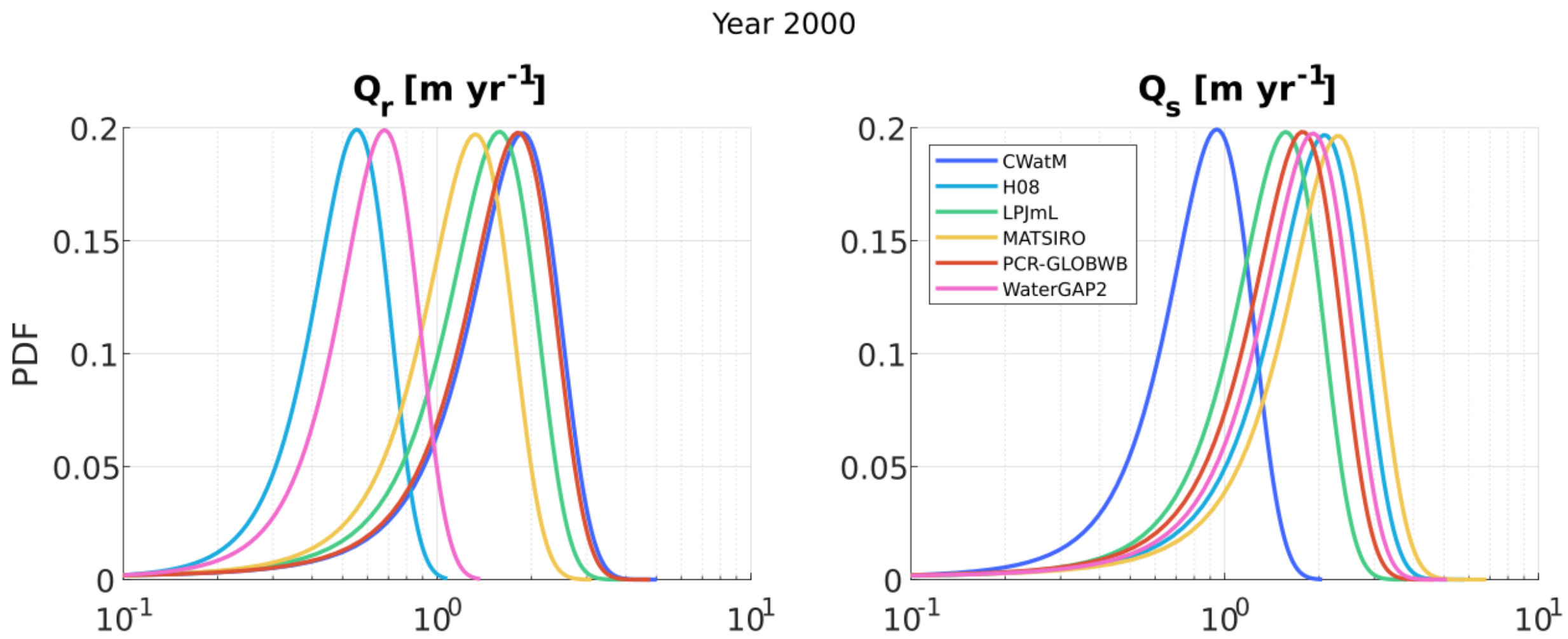

Fig. S2: Probability density functions (PDFs) of the blue renewability rates evaluated in each grid cell (left: surface runoff Q_r, right: groundwater recharge Q_s) for the 6 Water Models considered in the year 2000.

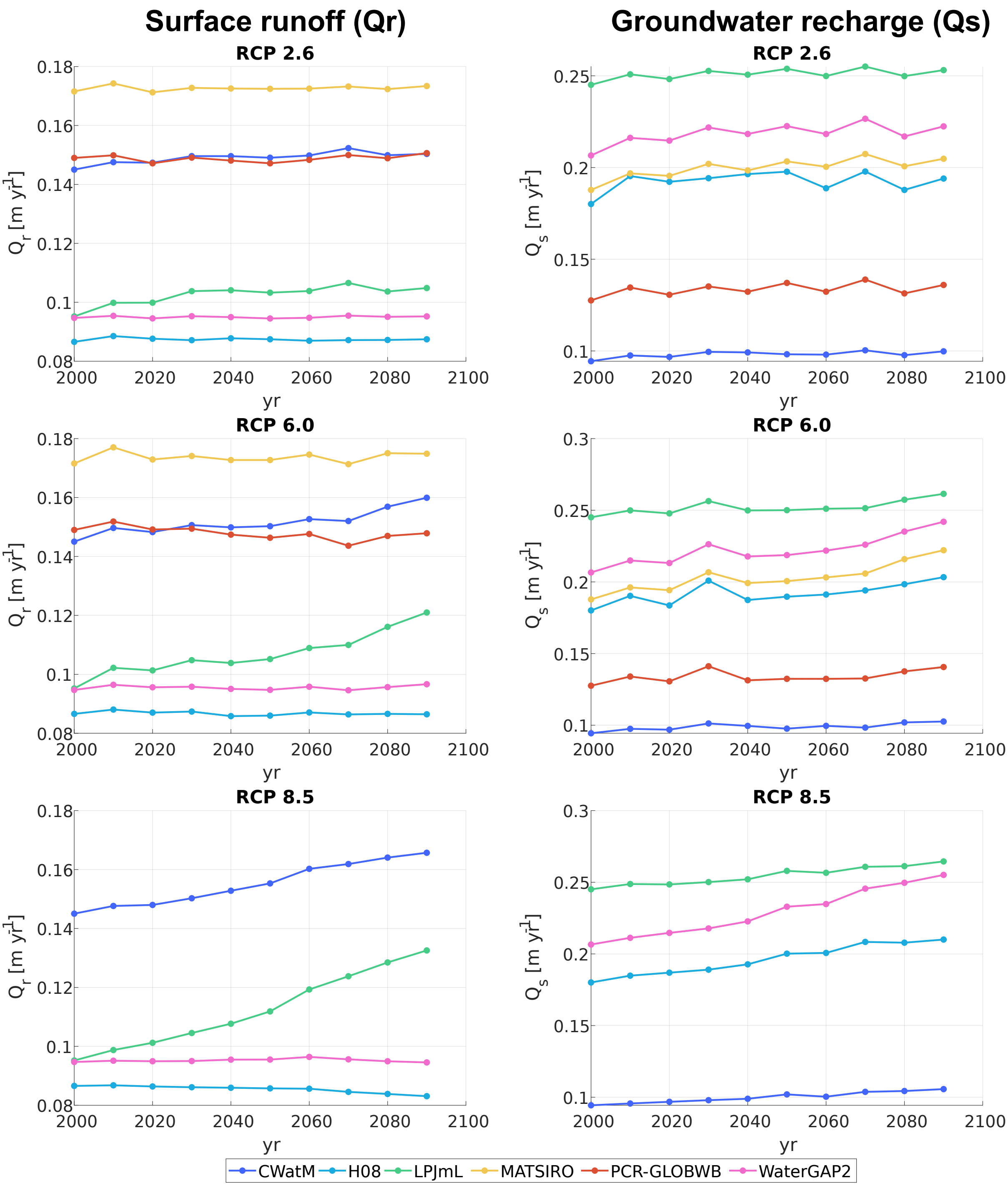

Fig. S3: Temporal variation of mean global annual blue renewability rates for the 6 Water Models considered and across the 10 years. Left and right columns refer to surface runoff (Q_r) and groundwater recharge (Q_s), respectively. Top, middle, and bottom panels refer to RCPs 2.6, 6.0 and 8.5, respectively. Note that Q_r and Q_s are not available for the models MATSIRO and PCR-GLOBWB under RCP 8.5 (Tab. 1 of the main text).

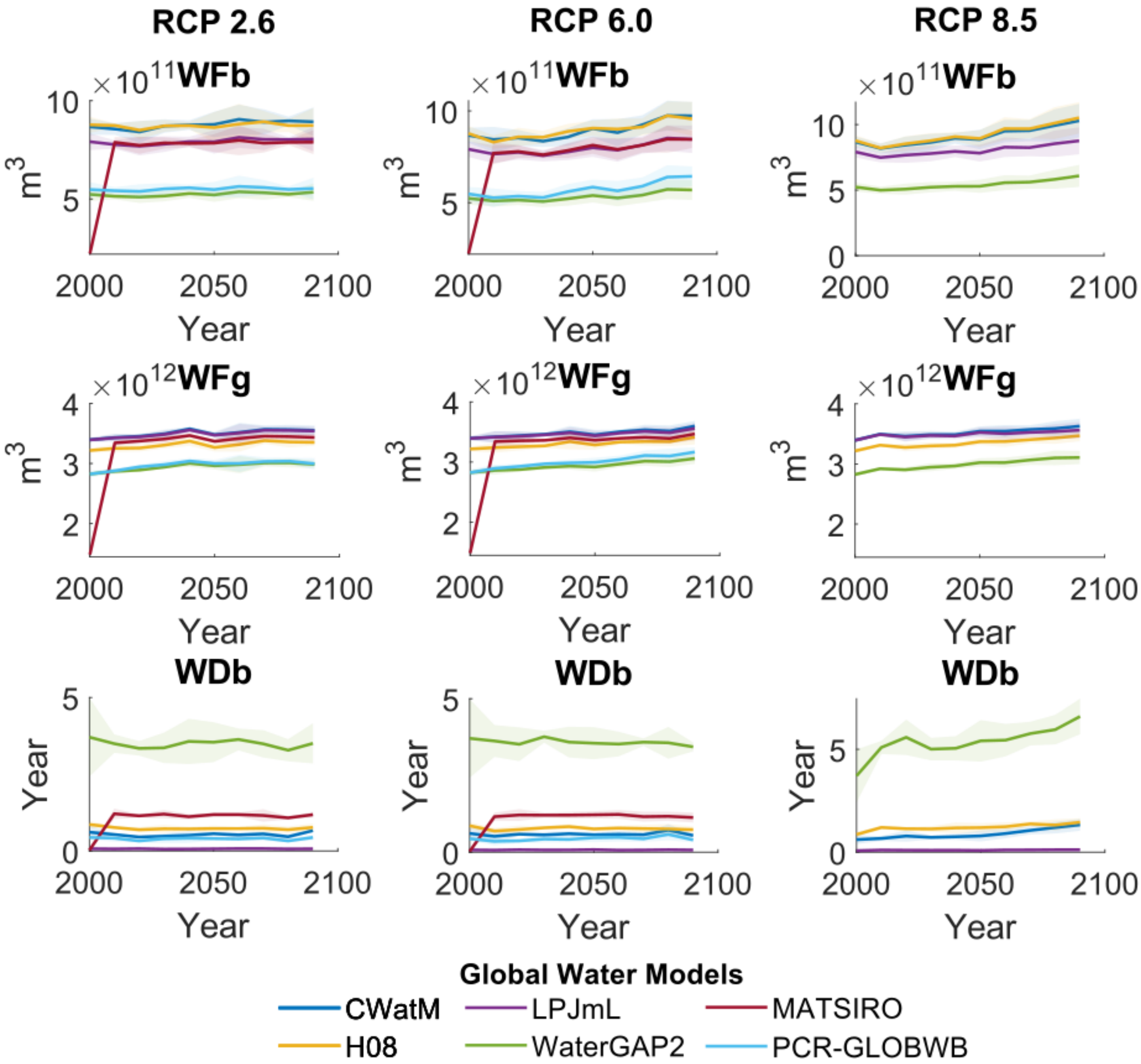

Fig. S4: Temporal evolution of global water footprint and water debt for different GWMs. Global blue water footprint WF_b, green water footprint WF_g, and blue water debt WD_b under the three RCPs, and for each of the six GWMs (averaged for the four GCMs), considering all crops. Shaded areas denote the standard deviation among all models. MATSIRO and PCR-GLOBWB models are not available for RCP 8.5.

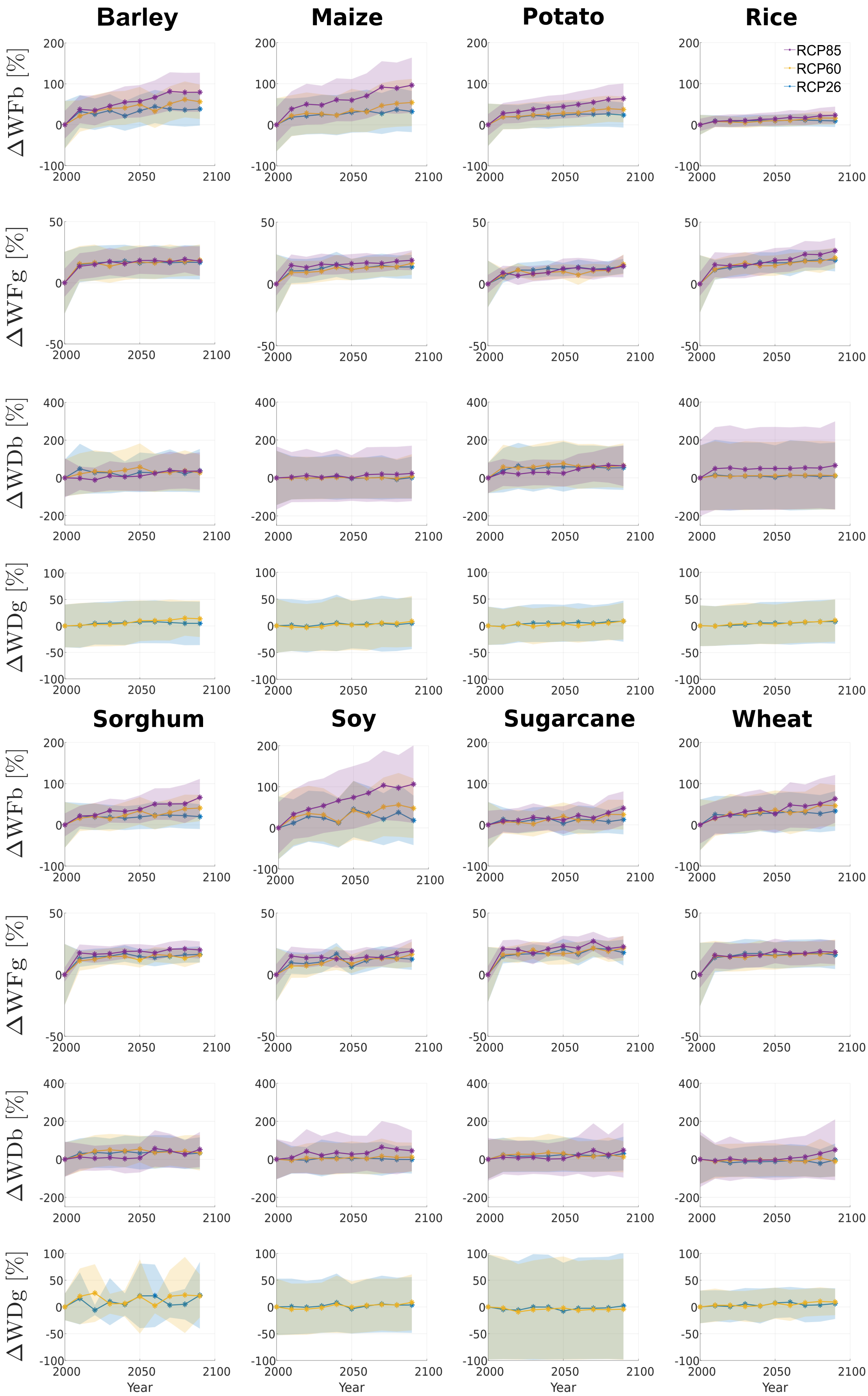

Fig. S5: Temporal evolution of the percentage change Δ (relative to the average in the reference year 2000) of global blue and green water footprints (WF_b and WF_g, respectively), and blue and green water debts (WD_b, WD_g) for all the 8 crops and the RCPs considered. Solid lines represent average values across all models (climate and water models) under RCP 2.6 (blue), 6.0 (yellow), and 8.5 (purple). Shaded areas denote the uncertainty computed as the standard deviation among values from all models. WD_g is not available under RCP 8.5.

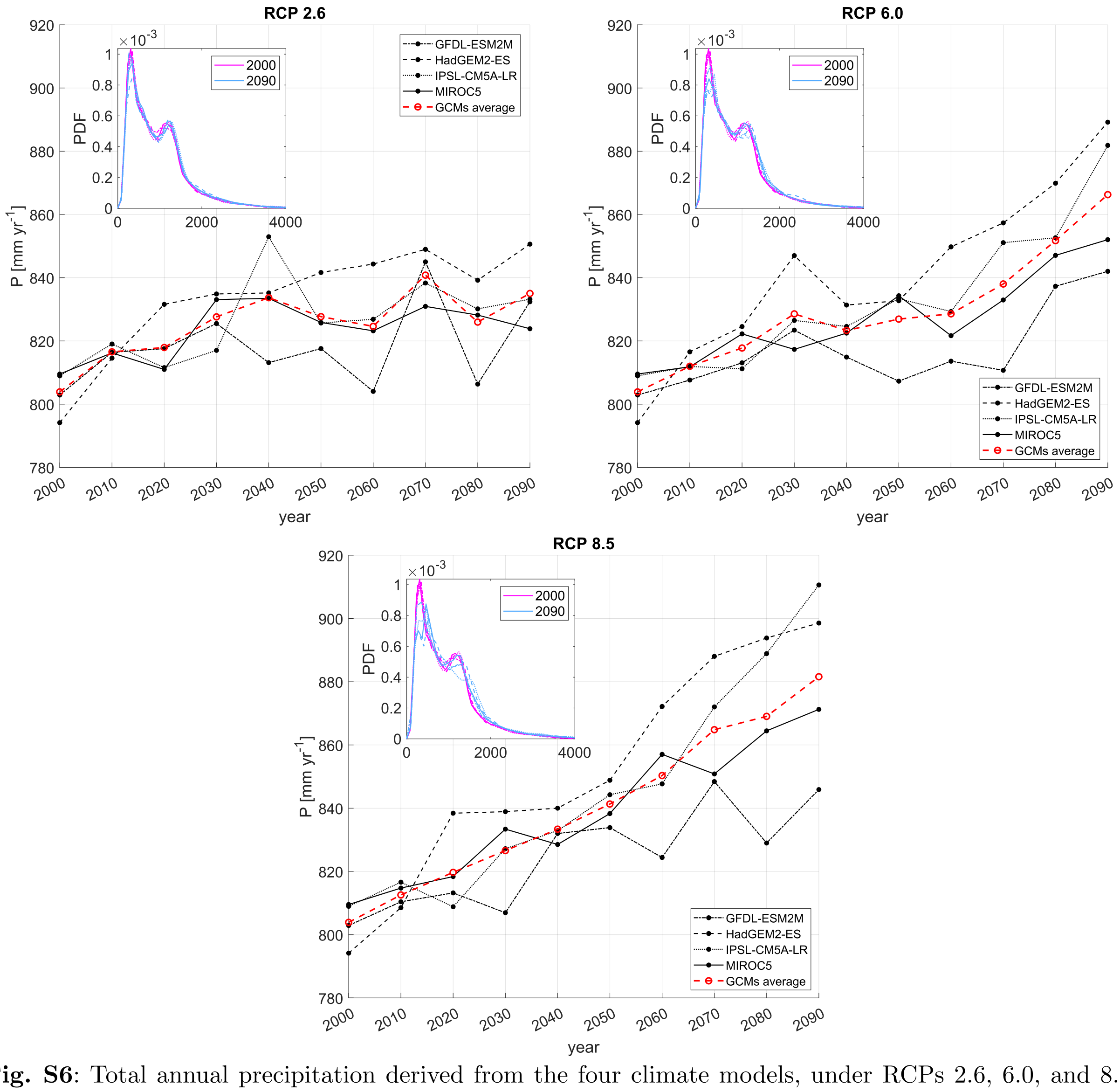

Fig. S6: Total annual precipitation derived from the four climate models, under RCPs 2.6, 6.0, and 8.5. Insets show the probability density function (PDF) of grid cell values for years 2000 and 2090.

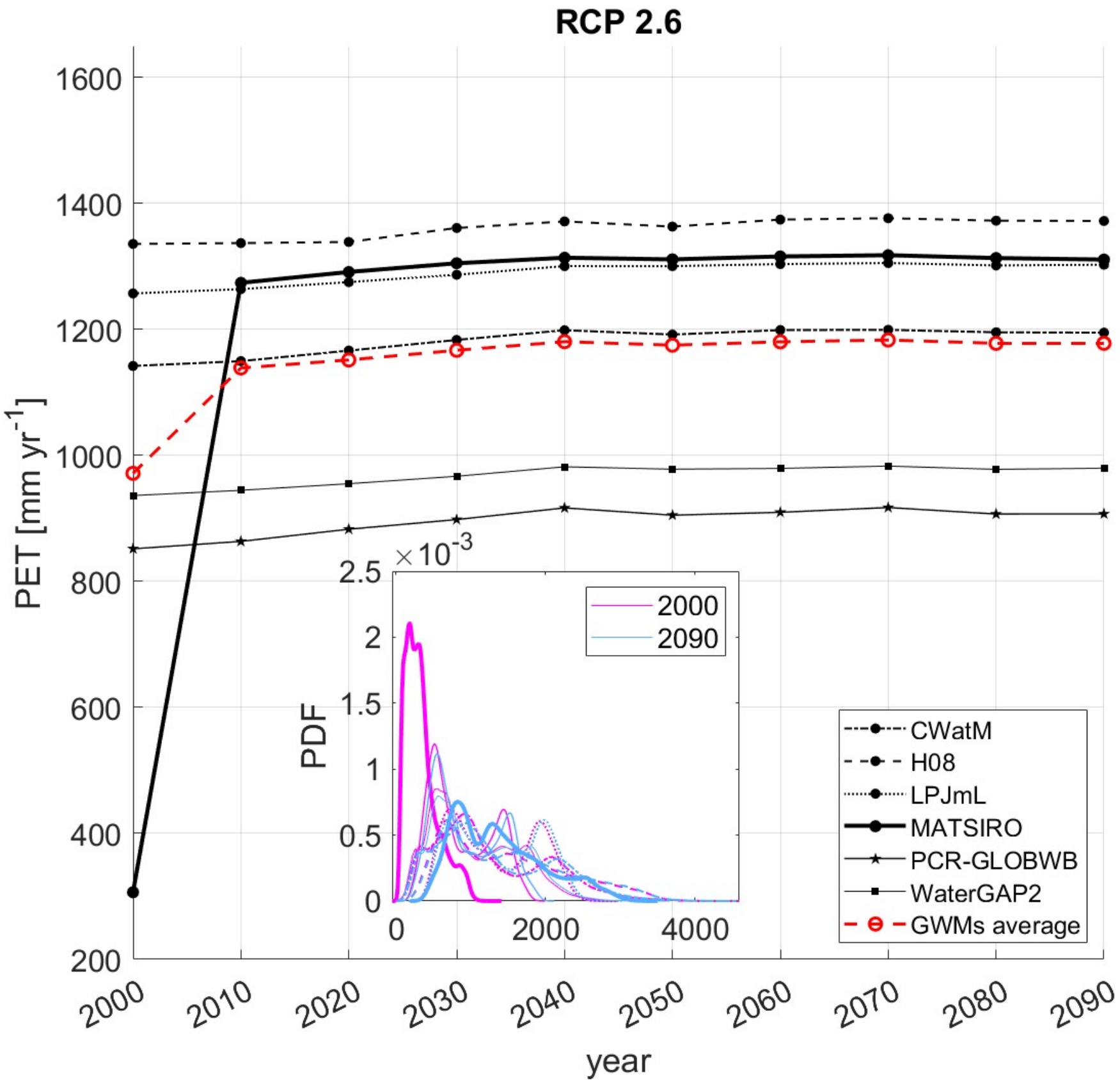

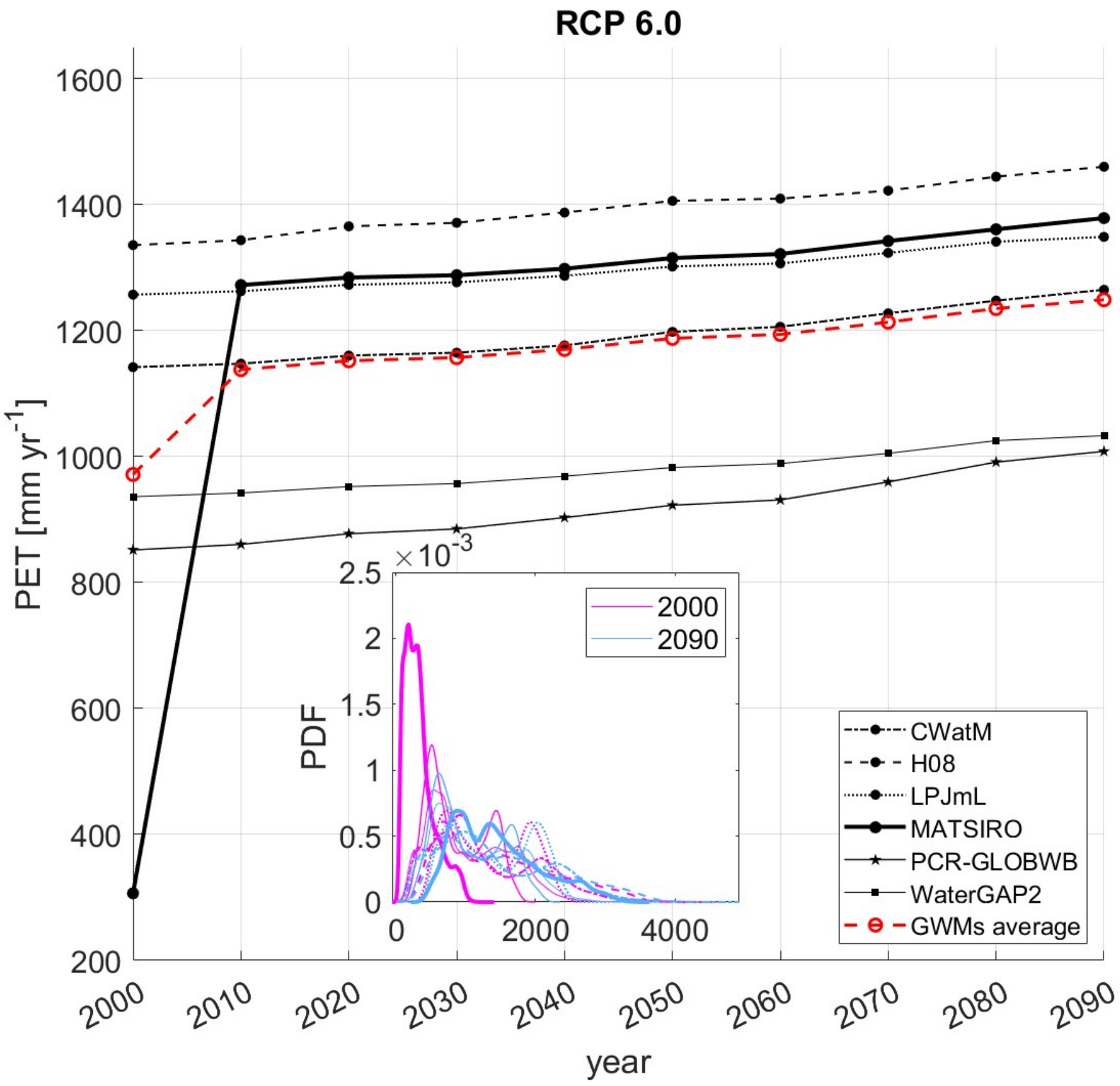

Fig. S7: Mean global total annual reference potential evapotranspiration (PET) derived from the six GWMs. Insets show the probability density functions (PDF) of annual PET values in each grid cell for years 2000 and 2090, under RCP 2.6 (upper panel) and RCP 6.0 (bottom panel) for the different GWMs.

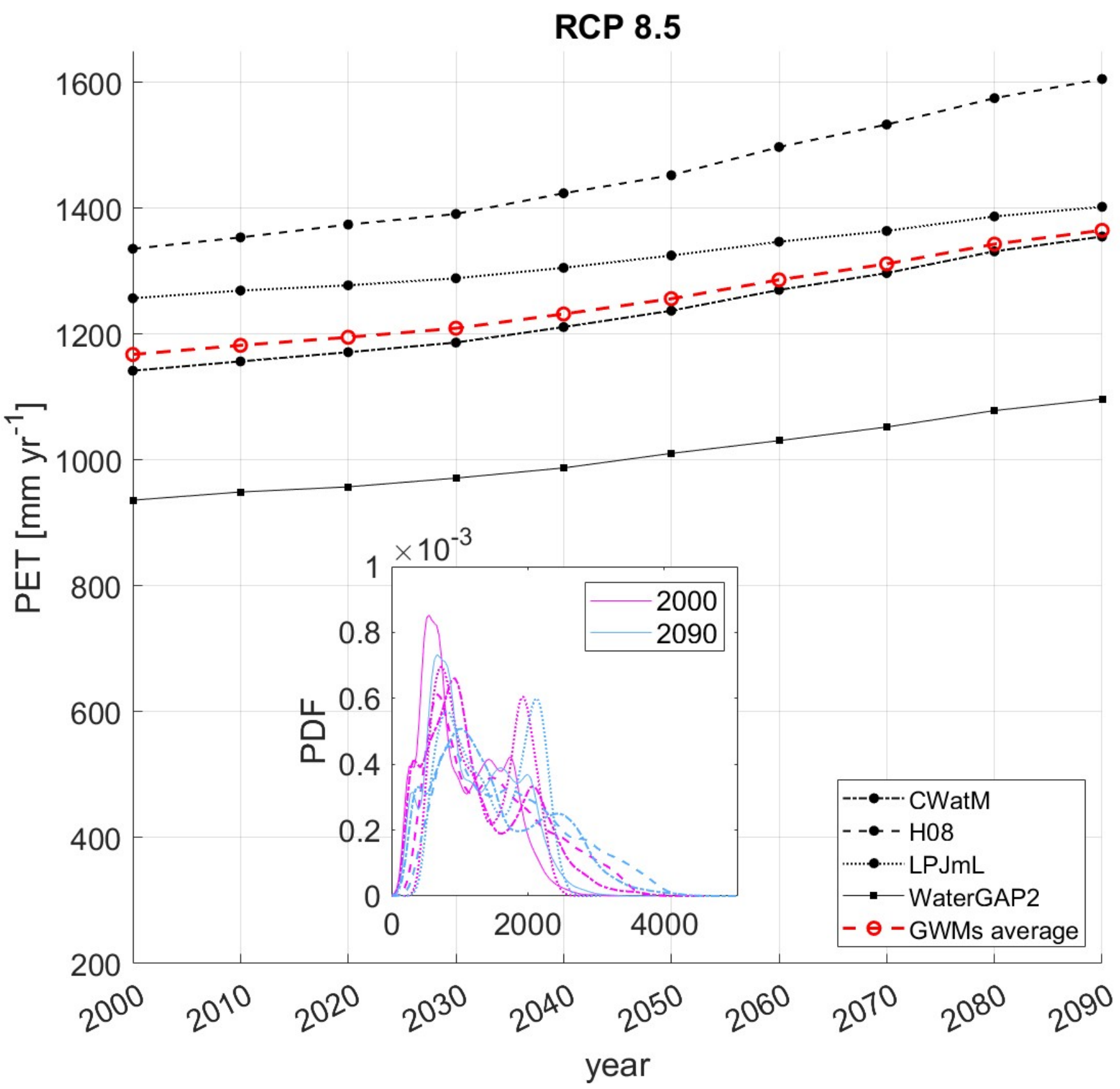

Fig. S8: Mean global total annual potential evapotranspiration (PET) derived from the four water models (GWMs) reported in the legend. Inset shows the probability density functions (PDF) of annual PET values in each grid cell for years 2000 and 2090, under RCP 8.5 for different GWMs (estimates not available for the models MATSIRO and PCR-GLOBWB for this scenario – Tab. 1 of the main text).

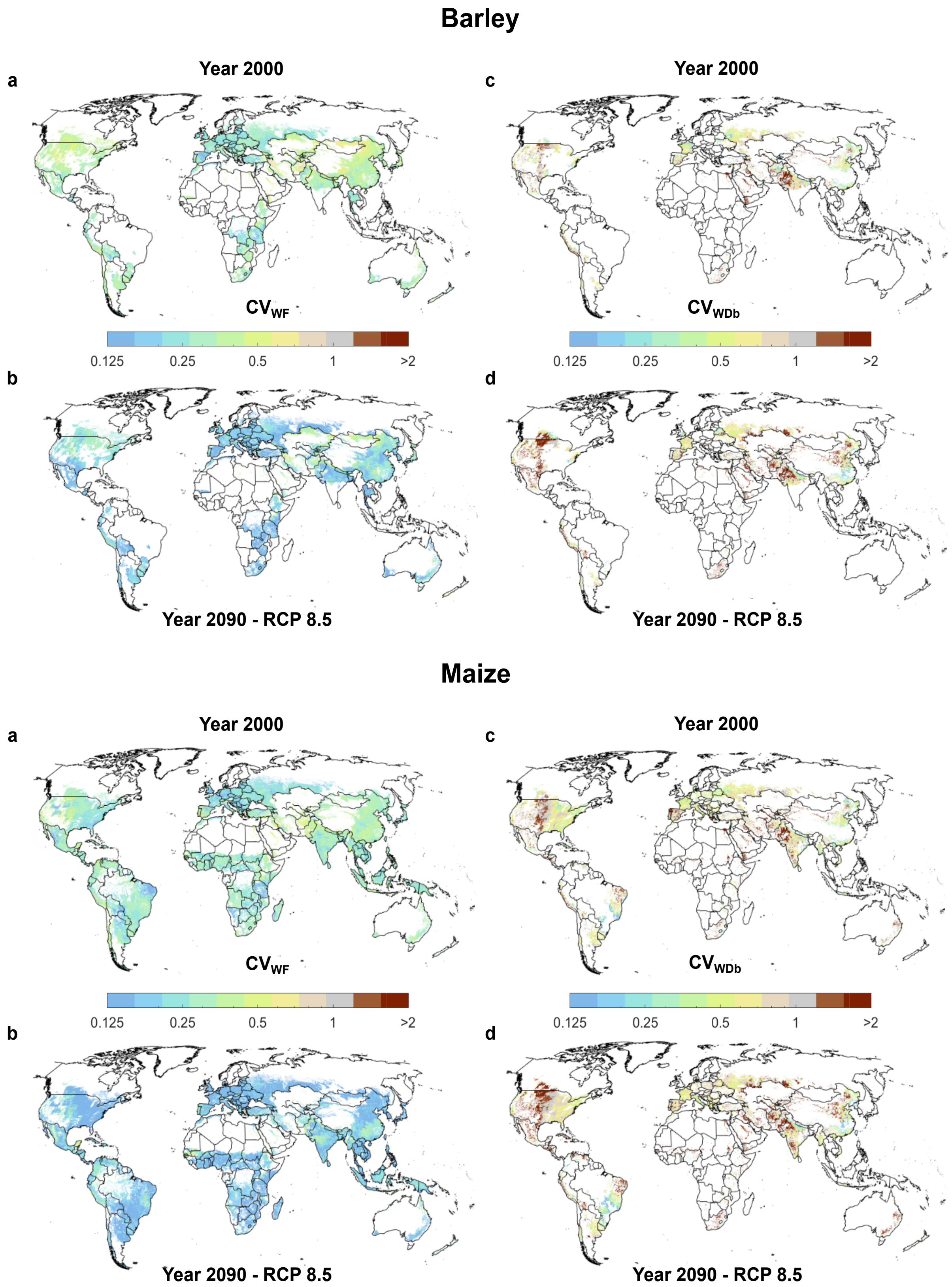

Fig. S9: Coefficient of variation *CV* of total water footprint (WF) (**a,b**) and blue water debt WD_b (**c,d**) for barley and maize (years 2000 and 2090 – RCP 8.5).

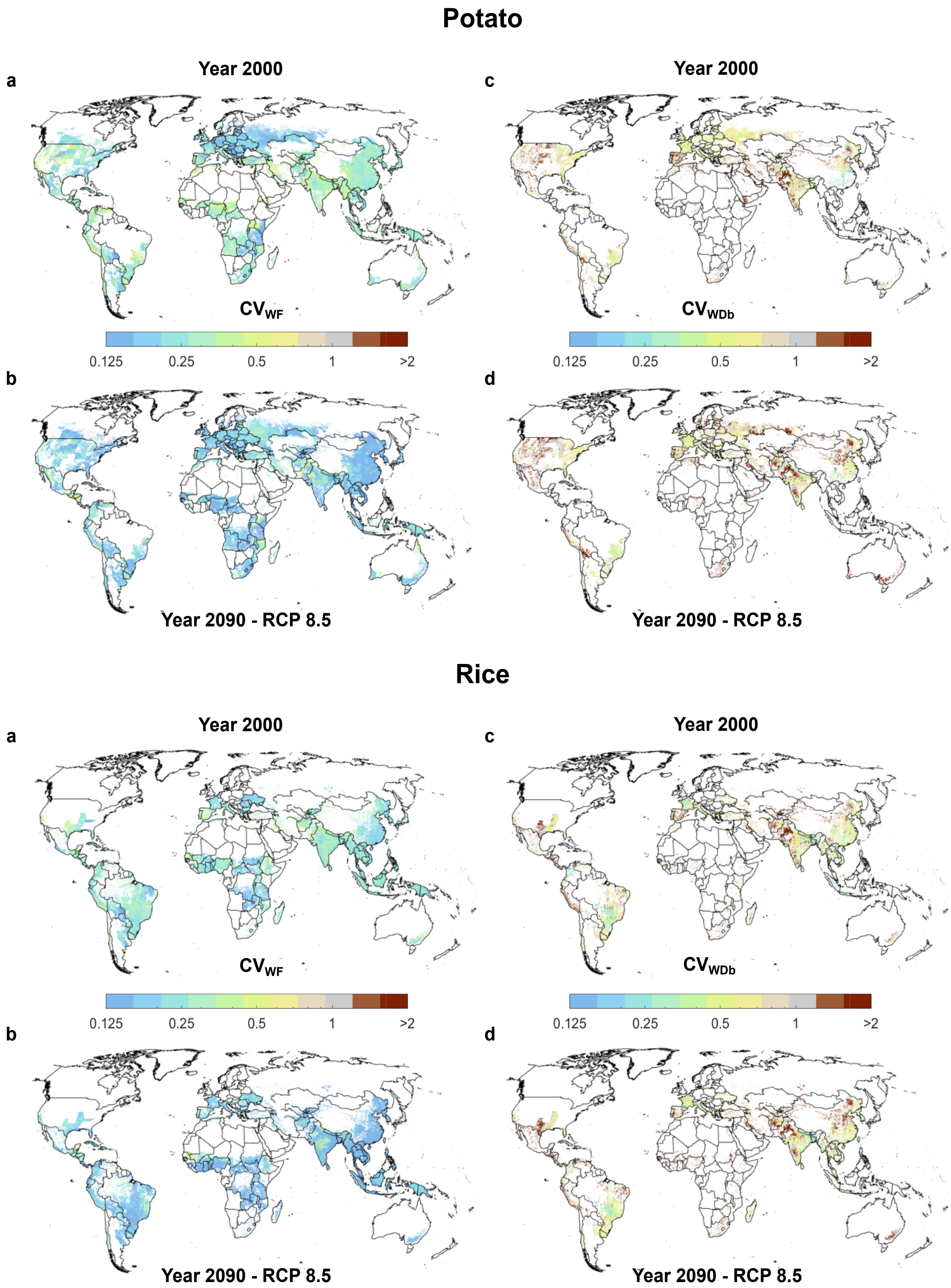

Fig. S10: Coefficient of variation CV of total water footprint (WF) (**a,b**) and blue water debt $\mathrm{WD_b}$ (**c,d**) for potato and rice (years 2000 and 2090 – RCP 8.5).

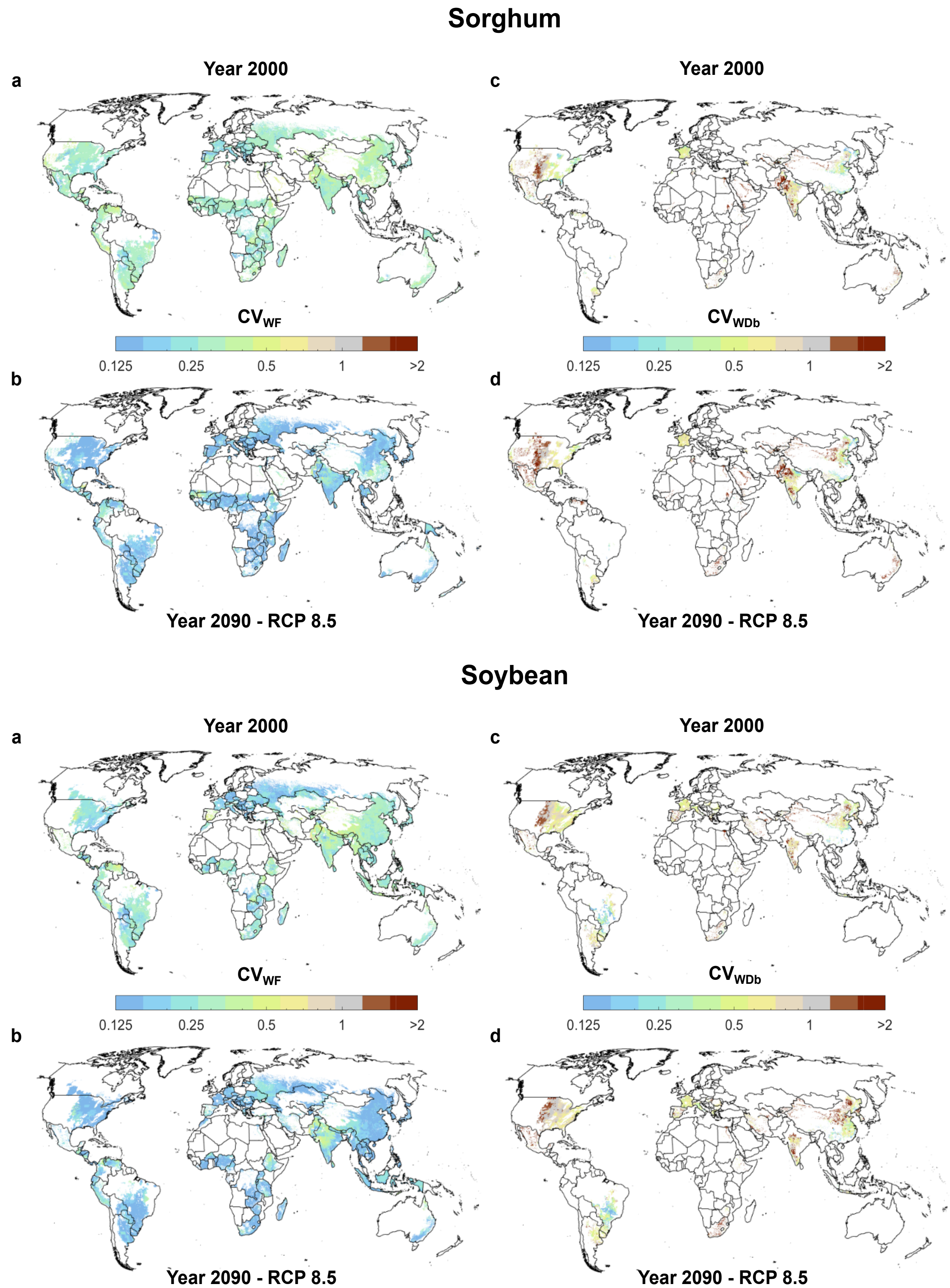

Fig. S11: Coefficient of variation CV of total water footprint (WF) (**a,b**) and blue water debt $\mathrm{WD_b}$ (**c,d**) for sorghum and soybean (years 2000 and 2090 – RCP 8.5).

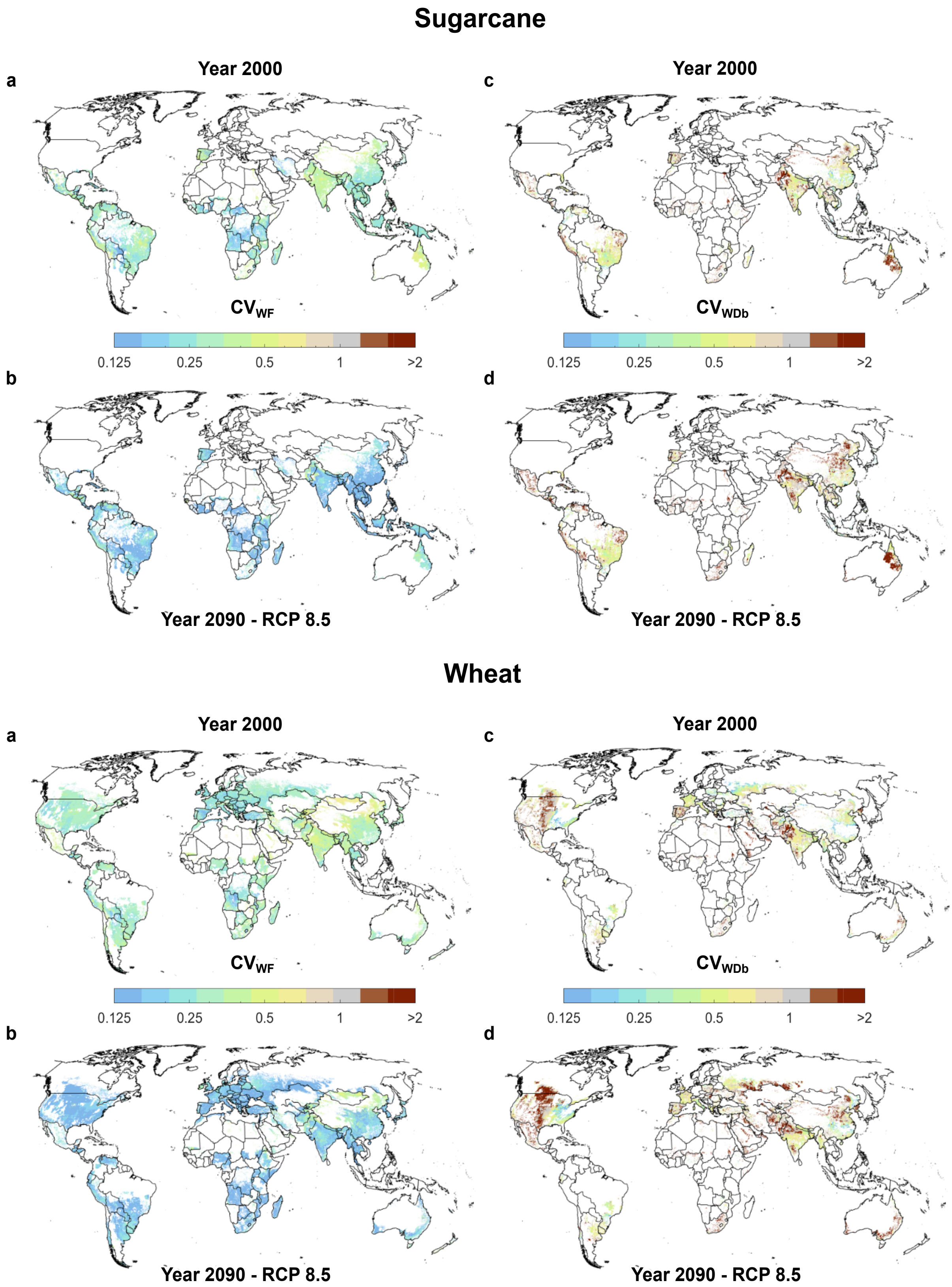

Fig. S12: Coefficient of variation *CV* of total water footprint (WF) (**a,b**) and blue water debt WD_b (**c,d**) for sugarcane and wheat (years 2000 and 2090 – RCP 8.5).

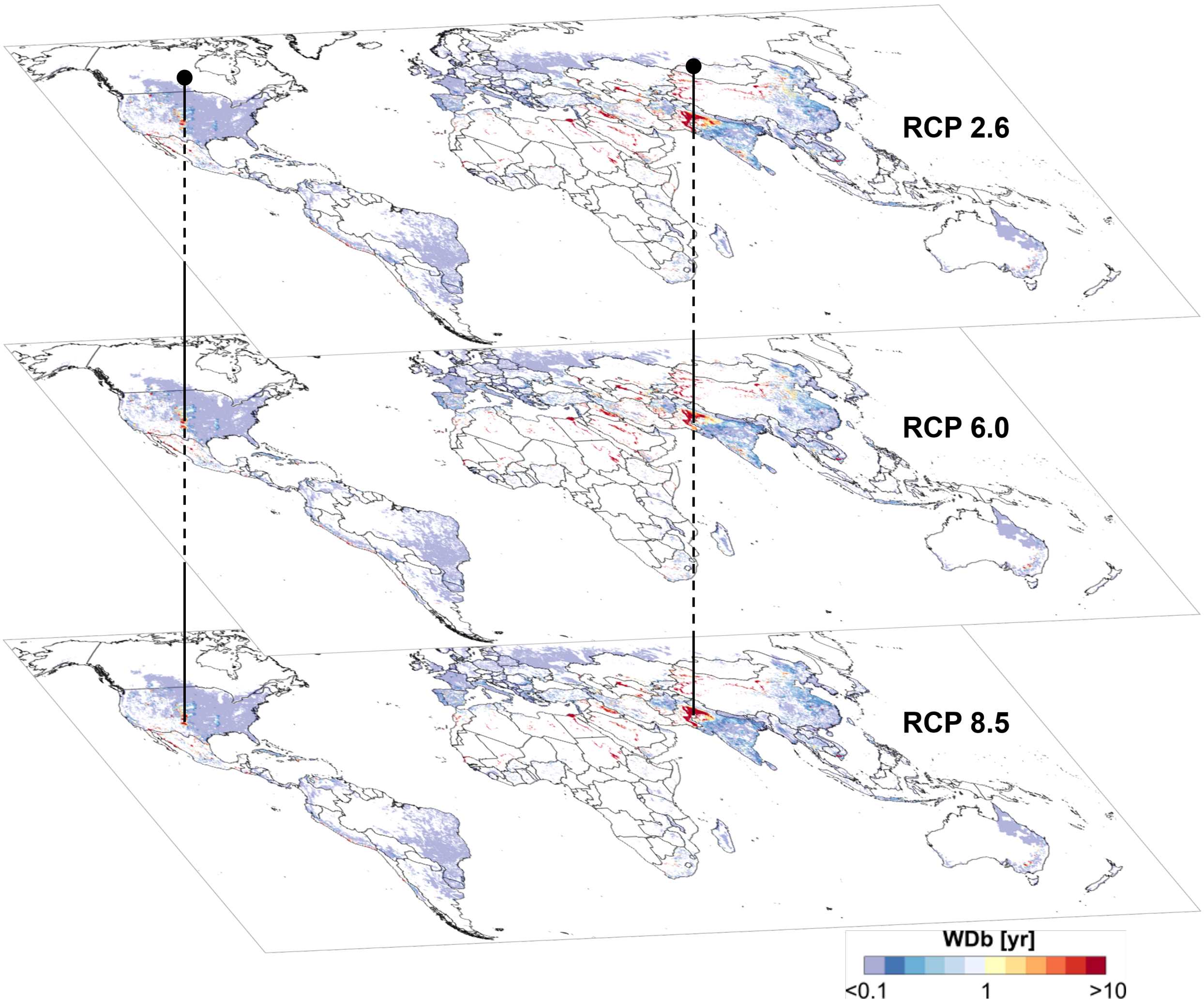

Fig. S13: Projected end-of-century (2090) blue water debt WD_b under the three climate change scenarios, for all crops and averaged across all climate and water models. The black lines indicate the overlapping of the Asian and American hotspots (i.e. characterized by $WD_b > 1$ year) across different RCPs.

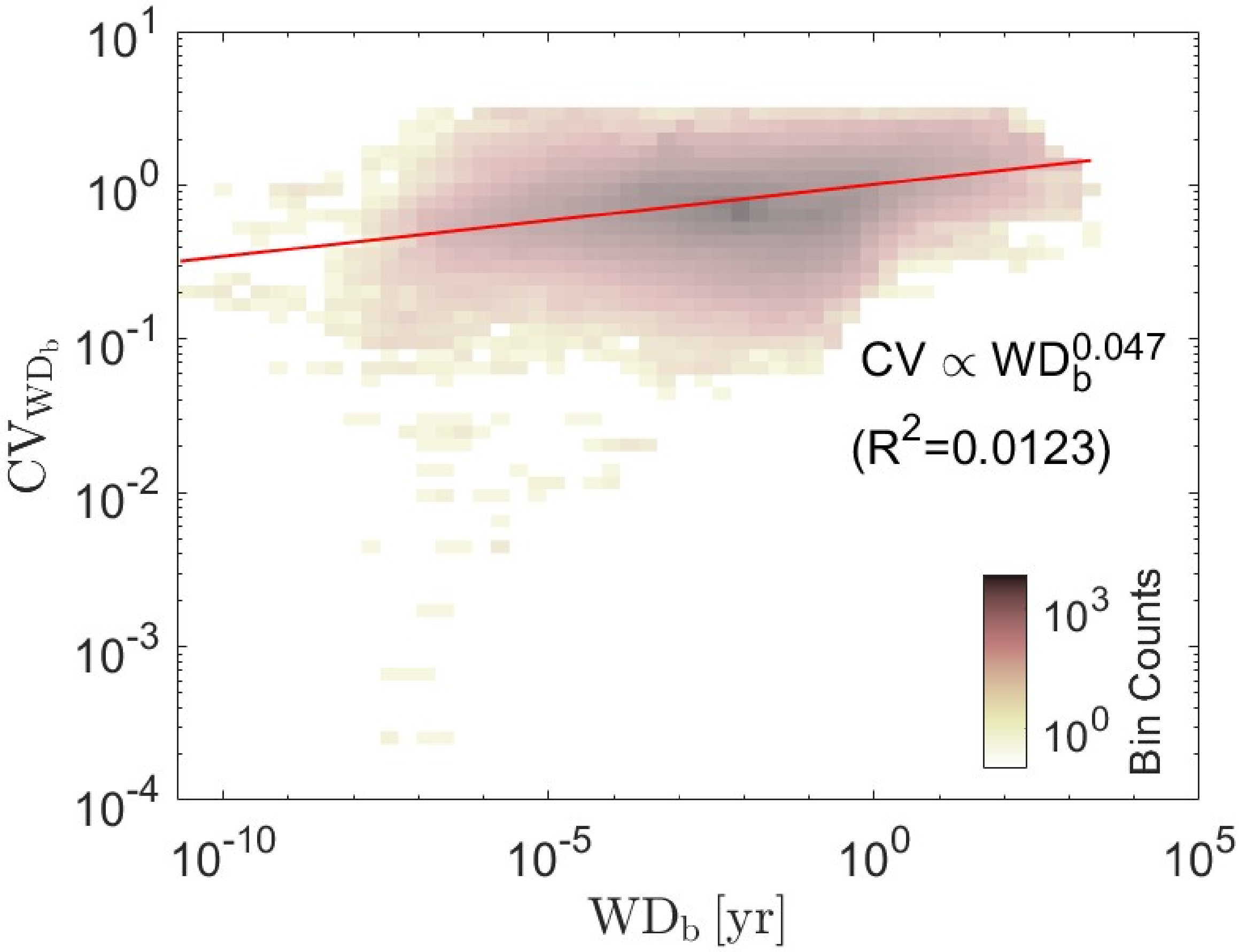

Fig. S14: Bi-logarithmic grid-cell relationship between the blue water debt (WD_b) and its associated coefficient of variation (CV), binned to increase readability (colors code the number of data points in each bin). The red line represents the linear regression ($R^2 \approx 0.0123$) fitting the data.

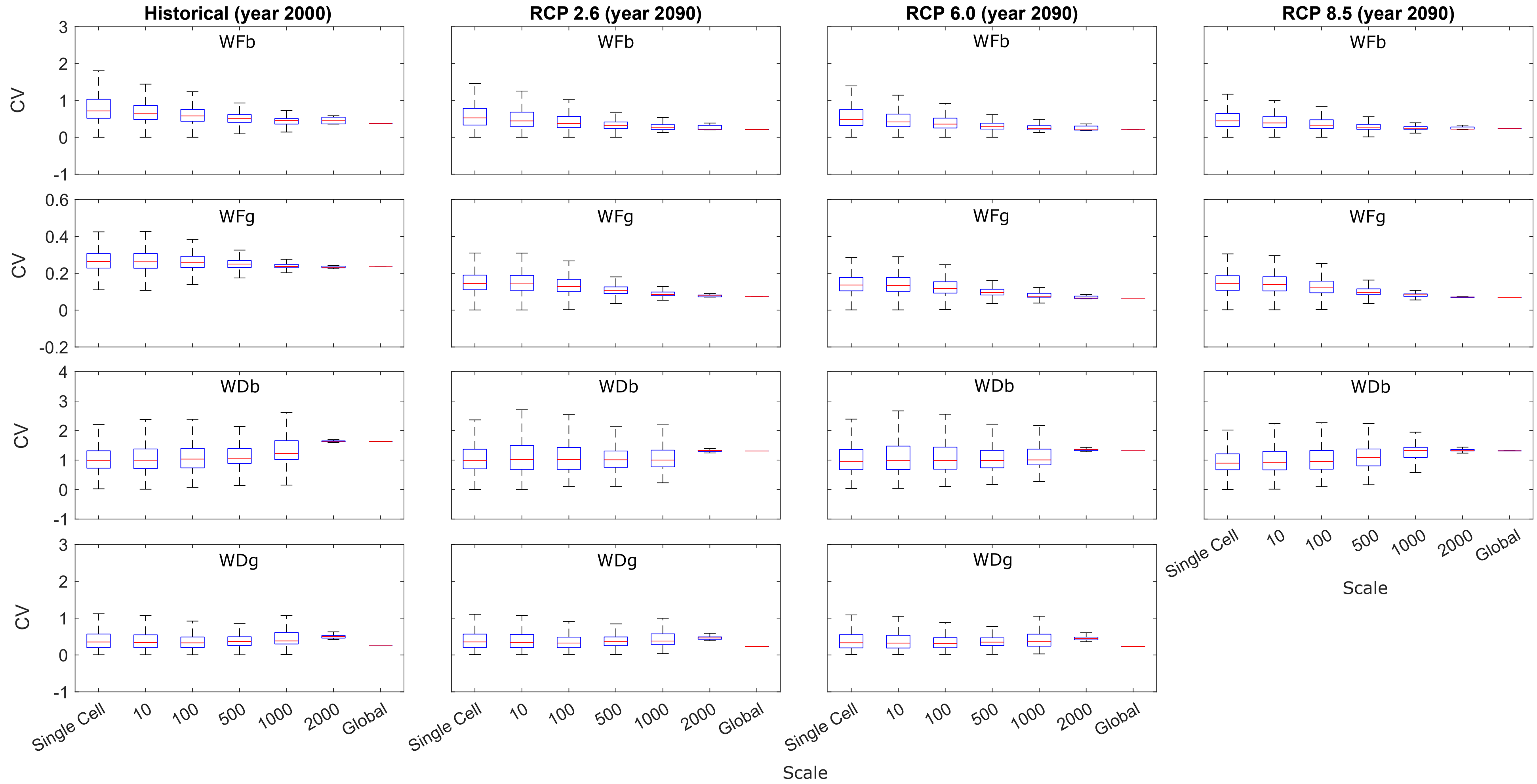

Fig. S15: Scale-dependency of the uncertainty. Total coefficient of variation among all water and climate models (for all the crops) as a function of the grid size (number of cells) considered, ranging from a single grid cell up to the to global scale.

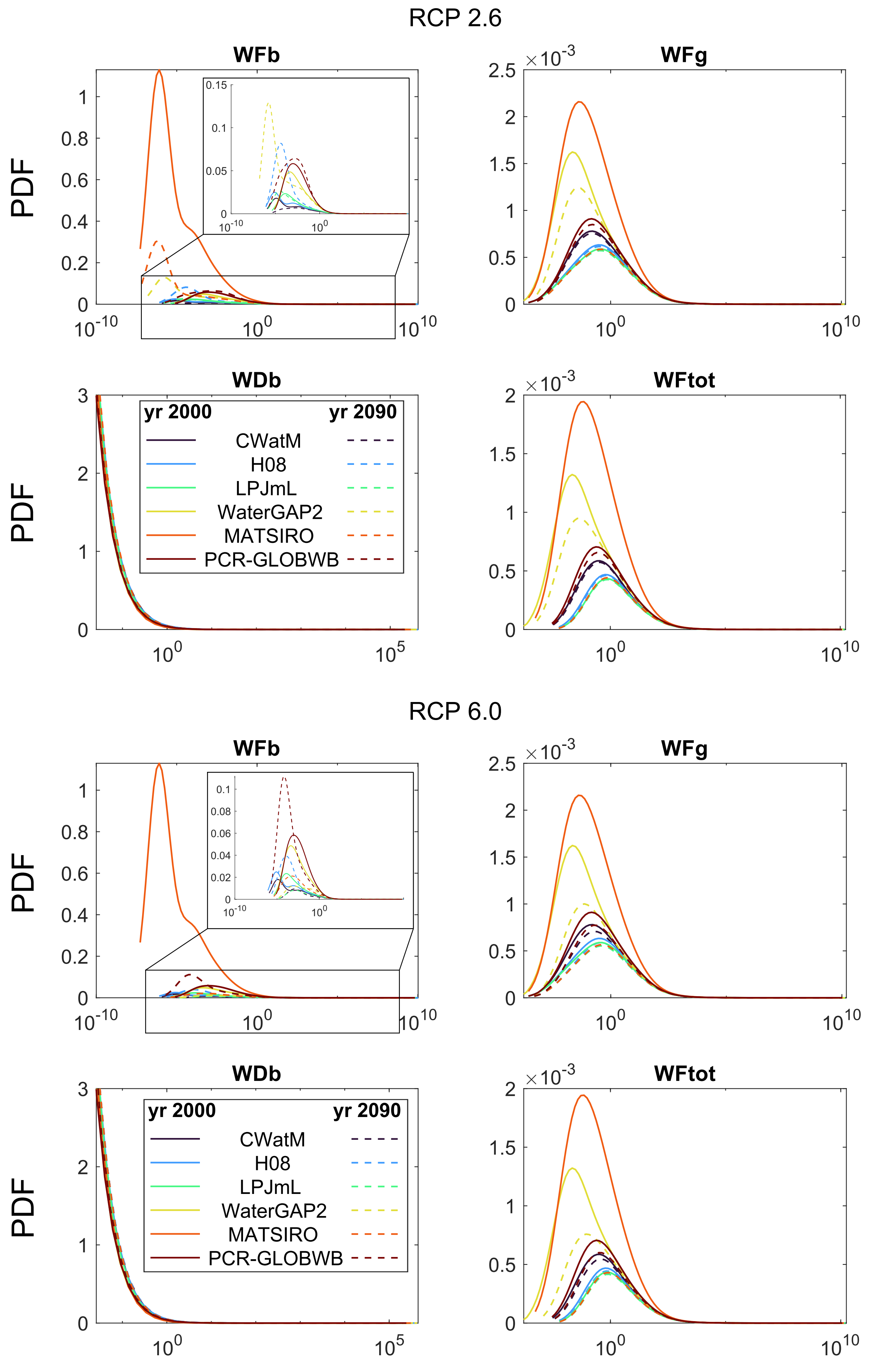

Fig. S16: Probability density functions (PDFs) of blue and green water footprints (WF_b and WF_g, respectively, in m^3), blue water debt (WD_b, $years$), and total water footprint (WF_{tot}, m^3). Colors code the six GWMs, with solid lines indicating the year 2000, while dashed lines refer to the end-of-century (year 2090) projections, under RCP 2.6 and 6.0.

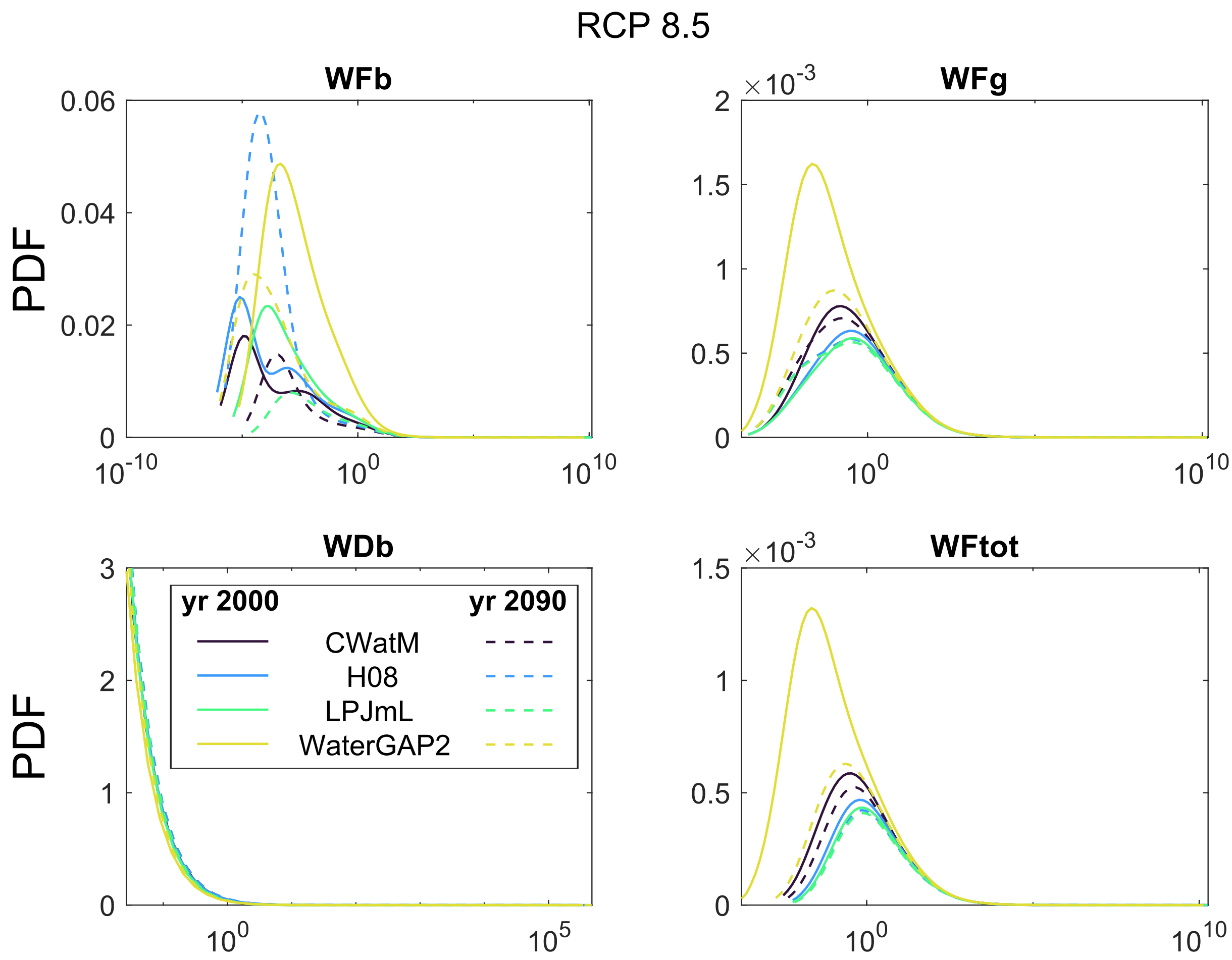

Fig. S17: Probability density functions (PDFs) of blue and green water footprints ($\mathrm{WF_b}$ and $\mathrm{WF_g}$, respectively, in m^3), blue water debt ($\mathrm{WD_b}$, *years*), and total water footprint ($\mathrm{WF_{tot}}$, m^3). Colors code the four water models that are available for the worst-case scenario RCP 8.5, with solid lines indicating the year 2000, while dashed lines refer to the end-of-century (year 2090) projections.

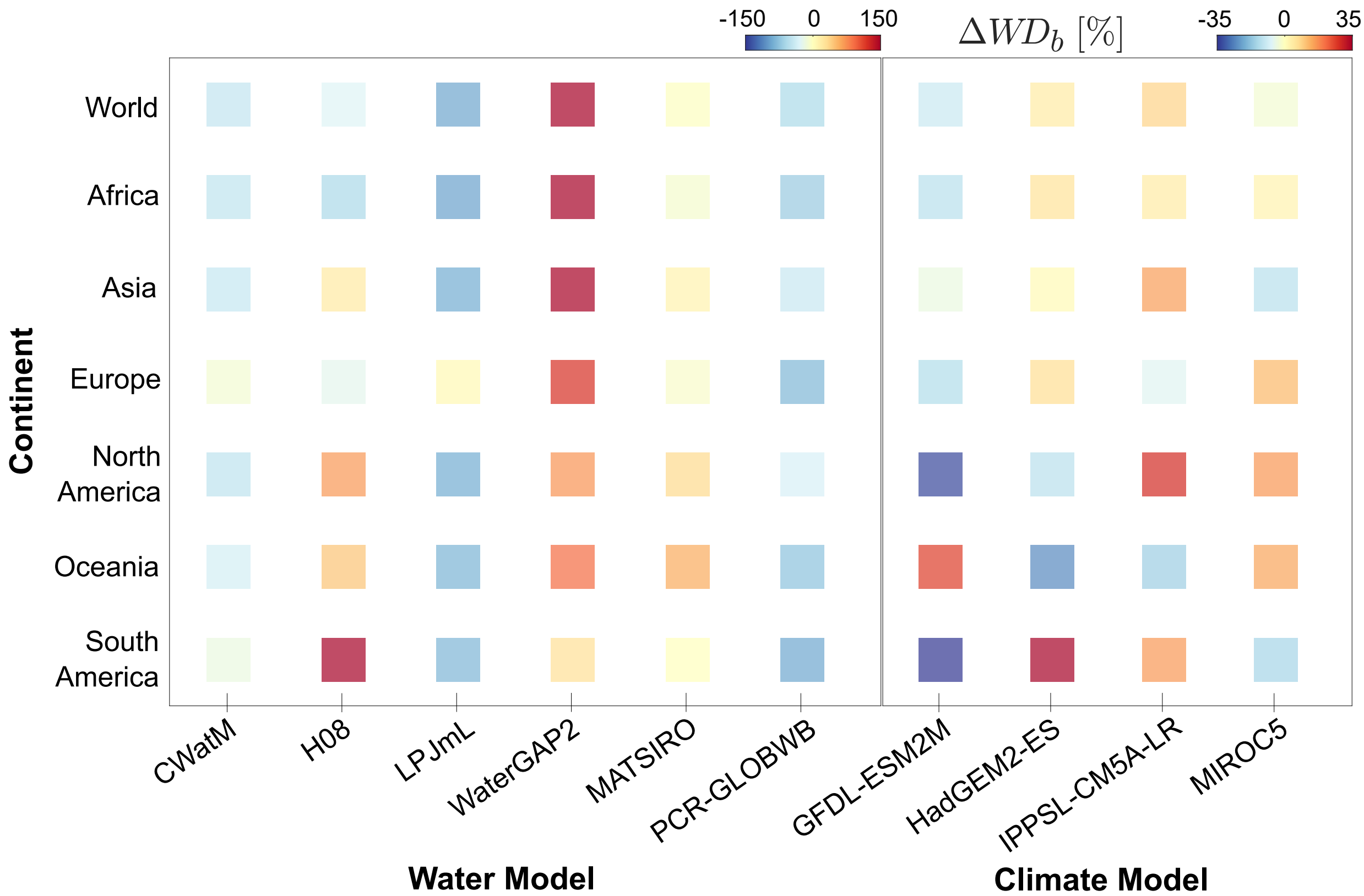

Fig. S18: Sources of uncertainty in global and continental scale crop blue water debt. Variability of global and continental scale blue water debt ($\mathrm{WD_b}$) across GWMs (left panel) and GCMs (right panel) averaged for all years between 2000 and 2090 and all crops (here future periods refer to RCP 6.0, for which all models are available – Tab. 1 of the main text). Colors code the relative deviation of $\mathrm{WD_b}$ with respect to the average value per continent C, that is, $\Delta\mathrm{WD_b} = [(\mathrm{WD_b}_{C,m} - \overline{\mathrm{WD_b}}_C)/\overline{\mathrm{WD_b}}_C] \cdot 100$, m being the considered model.

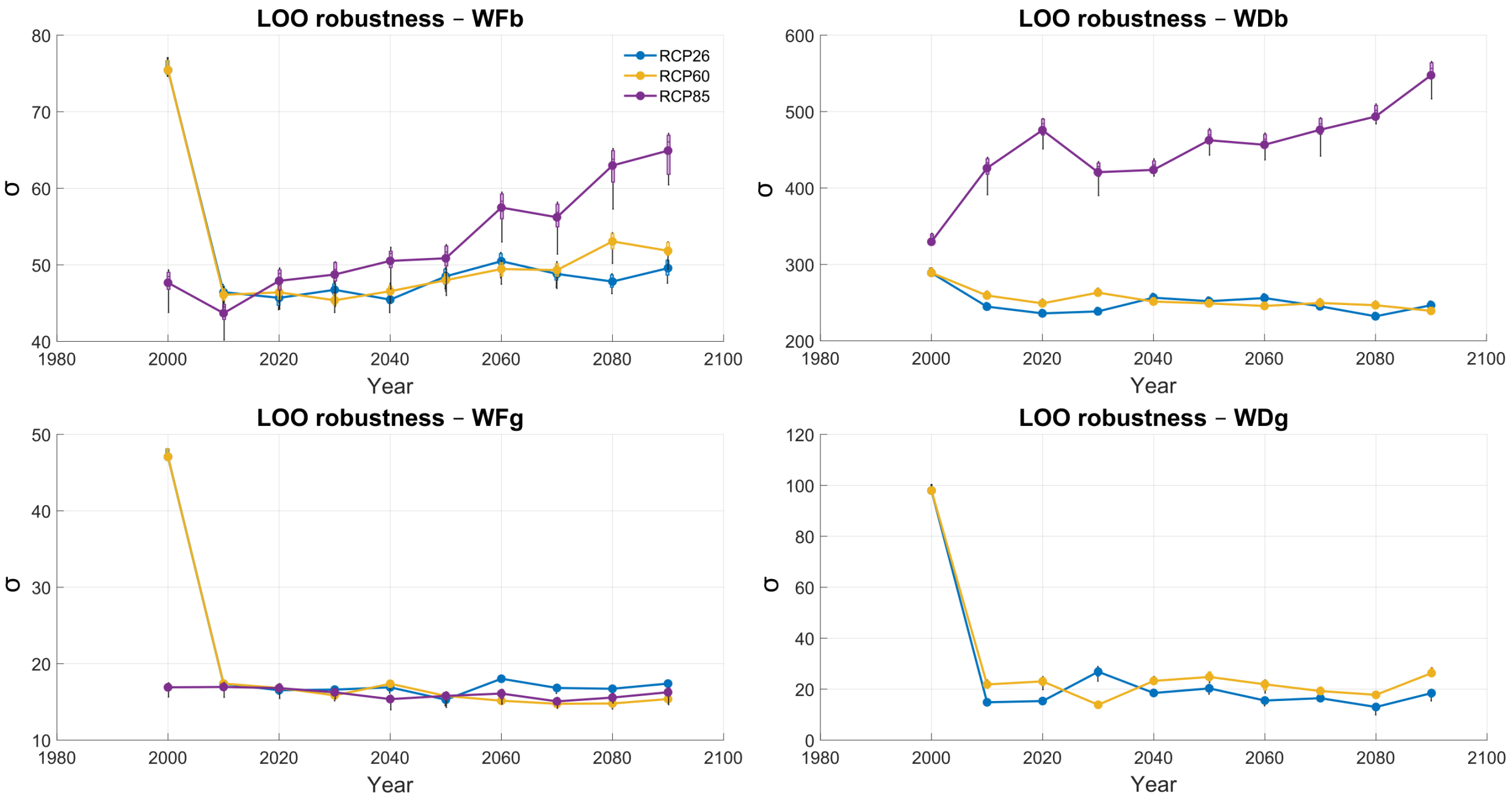

Fig. S19: Robustness of the uncertainty metric, namely the standard deviation relative to year 2000 (σ, %), assessed through a leave-one-out (LOO) analysis. Panels show results for WF_b (top left), WF_g (bottom left), WD_b (top right), and WD_g (bottom right), under RCPs 2.6 (blue), 6.0 (yellow), and 8.5 (purple). The continuous line represents the standard deviation computed using the full ensemble of models, while the error bars depict the distribution of uncertainty estimates obtained by sequentially excluding one model at a time. The limited spread of the boxplots relative to the reference line indicates that the estimated uncertainty remains stable when individual models are omitted. The maximum variability is found for WF_b in the year 2090 under RCP 8.5 (median 66.06%, quartiles 61.82% and 66.92%).

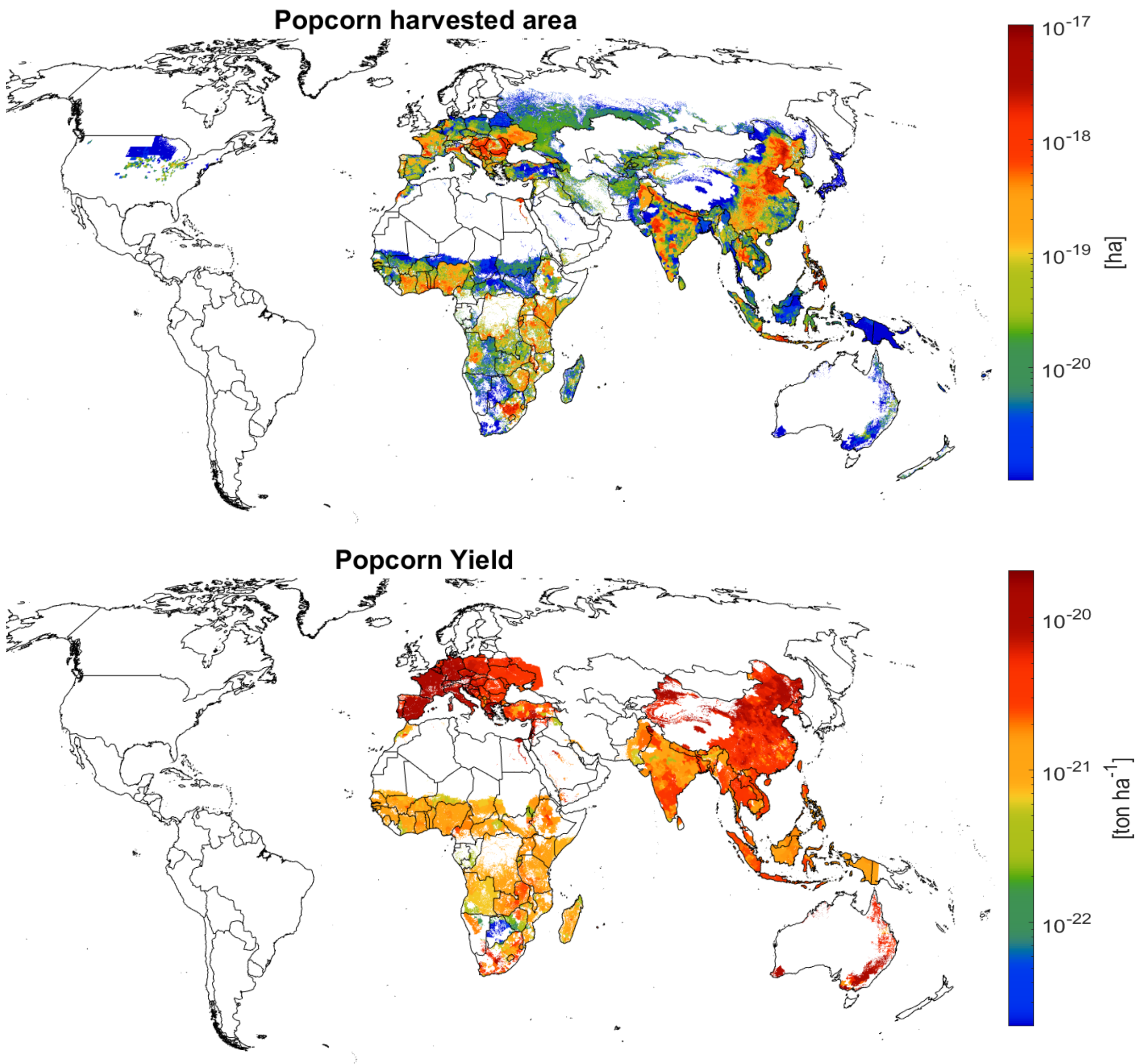

Fig. S20: Global harvested areas in hectares (top) and yield in ton per hectares (bottom) for the Maize's sub-class popcorn (data from Earthstat [4]).

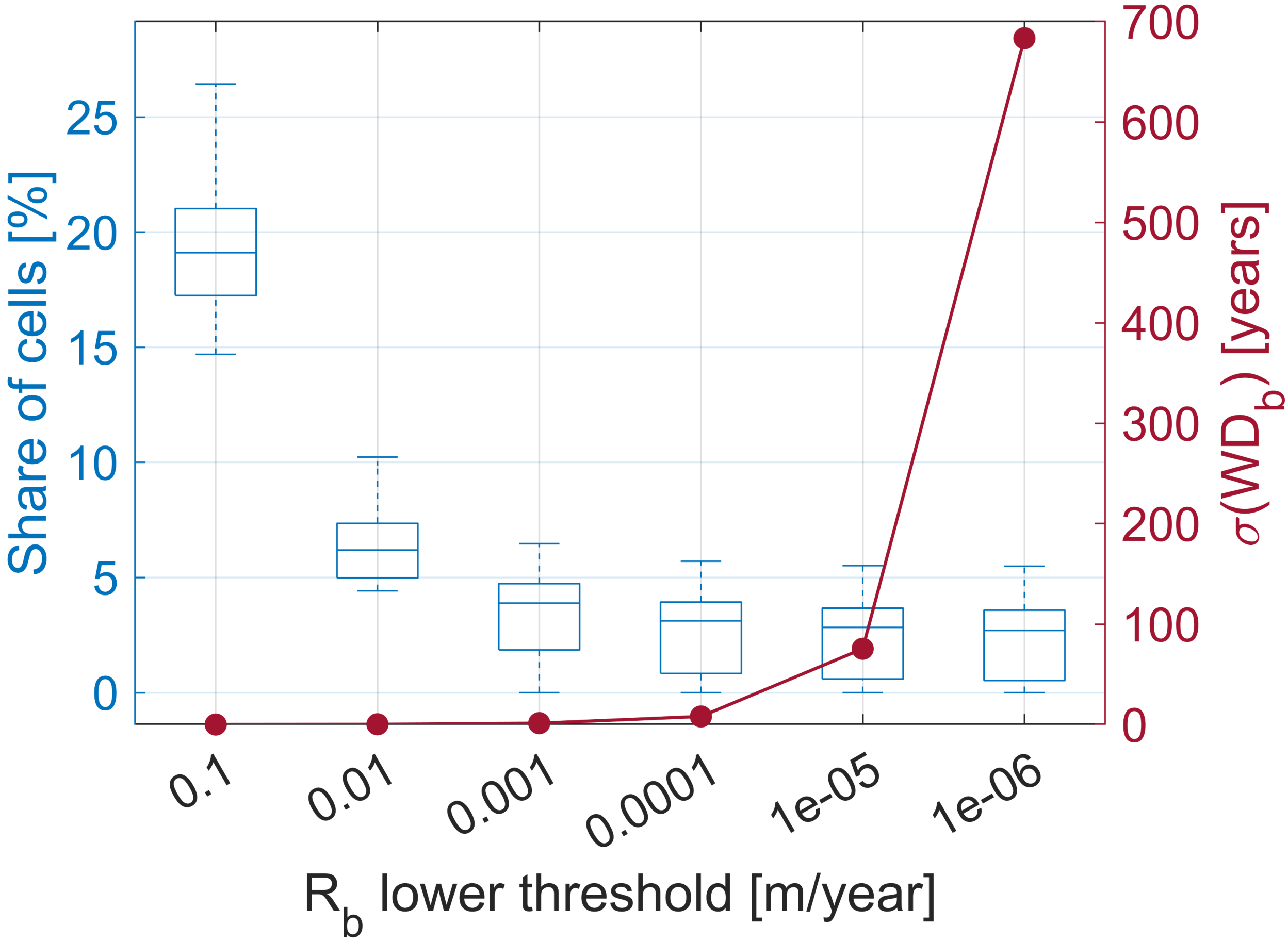

Fig. S21: Sensitivity of blue water debt to minimum thresholds applied to water renewability rates. Unrealistically low values of surface runoff and groundwater recharge were filtered using a range of thresholds (x-axis). For each threshold, the percentage of global cells affected by each value (left y-axis, in blue), and the variability of WD_b (expressed as standard deviation – right y-axis, in red) were evaluated. Results are aggregated across all crops, model combinations, and the entire 10-year period.

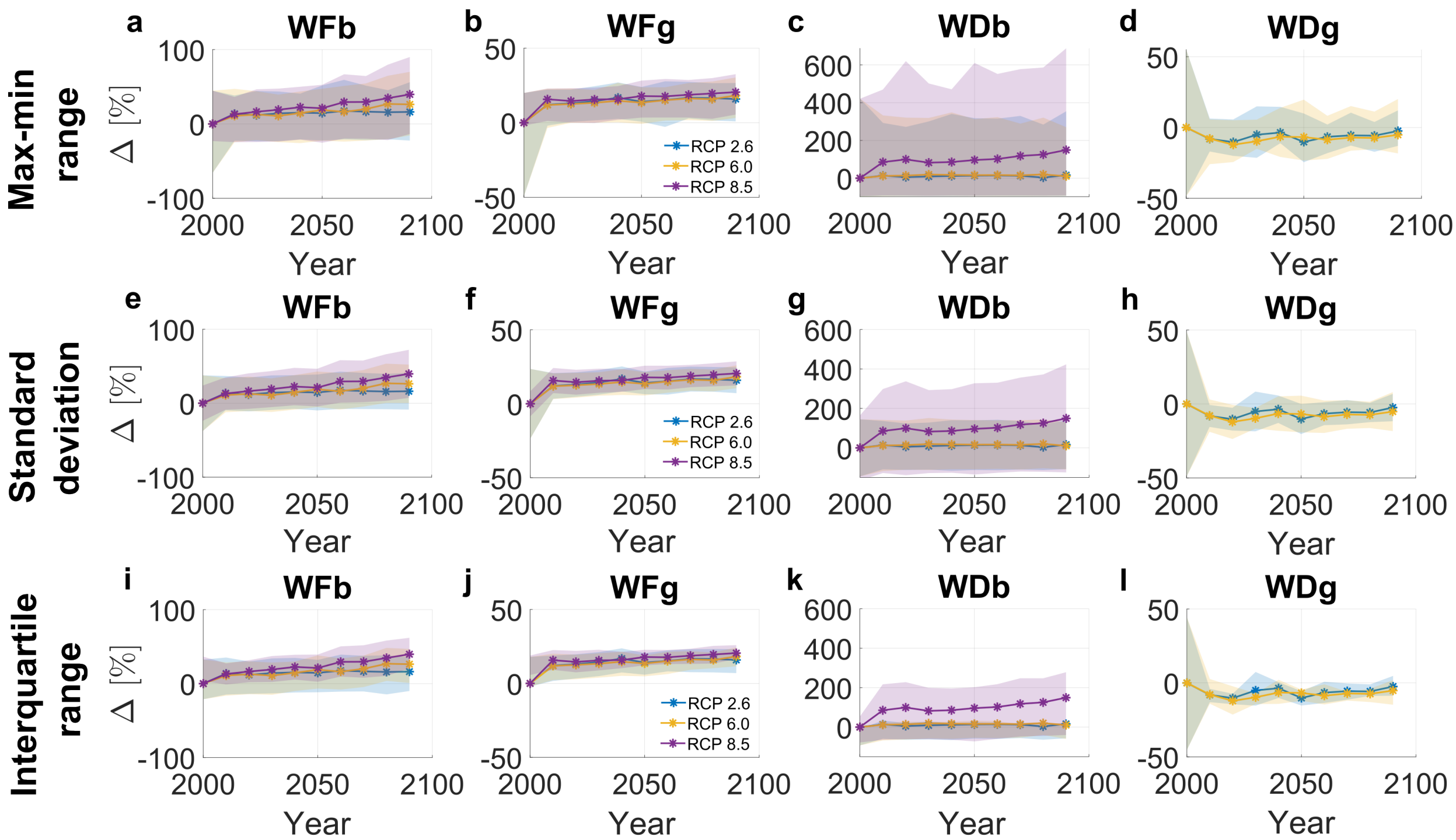

Fig. S22: Variability in projections of global water footprint and water debt estimates using different metrics. Temporal evolution of the percentage change Δ (relative to the average in the reference year 2000) of global (**a**) blue water footprint WF_b, (**b**) green water footprint WF_g, (**c**) blue water debt WD_b, and (**d**) green water debt WD_g accounting for all the 8 crops. Solid lines represent average values across all GWMs and GCMs under RCP 2.6 (blue), 6.0 (yellow), and 8.5 (purple). The uncertainty ranges denoting variability among values from all models (colored shaded areas) are computed as: the maximum-minimum difference (panels **a-d**), the standard deviation (panels **e-h**), and the interquartile range (panels **i-l**).

Supplementary References

[1] Mann, H. B. Nonparametric tests against trend. *Econometrica: Journal of the econometric society* 245–259 (1945).

[2] Kendall, M. G. Rank and product-moment correlation. *Biometrika* 177–193 (1949).

[3] Allen, R. G., Pereira, L. S., Raes, D., Smith, M. *et al.* Crop evapotranspiration-guidelines for computing crop water requirements-fao irrigation and drainage paper 56. *Fao, Rome* **300**, D05109 (1998).

[4] Monfreda, C., Ramankutty, N. & Foley, J. A. Farming the planet: 2. Geographic distribution of crop areas, yields, physiological types, and net primary production in the year 2000. *Global biogeochemical cycles* **22** (2008).
